# Best match graphs

**DOI:** 10.1007/s00285-019-01332-9

**Published:** 2019-04-09

**Authors:** Manuela Geiß, Edgar Chávez, Marcos González Laffitte, Alitzel López Sánchez, Bärbel M. R. Stadler, Dulce I. Valdivia, Marc Hellmuth, Maribel Hernández Rosales, Peter F. Stadler

**Affiliations:** 1 CONACYT-Instituto de Matemáticas, UNAM Juriquilla, Blvd. Juriquilla 3001, 76230 Juriquilla, Querétaro, QRO Mexico; 2 Centro de Ciencias Básicas, Universidad Autónoma de Aguascalientes, Av. Universidad 940, 20131 Aguascalientes, AGS Mexico; 3 Instituto de Matemáticas, UNAM Juriquilla, Blvd. Juriquilla 3001, 76230 Juriquilla, Querétaro, QRO Mexico; 4 Bioinformatics Group, Department of Computer Science, University of Leipzig, Härtelstraße 16-18, 04107 Leipzig, Germany; 5 Interdisciplinary Center of Bioinformatics, University of Leipzig, Härtelstraße 16-18, 04107 Leipzig, Germany; 6 Institute of Mathematics and Computer Science, University of Greifswald, Walther-Rathenau-Straße 47, 17487 Greifswald, Germany; 7 Center for Bioinformatics, Saarland University, Building E 2.1, P.O. Box 151150, 66041 Saarbrücken, Germany; 8 Max-Planck-Institute for Mathematics in the Sciences, Inselstraße 22, 04103 Leipzig, Germany; 9 German Centre for Integrative Biodiversity Research (iDiv) Halle-Jena-Leipzig, Leipzig University, Härtelstraße 16-18, 04107 Leipzig, Germany; 10 Competence Center for Scalable Data Services and Solutions, Leipzig University, Härtelstraße 16-18, 04107 Leipzig, Germany; 11 Leipzig Research Center for Civilization Diseases, Leipzig University, Härtelstraße 16-18, 04107 Leipzig, Germany; 12 Institute of Theoretical Chemistry, University of Vienna, Währingerstraße 17, 1090 Vienna, Austria; 13 Facultad de Ciencias, Universidad National de Colombia, Sede Bogotá, Colombia; 14 Santa Fe Institute, 1399 Hyde Park Rd, Santa Fe, NM 87501 USA

**Keywords:** Phylogenetic combinatorics, Colored digraph, Reachable sets, Hierarchy, Hasse diagram, Rooted triples, Supertrees

## Abstract

Best match graphs arise naturally as the first processing intermediate in algorithms for orthology detection. Let *T* be a phylogenetic (gene) tree *T* and $\sigma$ an assignment of leaves of *T* to species. The best match graph $(G,\sigma )$ is a digraph that contains an arc from *x* to *y* if the genes *x* and *y* reside in different species and *y* is one of possibly many (evolutionary) closest relatives of *x* compared to all other genes contained in the species $\sigma (y)$. Here, we characterize best match graphs and show that it can be decided in cubic time and quadratic space whether $(G,\sigma )$ derived from a tree in this manner. If the answer is affirmative, there is a unique least resolved tree that explains $(G,\sigma )$, which can also be constructed in cubic time.

## Introduction

Symmetric best matches (Tatusov et al. 1997), also known as bidirectional best hits (BBH) (Overbeek et al. 1999), reciprocal best hits (RBH) (Bork et al. 1998), or reciprocal smallest distance (RSD) (Wall et al. 2003) are the most commonly employed method for inferring orthologs (Altenhoff and Dessimoz 2009; Altenhoff et al. 2016). Practical applications typically produce, for each gene from species *A*, a list of genes found in species *B*, ranked in the order of decreasing sequence similarity. From these lists, reciprocal best hits are readily obtained. Some software tools, such as ProteinOrtho (Lechner et al. 2011, 2014), explicitly construct a digraph whose arcs are the (approximately) co-optimal best matches. Empirically, the pairs of genes that are identified as reciprocal best hits depend on the details of the computational method for quantifying sequence similarity. Most commonly, blast or blat scores are used. Sometimes exact pairwise alignment algorithms are used to obtain a more accurate estimate of the evolutionary distance, see Moreno-Hagelsieb and Latimer (2008) for a detailed investigation. Independent of the computational details, however, reciprocal best match are of interest because they approximate the concept of pairs of *reciprocal evolutionarily most closely related* genes. It is this notion that links best matches directly to orthology: Given a gene *x* in species *a* (and disregarding horizontal gene transfer), all its co-orthologous genes *y* in species *b* are by definition closest relatives of *x*.

Evolutionary relatedness is a phylogenetic property and thus is defined relative to the phylogenetic tree *T* of the genes under consideration. More precisely, we consider a set of genes *L* (the leaves of the phylogenetic tree *T*), a set of species *S*, and a map $\sigma$ assigning to each gene $x\in L$ the species $\sigma (x)\in S$ within which it resides. A gene *x* is more closely related to gene *y* than to gene *z* if ${{\,\mathrm{lca}\,}}(x,y)\prec {{\,\mathrm{lca}\,}}(x,z)$. As usual, ${{\,\mathrm{lca}\,}}$ denotes the last common ancestor, and $p\prec q$ denotes the fact that *q* is located above *p* along the path connecting *p* with the root of *T*. The partial order $\preceq$ (which also allows equality) is called the ancestor order on *T*. We can now make the notion of a *best match* precise:

### Definition 1

Consider a tree *T* with leaf set *L* and a surjective map $\sigma :L\rightarrow S$. Then $y\in L$ is a *best match* of $x\in L$, in symbols $x\mathrel {\rightarrow }y$, if and only if ${{\,\mathrm{lca}\,}}(x,y)\preceq {{\,\mathrm{lca}\,}}(x,y')$ holds for all leaves $y'$ from species $\sigma (y')=\sigma (y)$.

**Fig. 1:**
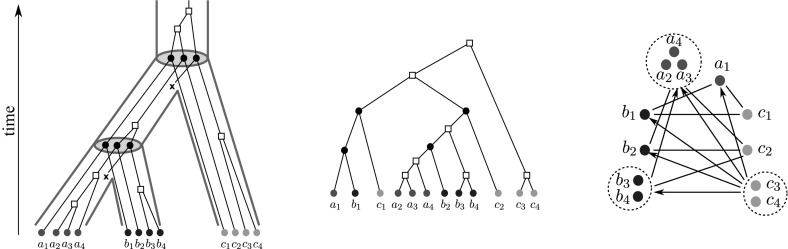
An evolutionary scenario (left) consists of a gene tree whose inner vertices are marked by the event type ($\bullet$ for speciations, $\square$ for gene duplications, and $\times$ for gene loss) together with its embedding into a species tree (drawn as tube-like outline). All events are placed on a time axis. The middle panel shows the observable part of the gene tree $(T,\sigma )$; it is obtained from the gene tree in the full evolutionary scenario by removing all leaves marked as loss events and suppression of all resulting degree two vertices (Hernandez-Rosales et al. 2012; Hellmuth 2017). The r.h.s. panel shows the colored best match graph $(G,\sigma )$ that is explained by $(T,\sigma )$. Directed arcs indicate the best match relation $\mathrel {\rightarrow }$. Bi-directional best matches ($x\mathrel {\rightarrow }y$ and $y\mathrel {\rightarrow }x$) are drawn as solid lines without arrow heads instead of pairs of arrows. Dotted circles collect sets of leaves that have the same in- and out-neighborhood. The corresponding arcs are shown only once

In order to understand how best matches (in the sense of Definition 1) are approximated by best hits computed by mean sequence similarity we first observe that best matches can be expressed in terms of the evolutionary time. Denote by *t*(*x*, *y*) the temporal distance along the evolutionary tree, as in Fig. 1. By definition *t*(*x*, *y*) is twice the time elapsed between ${{\,\mathrm{lca}\,}}(x,y)$ and *x* (or *y*), assuming that all leaves of *T* live in the present. Instead of Definition 1 we can then use “$x\mathrel {\rightarrow }y$ holds if and only if $t(x,y)\le t(x,y')$ for all $y'$ with $\sigma (y')=\sigma (y)\ne \sigma (x)$.” Mathematically, this is equivalent to Definition 1 whenever *t* is an ultrametric distance on *T*. For the temporal distance *t* this is the case. Best match heuristics therefore assume (often tacitly) that the *molecular clock hypothesis* (Zuckerkandl and Pauling 1962; Kumar 2005) is at least a reasonable approximation.

While this strong condition is violated more often than not, best match heuristics still perform surprisingly well on real-life data, in particular in the context of orthology prediction (Wolf and Koonin 2012). Despite practical problems, in particular in applications to Eukaryotic genes (Dalquen and Dessimoz 2013), reciprocal best heuristics perform at least as good for this task as methods that first estimate the gene phylogeny (Altenhoff et al. 2016; Setubal and Stadler 2018). One reason for their resilience is that the identification of best matches only requires inequalities between sequence similarities. In particular, therefore they are invariant under monotonic transformations and, in contrast e.g. to distance based phylogenetic methods, does not require additivity. Even more generally, it suffices that the evolutionary rates of the different members of a gene family are roughly the same within each lineage.

Best match methods are far from perfect, however. Large differences in evolutionary rates between paralogs, as predicted by the DDC model (Force et al. 1999), for example, may lead to false negatives among co-orthologs and false positive best matches between members of slower subfamilies. Recent orthology detection methods recognize the sources of error and complement sequence similarity by additional sources of information. Most notably, synteny is often used to support or reject reciprocal best matches (Lechner et al. 2014; Jahangiri-Tazehkand et al. 2017). Another class of approaches combine the information of small sets of pairwise matches to improve orthology prediction (Yu et al. 2011; Train et al. 2017). In the Concluding Remarks we briefly sketch a simple quartet-based approach to identify incorrect best match assignments.

Extending the information used for the correction of initial reciprocal best hits to a global scale, it is possible to improve orthology prediction by enforcing the global cograph of the orthology relation (Hellmuth et al. 2015; Lafond et al. 2016). This work originated from an analogous question: Can empirical reciprocal best match data be improved just by using the fact that ideally a best match relation should derive from a tree *T* according to Definition 1? To answer this question we need to understand the structure of best match relations.

The best match relation is conveniently represented as a colored digraph.

### Definition 2

Given a tree *T* and a map $\sigma :L\rightarrow S$, the *colored best match graph* (cBMG) $G(T,\sigma )$ has vertex set *L* and arcs $xy\in E(G)$ if $x\ne y$ and $x\mathrel {\rightarrow }y$. Each vertex $x\in L$ obtains the color $\sigma (x)$.

The rooted tree *Texplains* the vertex-colored graph $(G,\sigma )$ if $(G,\sigma )$ is isomorphic to the cBMG $G(T,\sigma )$.

To emphasize the number of colors used in $G(T,\sigma )$, that is, the number of species in *S*, we will write |*S*|-cBMG.

**Fig. 2:**
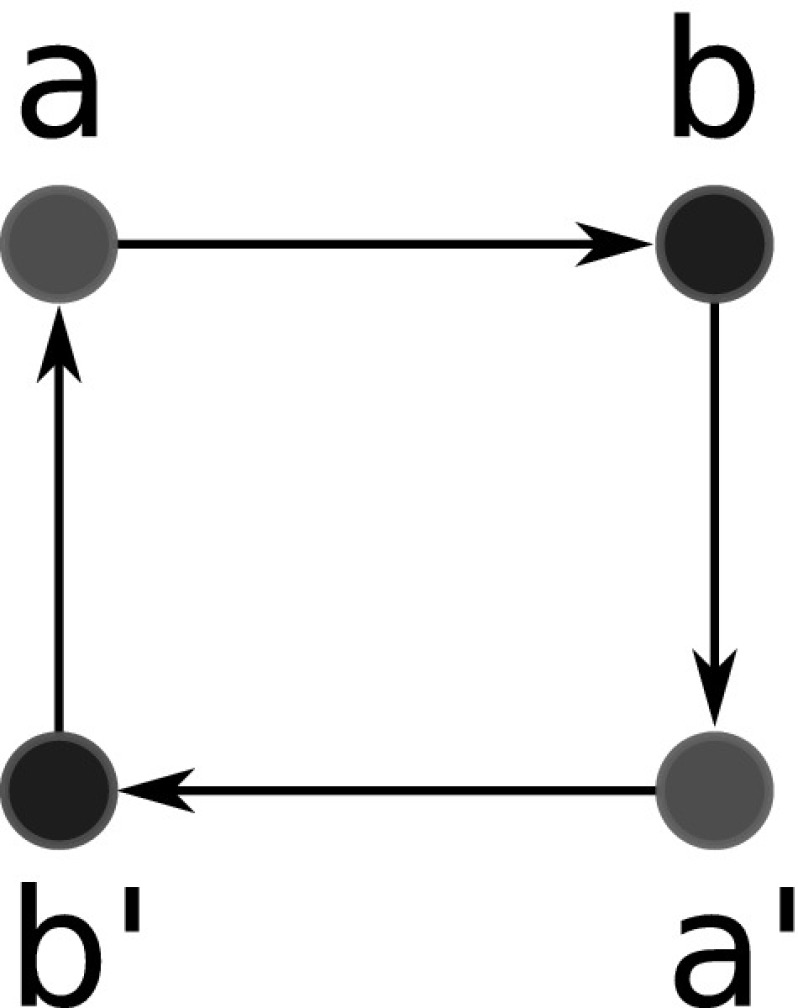
Not every graph with non-empty out-neighborhoods is is a colored best match graph. The 4-vertex graph $(G,\sigma )$ shown here is the smallest connected counterexample: there is no leaf-colored tree $(T,\sigma )$ that explains $(G,\sigma )$

The purpose of this contribution is to establish a characterization of cBMGs as an indispensable prerequisite for any method that attempts to directly correct empirical best match data. After settling the notation we establish a few simple properties of cBMGs and show that key problems can be broken down to the connected components of 2-colored BMGs. These are considered in detail in Sect. 3. The characterization of 2-BMGs is not a trivial task. Although the existence of at least one out-neighbor for each vertex is an obvious necessary condition, the example in Fig. 2 shows that it is not sufficient. In Sect. 3 we prove our main results on 2-cBMGs: the existence of a unique least resolved tree that explains any given 2-cBMG (Theorem 2), a characterization in terms of informative triples that can be extracted directly from the input graph (Theorem 6), and a characterization in terms of three simple conditions on the out-neighborhoods (Theorem 4). In Sect. 4 we provide a complete characterization of a general cBMG: It is necessary and sufficient that the subgraph induced by each pair of colors is a 2-cBMG and that the union of the triple sets of their least resolved tree representations is consistent. After a brief discussion of algorithmic considerations we close with a brief introduction into questions for future research.

## Preliminaries

### Notation

Given a rooted tree $T=(V,E)$ with root $\rho$, we say that a vertex $v\in V$ is an *ancestor* of $u\in V$, in symbols $u\preceq v$, *v* lies one the path from $\rho$ to *u*. For an edge $e=uv$ in the rooted tree *T* we assume that *u* is closer to the root of *T* than *v*. In this case, we call *v* a child of *u*, and *u* the parent of *v* and denote with $\mathsf {child}(u)$ the set of children of *u*. Moreover, $e=uv$ is an *outer edge* if $v\in L(T)$ and an *inner edge* otherwise. We write *T*(*v*) for the subtree of *T* rooted at *v*, $L(T')$ for the leaf set of some subtree $T'$ and $\sigma (L')=\{\sigma (x)\mid x\in L'\}$. To avoid dealing with trivial cases we will assume that $\sigma (L)=S$ contains at least two distinct colors. Furthermore, for $|S|=1$, the edge-less graphs are explained by any tree. Hence, we will assume $|S|\ge 2$ in the following. Without loosing generality we may assume throughout this contribution that all trees are phylogenetic, i.e., all inner vertices of *T* (except possibly the root) have at least two children. A tree is binary if each inner vertex has exactly two children.

We follow the notation used e.g. in Semple and Steel (2003) and say that $T'$ is *displayed* by *T*, in symbols $T'\le T$, if the tree $T'$ can be obtained from a subtree of *T* by contraction of edges. In addition, we will consider trees *T* with a coloring map $\sigma : L(T)\rightarrow S$ of its leaves, in short $(T,\sigma )$. We say that $(T,\sigma )$*displays* or *is a refinement of*$(T',\sigma ')$, whenever $T'\le T$ and $\sigma (v)=\sigma '(v)$ for all $v\in L(T')$.

We write $T_{L'}$ for the *restriction* of *T* to a subset $L'\subseteq L$. We denote by ${{\,\mathrm{lca}\,}}(A)$ the last common ancestor of all elements of any set *A* of vertices in *T*. For later reference we note that ${{\,\mathrm{lca}\,}}(A\cup B)={{\,\mathrm{lca}\,}}({{\,\mathrm{lca}\,}}(A),{{\,\mathrm{lca}\,}}(B))$. We sometimes write ${{\,\mathrm{lca}\,}}_T$ instead of ${{\,\mathrm{lca}\,}}$ to avoid ambiguities. We will often write $A \preceq x$, in case that ${{\,\mathrm{lca}\,}}(A)\preceq x$ and therefore, that *x* is an ancestor of all $a\in A$.

A binary tree on three leaves is called a *triple*. In particular, we write *xy*|*z* for the triple on the leaves *x*, *y* and *z* if the path from *x* to *y* does not intersect the path from *z* to the root. We write *r*(*T*) for the set of all triples that are displayed by the tree *T*. In particular, we call a triple set *Rconsistent* if there exists a tree *T* that displays *R*, i.e., $R\subseteq r(T)$. A rooted triple $xy|z\in r(T)$*distinguishes* an edge (*u*, *v*) in *T* if and only if *x*, *y* and *z* are descendants of *u*, *v* is an ancestor of *x* and *y* but not of *z*, and there is no descendant $v'$ of *v* for which *x* and *y* are both descendants. In other words, the edge (*u*, *v*) is distinguished by $xy|z\in r(T)$ if ${{\,\mathrm{lca}\,}}(x,y)=v$ and ${{\,\mathrm{lca}\,}}(x,y,z)=u$.

By a slight abuse of notation we will retain the symbol $\sigma$ also for the restriction of $\sigma$ to a subset $L'\subseteq L$. We write $L[s]=\{x\in L \mid \sigma (x)=s\}$ for the color classes on the leaves of $(T,\sigma )$ and denote by $\overline{\sigma (x)}=S{\setminus } \{\sigma (x)\}$ the set of colors different from the color of the leaf *x*.

All (di-)graphs considered here do not contain loops, i.e., there are no arcs of the form *xx*. For a given (di-)graph $G=(V,E)$ and a subset $W\subseteq V$, we write *G*[*W*] for the *induced subgraph* of *G* that has vertex set *W* and contains all edges *xy* of *G* for which $x,y\in W$. A digraph $G=(V,E)$ is *connected* if for any pairs of vertices $x,y\in V$ there is a path $x=v_1-v_2-\dots -v_k=y$ such that (i) $v_iv_{i+1} \in E$ or (ii) $v_{i+1}v_i \in E$, $1\le i<k$. The graph *G*(*V*, *E*) is strongly connected if for all $x,y\in V$ there is a sequence $P_{xy}$ that always satisfies Condition (i). For a vertex *x* in a digraph *G* we write $N(x)=\{z \mid xz \in E(G)\}$ and $N^-(x)=\{z \mid zx \in E(G)\}$ for the out- and in-neighborhoods of *x*, respectively. For any set of vertices $A\subseteq L$ we write $N(A):=\bigcup _{x\in A} N(x)$ and $N^-(A):=\bigcup _{x\in A} N^-(x)$.

### Basic properties of best match relations

The best match relation $\mathrel {\rightarrow }$ is reflexive because ${{\,\mathrm{lca}\,}}(x,x)=x\prec {{\,\mathrm{lca}\,}}(x,y)$ for all genes *y* with $\sigma (x)=\sigma (y)$. For any pair of distinct genes *x* and *y* with $\sigma (x)=\sigma (y)$ we have ${{\,\mathrm{lca}\,}}(x,y)\notin \{x,y\}$, hence the relation $\mathrel {\rightarrow }$ has off-diagonal pairs only between genes from different species. There is still a 1-1 correspondence between cBMGs (Definition 2) and best match relations (Definition 1): In the cBMG the reflexive loops are omitted, in the relation $\mathrel {\rightarrow }$ they are added.

The tree $(G,\sigma )$ and the corresponding cBGM $G(T,\sigma )$ employ the same coloring map $\sigma : L\rightarrow S$, i.e., our notion of isomorphy requires the preservation of colors. The usual definition of isomorphisms of colored graphs also allows an arbitrary bijection between the color sets. This is not relevant for our discussion: if $(G',\sigma ')$ and $G(T,\sigma )$ are isomorphic in the usual sense then there is—by definition—a bijective relabeling of the colors in $(G',\sigma ')$ that makes them coincide with the vertex coloring of $G(T,\sigma )$. In other words, if $\varphi$ is an isomorphism from $(G',\sigma ')$ to $G(T,\sigma )$ we assume w.l.o.g. that $\sigma '(x) = \sigma (\varphi (x))$, i.e., each vertex $x\in V(G')$ has the same color as the vertex $\varphi (x)\in V(G)$.

### Thinness

In undirected graphs, equivalence classes of vertices that share the same neighborhood are considered in the context of thinness of the graph (McKenzie 1971; Sumner 1973; Bull and Pease 1989). The concept naturally extends to digraphs (Hellmuth and Marc 2015). For our purposes the following variation on the theme is most useful:

#### Definition 3

Two vertices $x,y\in L$ are in relation  if $N(x)=N(y)$ and $N^-(x)=N^-(y)$.

For each  class $\alpha$ we have $N(x)=N(\alpha )$ and $N^-(x)=N^-(\alpha )$ for all $x\in \alpha$. It is obvious, therefore, that  is an equivalence relation on the vertex set of *G*. Moreover, since we consider loop-free graphs, one can easily see that $G[\alpha ]$ is always edge-less. We write ${\mathscr {N}}$ for the corresponding partition, i.e., the set of  classes of *G*. Individual  classes will be denoted by lowercase Greek letters. Moreover, we write $N_s(x)=\{z\mid z\in N(x) \text { and } \sigma (z)=s\}$ and $N^-_s(x)=\{z\mid z\in N^-(x) \text { and } \sigma (z)=s\}$ for the in- and out-neighborhoods of *x* restricted to a color $s\in S$. For the graphs considered here, we always have $N_{\sigma (x)}(x) = N^-_{\sigma (x)}(x) = \emptyset$. When considering sets $N_s(x)$ and $N^-_s(x)$ we always assume that $s\ne \sigma (x)$. Furthermore, ${\mathscr {N}}_s$ denotes the set of  classes with color *s*.

By construction, the function $$N: V(G)\rightarrow $$$${\mathscr {P}}(V(G))$$, where $${\mathscr {P}}(V(G))$$ is the power set of *V*(*G*), is isotonic, i.e., $$A\subseteq B$$ implies $$N(A)\subseteq N(B)$$. In particular, therefore, we have for $$\alpha ,\beta \in {\mathscr {N}}$$:

(i) $$\alpha \subseteq N(\beta )$$ implies $$N(\alpha ) \subseteq N(N(\beta ))$$
(ii) $$N(\alpha )\subseteq N(\beta )$$ implies $$N(N(\alpha ))\subseteq N(N(\beta ))$$.

These observations will be useful in the proofs below.

By construction every vertex in a cBMG has at least one out-neighbor of every color except its own, i.e., $|N(x)| \ge |S|-1$ holds for all *x*. In contrast, $N^-(x)=\emptyset$ is possible.

### Some simple observations

The color classes *L*[*s*] on the leaves of *T* are independent sets in $G(T,\sigma )$ since arcs in $G(T,\sigma )$ connect only vertices with different colors. For any pair of colors $s,t\in S$, therefore, the induced subgraph $G[L[s]\cup L[t]]$ of $G(T,\sigma )$ is bipartite. Since the definition of $x \mathrel {\rightarrow }y$ does not depend on the presence or absence of vertices *u* with $\sigma (u)\notin \{\sigma (x),\sigma (y)\}$, we have

#### Observation 1

Let $(G,\sigma )$ be a cBMG explained by *T* and let $L' := \bigcup _{s\in S'} L[s]$ be the subset of vertices with a restricted color set $S'\subseteq S$. Then the induced subgraph $(G[L'],\sigma )$ is explained by the restriction $T_{L'}$ of *T* to the leaf set $L'$.

It follows in particular that $G[L[s]\cup L[t]]$ is explained by the restriction $T_{L[s]\cup L[t]}$ of *T* to the colors *s* and *t*. Furthermore, *G* is the edge-disjoint union of bipartite subgraphs corresponding to color pairs, i.e.,

$$\begin{aligned} E(G) = \dot{\bigcup }_{\{s,t\}\in \left( {\begin{array}{c}S\\ 2\end{array}}\right) } E(G_{s,t}). \end{aligned}$$

In order to understand when arbitrary graphs $(G,\sigma )$ are cBMGs, it is sufficient, therefore, to characterize 2-cBMGs. A formal proof will be given later on in Sect. 4.

**Fig. 3:**
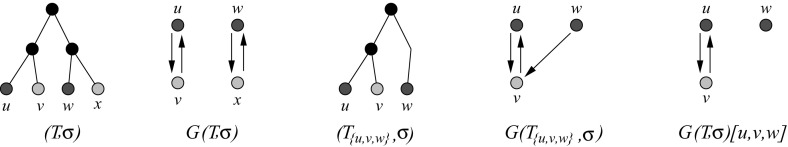
$T_{\{u,v,w\}}$ is displayed by *T* but $G(T_{\{u,v,w\}},\sigma )$ is not isomorphic to the induced subgraph $G(T,\sigma )[\{u,v,w\}]$ of $G(T,\sigma )$, since $G(T_{\{u,v,w\}},\sigma )$ contains the additional arc $w\rightarrow v$

Note the condition that “*T* explains $(G,\sigma )$” does not imply that $(T_{L'},\sigma )$ explains $(G[L'],\sigma )$ for arbitrary subsets of $L'\subseteq L$. Figure 3 shows that, indeed, not every induced subgraph of a cBMG is necessarily a cBMG. However, we have the following, weaker property:

#### Lemma 1

Let $(G,\sigma )$ be the cBMG explained by $(T,\sigma )$, let $T'=T_{L'}$ and let $(G',\sigma )$ be the cBMG explained by $(T',\sigma )$. Then $u,v \in L'$ and $uv\in E(G)$ implies $uv\in E(G')$. In other words, $(G[L'],\sigma )$ is always a subgraph of $(G'[L'],\sigma )$.

#### Proof

If $uv\in E(G)$ then ${{\,\mathrm{lca}\,}}_T(u,v) \preceq _T {{\,\mathrm{lca}\,}}_T(u,z)$ for all $z\in L[\sigma (v)]$, and thus the inequality ${{\,\mathrm{lca}\,}}_{T'}(u,v)\preceq _{T'} {{\,\mathrm{lca}\,}}_{T'}(u,z)$ is true in particular for all $z\in L'\cap L[\sigma (v)]=L'[\sigma (v)]$. $\square$

### Connectedness

We briefly present some results concerning the connectedness of cBMGs. In particular, it turns out that connected cBMGs have a simple characterization in terms of their representing trees.

#### Theorem 1

Let $(T,\sigma )$ be a leaf-labeled tree and $G(T,\sigma )$ its cBMG. Then $G(T,\sigma )$ is connected if and only if there is a child *v* of the root $\rho$ such that $\sigma (L(T(v)))\ne S$. Furthermore, if $G(T,\sigma )$ is not connected, then for every connected component *C* of $G(T,\sigma )$ there is a child *v* of the root $\rho$ such that $V(C)\subseteq L(T(v))$.

#### Proof

For convenience we write $L_v:=L(T(v))$. Suppose $\sigma (L_v)=S$ holds for all children *v* of the root. Then for any pair of colors $s,t\in S$ we find for a leaf $x\in L_v$ with $\sigma (x)=s$ a leaf $y\in L_v$ with $\sigma (y)=t$ within *T*(*v*); thus ${{\,\mathrm{lca}\,}}(x,y)$ is in *T*(*v*) and thus ${{\,\mathrm{lca}\,}}(x,y)\prec \rho$. Hence, all best matching pairs are confined to the subtrees below the children of the root. The corresponding leaf sets are thus mutually disconnected in $G(T,\sigma )$.

Conversely, suppose that one of the children *v* of the root $\rho$ satisfies $\sigma (L_v)\ne S$. Therefore, there is a color $t\in S$ with $t\notin \sigma (L_v)$. Then for every $x\in L_v$ there is an arc $x\mathrel {\rightarrow }z$ for all $z\in L[t]$ since for all such *z* we have ${{\,\mathrm{lca}\,}}(x,z)=\rho$. If $L[t] = L{\setminus } L_v$, we can conclude that $G(T,\sigma )$ is a connected digraph. Otherwise, every leaf $y\in L{\setminus } L_v$ with a color $\sigma (y)\ne t$ has an out-arc $y\mathrel {\rightarrow }z$ to some $z\in L[t]$ and thus there is a path $y\rightarrow z \leftarrow x$ connecting $y\in L{\setminus } L_v$ to every $x\in L_v$. Finally, for any two vertices $y,y'\in L{\setminus } (L_v\cup L[t])$ there are vertices $z,z'\in L[t]$ such that arcs exist that form a path $y\rightarrow z \leftarrow x \rightarrow z' \leftarrow y'$ connecting *z* with $z'$ and both to any $x\in L_v$. In summary, therefore, $G(T,\sigma )$ is a connected digraph.

For the last statement, we argue as above and conclude that if $\sigma (L_v) = S$ for all children *v* of the root (or, equivalently, if $G(T,\sigma )$ is not connected), then all best matching pairs are confined to the subtrees below the children of the root $\rho$. Thus, the vertices of every connected component of $G(T,\sigma )$ must be leaves of a subtree *T*(*v*) for some child *v* of the root $\rho$. $\square$

The following result shows that cBMGs can be characterized by their connected components: the disjoint union of vertex disjoint cBMGs is again a cBMG if and only if they all share the same color set. It suffices therefore, to consider each connected component separately.

#### Proposition 1

Let $(G_i,\sigma _i)$ be vertex disjoint cBMGs with vertex sets $L_i$ and color sets $S_i=\sigma _i(L_i)$ for $1\le i \le k$. Then the disjoint union $(G,\sigma ):=\dot{\bigcup }_{i=1}^k(G_i,\sigma _i)$ is a cBMG if and only if all color sets are the same, i.e., $\sigma _i(L_i)=\sigma _j(L_j)$ for $1\le i,j\le k$.

#### Proof

The statement is trivially fulfilled for $k=1$. For $k\ge 2$, the disjoint union $(G,\sigma )$ is not connected. Assume that $\sigma _i(L_i)=\sigma _j(L_j)$ for all *i*, *j*. Let $(T_i,\sigma _i)$ be trees explaining $(G_i,\sigma _i)$ for $1\le i \le k$. We construct a tree $(T,\sigma )$ as follows: Let $\rho$ be the root of $(T,\sigma )$ with children $r_1, \dots r_k$. Then we identify $r_i$ with the root of $T_i$ and retain all leaf colors. In order to show that $(T,\sigma )$ explains $(G,\sigma )$ we recall from Theorem 1 that all best matching pairs are confined to the subtrees below the children of the root and hence, each connected component of $(G,\sigma )$ forms a subset of one of the leaf sets $L_i$. Since each $(T_i,\sigma _i)$ explains $(G_i,\sigma _i)$, we conclude that the cBMG explained by $(T,\sigma )$ is indeed the disjoint union of the $(G_i,\sigma _i)$, i.e., $(G,\sigma )$. Thus $(G,\sigma )$ is a cBMG.

Conversely, assume that $(G,\sigma )$ is a cBMG but $\sigma _i(L_i)\ne {\sigma _k(L_k)}$ for some ${k}\ne i$. By construction, $\sigma (L_i) = \sigma _i(L_i)$ and $\sigma ({L_k})={\sigma _k(L_k)}$. In particular, for every color $t\notin \sigma (L_i)$ and every vertex $x\in L_i$, there is a $j\ne i$ with $t\in \sigma (L_j)$ such that there exists an outgoing arc form *x* to some vertex $y\in L_j$ with color $\sigma (y)=t$. Thus (*x*, *y*) is an arc connecting $L_i$ with some $L_j$, $j\ne i$, contradicting the assumption that each $L_i$ forms a connected component of $(G,\sigma )$. Hence, the color sets cannot differ between connected components. $\square$

The example $(G(T_{\{u,v,w\}}),\sigma )$ in Fig. 3 already shows however that $G(T,\sigma )$ is not necessarily strongly connected.

## Two-colored best match graphs (2-cBMGs)

Through this section we assume that $\sigma (L)=\{s,t\}$ contains exactly two colors.

### Thinness classes

A connected 2-cBMG contains at least two  classes, since all in- and out-neighbors *y* of *x* by construction have a color $\sigma (y)$ different from $\sigma (x)$. Consequently, a 2-cBMG is bipartite. Furthermore, if $\sigma (x)\ne \sigma (y)$ then $N(x)\cap N(y)=\emptyset$. Since $N(x)\ne \emptyset$ and all members of *N*(*x*) have the same color, we observe that $N(x)=N(y)$ implies $\sigma (x)=\sigma (y)$. By a slight abuse of notation we will often write $\sigma (x)=\sigma (\alpha )$ for an element *x* of some  class $\alpha$. Two leaves *x* and *y* of the same color that have the same last common ancestor with all other leaves in *T*, i.e., that satisfy ${{\,\mathrm{lca}\,}}(x,u)={{\,\mathrm{lca}\,}}(y,u)$ for all $u\in L{\setminus }\{x,y\}$ by construction have the same in-neighbor and the same out-neighbors in $G(T,\sigma )$; hence .

#### Observation 2

Let $(G,\sigma )$ be a connected 2-cBMG and $\alpha \in {\mathscr {N}}$ be a  class. Then, $\sigma (x) =\sigma (y)$ for any $x,y\in \alpha$.

The following result shows that the out-neighborhood of any  class is a disjoint union of  classes.

**Fig. 4:**
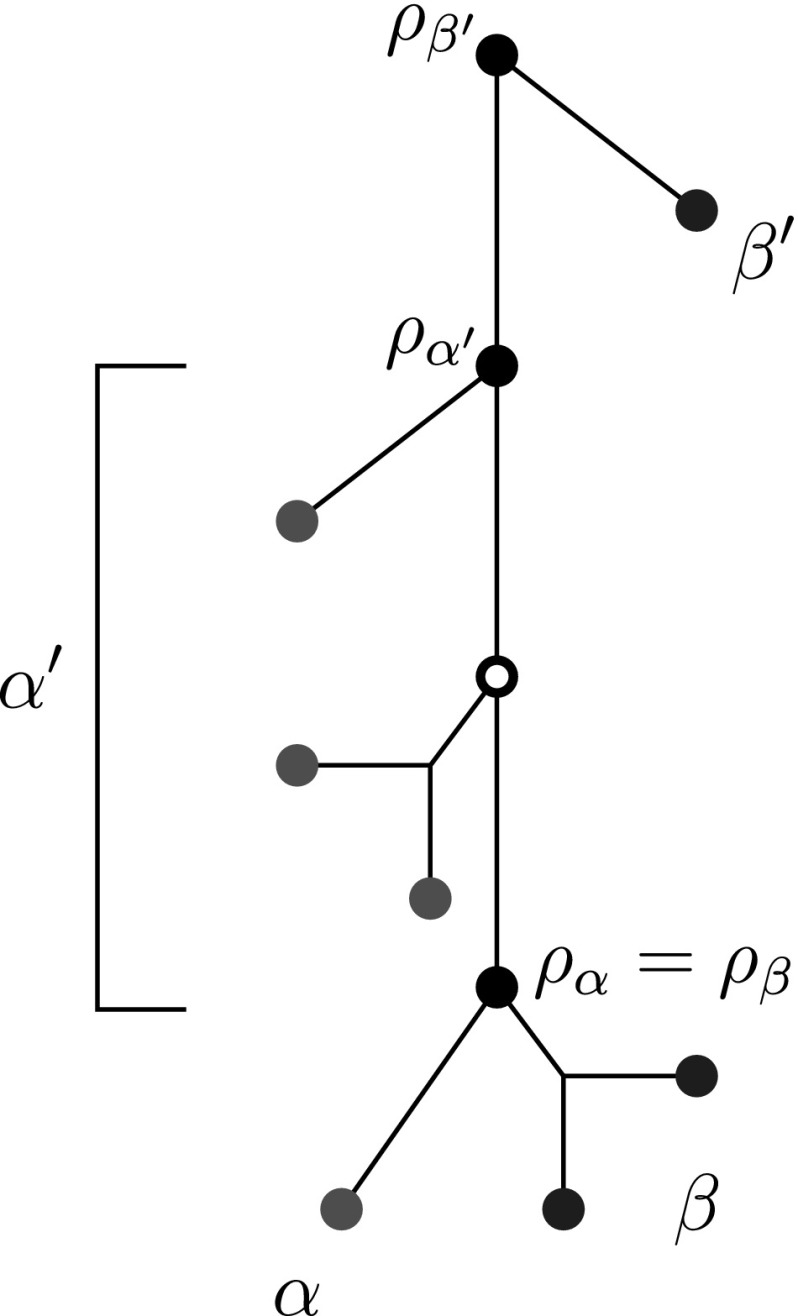
Relationship between  classes and their roots. A tree with two colors (red and blue) and four  classes $\alpha$, $\alpha '$ (red) and $\beta$, $\beta '$ (blue) together with their corresponding roots $\rho _\alpha$, $\rho _{\alpha '}$, $\rho _\beta$ and $\rho _{\beta '}$ are shown (color figure online)

#### Lemma 2

Let $(G,\sigma )$ be a connected 2-cBMG. Then any two  classes $\alpha ,\beta \in {\mathscr {N}}$ satisfy

(N0) $$\beta \subseteq N(\alpha )$$ or $$\beta \cap N(\alpha )=\emptyset $$.

#### Proof

For any $y\in \beta$, the definition of  classes implies that $y\in N(\alpha )$ if and only if $\beta \subseteq N(\alpha )$. Hence, either all or none of the elements of $\beta$ are contained in $N(\alpha )$. $\square$

The connection between the  classes of $G(T,\sigma )$ and the tree $(T,\sigma )$ is captured by identifying an internal node in *T* that is, as we shall see, in a certain sense characteristic for a given equivalence class (Fig. 4).

#### Definition 4

The *root*$\rho _{\alpha }$*of the**class*$\alpha$ is

$$\begin{aligned} \rho _\alpha =\max _{\begin{array}{c} x\in \alpha \\ y\in N(\alpha ) \end{array}} {{\,\mathrm{lca}\,}}(x,y). \end{aligned}$$

#### Corollary 1

Let $\rho _\alpha$ be the root of a  class $\alpha$. Then, for any $y\in N(\alpha )$ holds

$$\begin{aligned} \rho _\alpha =\max _{x\in \alpha } {{\,\mathrm{lca}\,}}(x,y). \end{aligned}$$

In particular, ${{\,\mathrm{lca}\,}}(x,y) = {{\,\mathrm{lca}\,}}(x,z)$ for all $y,z\in N(\alpha )$.

#### Proof

For any $y\in N(\alpha )$ it holds by definition of $N(\alpha )$ that ${{\,\mathrm{lca}\,}}(x,y)\preceq {{\,\mathrm{lca}\,}}(x,z)$ for $x\in \alpha$ and any *z* with $\sigma (z)=\sigma (y)$. This together with Observation 2 implies that ${{\,\mathrm{lca}\,}}(x,y)={{\,\mathrm{lca}\,}}(x,z)$ for any two $y,z\in N(\alpha )$ and $x\in \alpha$. $\square$

The following lemma collects some simple properties of the roots of  classes that will be useful for the proofs of the main results.

#### Lemma 3

Let $(G,\sigma )$ be a connected 2-cBMG explained by $(T,\sigma )$ and let $\alpha$, $\beta$ be  classes with roots $\rho _{\alpha }$ and $\rho _{\beta }$, respectively. Then the following statements hold

(i) $\rho _{\alpha }\preceq {{\,\mathrm{lca}\,}}(\alpha ,\beta )$ and $\rho _\beta \preceq {{\,\mathrm{lca}\,}}(\alpha ,\beta )$; equality holds for at least one of them if and only if $\rho _\alpha , \rho _\beta$ are comparable, i.e., $\rho _\alpha \preceq \rho _\beta$ or $\rho _\beta \preceq \rho _\alpha$.
(ii) The subtree $T(\rho _{\alpha })$ contains leaves of both colors.
(iii) $$N(\alpha )\preceq \rho _{\alpha }$$.
(iv) If $\beta \subseteq N(\alpha )$ then $\rho _{\beta }\preceq \rho _{\alpha }$.
(v) If $\rho _\alpha =\rho _\beta$ and $\alpha \ne \beta$, then $\sigma (\alpha )\ne \sigma (\beta )$.
(vi) $$N(\alpha )=\{y \mid y\in L(T(\rho _\alpha )) \text { and } \sigma (y)\ne \sigma (\alpha )\}$$
(vii) $$N(N(\alpha ))\preceq \rho _\alpha $$.

#### Proof

(i) By Condition (N0) in Lemma 2 we have either $\beta \subseteq N(\alpha )$ or $\beta \cap N(\alpha )=\emptyset$. By definition of $N(\beta )$, we have ${{\,\mathrm{lca}\,}}(x',y)\preceq {{\,\mathrm{lca}\,}}(x,y)$ where $y\in \beta$, $x'\in N(\beta )$, and $x\in \alpha$. Therefore, if $\beta \subseteq N(\alpha )$, then $\rho _\beta =\max _{x'\in N(\beta )}{{\,\mathrm{lca}\,}}(x',\beta )\preceq \max _{x\in \alpha }{{\,\mathrm{lca}\,}}(x,\beta )={{\,\mathrm{lca}\,}}(\alpha ,\beta )$. Moreover, Corollary 1 implies $\rho _\alpha =\max _{y\in N(\alpha )} {{\,\mathrm{lca}\,}}(\alpha ,y)= \max _{y \in \beta }{{\,\mathrm{lca}\,}}(\alpha ,y)= {{\,\mathrm{lca}\,}}(\alpha ,\beta )$.

If $\beta \cap N(\alpha )=\emptyset$, then ${{\,\mathrm{lca}\,}}(\alpha ,y)\succ \max _{y'\in N(\alpha )}{{\,\mathrm{lca}\,}}(\alpha ,y')=\rho _\alpha$ for all $y\in \beta$, i.e., ${{\,\mathrm{lca}\,}}(\alpha ,\beta )\succ \rho _\alpha$. Moreover, by definition of $\rho _\beta$, we have $\rho _\beta =\max _{x\in N(\beta )}{{\,\mathrm{lca}\,}}(x,\beta )\preceq \max _{x\in \alpha }{{\,\mathrm{lca}\,}}(x,\beta )={{\,\mathrm{lca}\,}}(\alpha ,\beta )$.

Now assume that $\rho _\alpha$ and $\rho _\beta$ are comparable. W.l.o.g. we assume $\rho _\alpha \succeq \rho _\beta$. Since $\alpha \preceq \rho _\alpha$ and $\beta \preceq \rho _\beta$ is true by definition, we obtain ${{\,\mathrm{lca}\,}}(\alpha ,\beta )=\rho _\alpha \succeq \rho _\beta$. Conversely, if $\rho _\alpha ={{\,\mathrm{lca}\,}}(\alpha ,\beta )\succeq \rho _\beta$, then $\rho _\alpha$ and $\rho _\beta$ are necessarily comparable.

(ii) As argued above, $N(x)\ne \emptyset$ for all vertices *x*. Let $x\in \alpha$ and $y\in N(x)$ such that $\rho _\alpha = {{\,\mathrm{lca}\,}}(x,y)$. By definition, $\sigma (x)\ne \sigma (y)$. Since $\rho _\alpha$ is an ancestor of both *x* and *y*, the statement follows.

(iii) Since $T(\rho _{\alpha })$ contains leaves of both colors, there is in particular a leaf *y* with $\sigma (y)\ne \sigma (x)$ within $T(\rho _{\alpha })$. It satisfies ${{\,\mathrm{lca}\,}}(x,y)\preceq \rho _{\alpha }$ and thus all arcs going out from $x\in \alpha$ are confined to leaves of $T(\rho _{\alpha })$, i.e., $N(\alpha )\preceq \rho _{\alpha }$.

(iv) is a direct consequence of (i) and (iii).

(v) Assume for contradiction that $\sigma (\alpha )=\sigma (\beta )$. There is some $y\in N(\alpha )$ with ${{\,\mathrm{lca}\,}}(\alpha ,y)=\rho _\alpha$. Since $\rho _\alpha =\rho _\beta ={{\,\mathrm{lca}\,}}(\alpha ,\beta )$ by (i), we have ${{\,\mathrm{lca}\,}}(\alpha ,y)\succeq {{\,\mathrm{lca}\,}}(\beta ,y)$. By definition of $\rho _\beta$, there is a $z\in N(\beta )$ such that ${{\,\mathrm{lca}\,}}(\beta ,z)=\rho _\beta$. Thus, ${{\,\mathrm{lca}\,}}(\beta ,y)\preceq {{\,\mathrm{lca}\,}}(\beta ,z)$, which implies that *y* is a best match of $\beta$, i.e., $y\in N(\beta )$. Hence, $N(\alpha )=N(\beta )$. On the other hand, since ${{\,\mathrm{lca}\,}}(\alpha ,\beta )=\rho _\alpha$, we have ${{\,\mathrm{lca}\,}}(\alpha ,y)={{\,\mathrm{lca}\,}}(\beta ,y)$ for any *y* with ${{\,\mathrm{lca}\,}}(\alpha ,y)\succeq \rho _\alpha$. As a consequence, since $\rho _\alpha \preceq {{\,\mathrm{lca}\,}}(\alpha ,y')$ for all $y'\in N^-(\alpha )$, it is true that ${{\,\mathrm{lca}\,}}(y',\beta )={{\,\mathrm{lca}\,}}(y',\alpha )\preceq {{\,\mathrm{lca}\,}}(y',z)$, for all *z* with $\sigma (z)=\sigma (\alpha )$. Hence $y\in N^-(\alpha )$ if and only if $y\in N^-(\beta )$. It follows that $\alpha =\beta$, a contradiction.

(vi) Let $y\in N(\alpha )$, then $\sigma (y)\ne \sigma (\alpha )$ by definition. In addition, we have $y\preceq \rho _\alpha$ by (iii). Conversely, suppose that $y\in L(T(\rho _\alpha ))$ and $\sigma (y)\ne \sigma (\alpha )$. Since $y\in L(T(\rho _\alpha ))$, it is true that $y,\alpha \preceq \rho _\alpha$ and therefore, ${{\,\mathrm{lca}\,}}(\alpha ,y)\preceq \rho _\alpha$. By definition of the root of $\alpha$, there exist $x'\in \alpha$ and $y'\in N(\alpha )$ such that $\rho _\alpha ={{\,\mathrm{lca}\,}}(x',y')\preceq {{\,\mathrm{lca}\,}}(x',z)$ for all *z* with $\sigma (z)=\sigma (y')$. Since ${{\,\mathrm{lca}\,}}(\alpha ,y)\preceq \rho _\alpha$, this implies $y\in N(\alpha )$.

(vii) Lemma 2 and (iv) imply that $N(\alpha )$ is a disjoint union of  classes $\gamma$ with $\rho _\gamma \preceq \rho _\alpha$ and $\sigma (\gamma )\ne \sigma (\alpha )$. Thus, $$N(N(\alpha ))= \bigcup _{\begin{array}{c} \gamma \in {\mathscr {N}}\\ \gamma \subseteq N(\alpha ) \end{array}} N(\gamma )= N(\bigcup _{\begin{array}{c} \gamma \in {\mathscr {N}}\\ \gamma \subseteq N(\alpha ) \end{array}} \gamma )$$. By (iii) and (iv), we have $N(\gamma )\preceq \rho _\alpha$ for any such $\gamma$, thus $N(N(\alpha ))\preceq \rho _\alpha$. $\square$

(N0) implies that there are four distinct ways in which two  classes $\alpha$ and $\beta$ with distinct colors can be related to each other. These cases distinguish the relative location of their roots $\rho _\alpha$ and $\rho _\beta$:

#### Lemma 4

If $(G,\sigma )$ is a connected 2-cBMG, and $\alpha$, $\beta$ are  classes with $\sigma (\alpha )\ne \sigma (\beta )$. Then exactly one of the following four cases is true

(i) $\alpha \subseteq N(\beta )$ and $\beta \subseteq N(\alpha )$. In this case $\rho _{\alpha }=\rho _{\beta }$.
(ii) $\alpha \subseteq N(\beta )$ and $\beta \cap N(\alpha )=\emptyset$. In this case $\rho _{\alpha }\prec \rho _{\beta }$.
(iii) $\beta \subseteq N(\alpha )$ and $\alpha \cap N(\beta )=\emptyset$. In this case $\rho _{\beta }\prec \rho _{\alpha }$.
(iv) $\alpha \cap N(\beta )=\beta \cap N(\alpha )=\emptyset$. In this case $\rho _{\alpha }$ and $\rho _{\beta }$ are not $\preceq$-comparable.

#### Proof

Set $\sigma (\alpha )=s$ and $\sigma (\beta )=t$, $s\ne t$, and consider the roots $\rho _\alpha$ and $\rho _\beta$ of the two  classes. Then, there are exactly four cases:

(i) For $\rho _\alpha =\rho _\beta$, Lemma 3(i) implies $\rho _\alpha =\rho _\beta ={{\,\mathrm{lca}\,}}(\alpha ,\beta )$. By definition of $\rho _\alpha$, $y\in N(\alpha )$ for all $y\in L(T(\rho _\alpha ))$ with $\sigma (y)\ne \sigma (\alpha )$ by Lemma 3(vi). A similar result holds for $\rho _\beta$. It follows immediately that $\alpha \subseteq N(\beta )$ and $\beta \subseteq N(\alpha )$.

(ii) In the case $\rho _\alpha \succ \rho _\beta$, Lemma 3(i) implies $\rho _\alpha ={{\,\mathrm{lca}\,}}(\alpha ,\beta )$ and thus, similarly to case (i), $\beta \subseteq N(\alpha )$. On the other hand, by Lemma 3(ii) and $\rho _\alpha \succ \rho _\beta$, there is a leaf $x'\in L(T(\rho _\beta )){\setminus } \alpha$ with $\sigma (x')=s$. Hence, ${{\,\mathrm{lca}\,}}(x',\beta )\prec \rho _\alpha ={{\,\mathrm{lca}\,}}(\alpha ,\beta )$, which implies $\alpha \cap N(\beta )=\emptyset$.

(iii) The case $\rho _\alpha \prec \rho _\beta$ is symmetric to (ii).

(iv) If $\rho _\alpha ,\rho _\beta$ are incomparable, it yields $\rho _\alpha , \rho _\beta \ne \rho$ and ${{\,\mathrm{lca}\,}}(\alpha ,\beta )=\rho$, where $\rho$ denotes the root of *T*. Since $\beta \preceq \rho _\beta$, Lemma 2 implies $\beta \cap N(\alpha )=\emptyset$. Similarly, $\alpha \cap N(\beta )=\emptyset$. $\square$

### Least resolved trees

In general, there are many trees that explain the same 2-cBMG. We next show that there is a unique “smallest” tree among them, which we will call the least resolved tree for $(G,\sigma )$. Later-on, we will derive a hierarchy of leaf sets from $(G,\sigma )$ whose tree representation coincides with this least resolved tree. We start by introducing a systematic way of simplifying trees. Let *e* be an interior edge of $(T,\sigma )$. Then the tree $T_e$ obtained by contracting the edge $e=uv$ is derived by identifying *u* and *v*. Analogously, we write $T_A$ for the tree obtained by contracting all edges in *A*.

#### Definition 5

Let $(G,\sigma )$ be a cBMG and let $(T,\sigma )$ be a tree explaining $(G,\sigma )$. An interior edge *e* in $(T,\sigma )$ is *redundant* if $(T_e,\sigma )$ also explains $(G,\sigma )$. Edges that are not redundant are called *relevant*.

The next two results characterize redundant edges and show that such edges can be contracted in an arbitrary order.

#### Lemma 5

Let $(T,\sigma )$ be a tree that explains a connected 2-cBMG $(G,\sigma )$. Then, the edge $e=uv$ is redundant if and only if *e* is an inner edge and there exists no  class $\alpha$ such that $v=\rho _\alpha$.

#### Proof

First we note that $e=uv$ must be an inner edge. Otherwise, i.e., if *e* is an outer edge, then $v\notin L(T_e)$ and thus, $(T_e,\sigma )$ does not explain $(G,\sigma )$. Now suppose that *e* is an inner edge, which in particular implies $L(T_e)=L(T)$, and that *e* is redundant. Assume for contradiction that there is a  class $\alpha$ such that $v=\rho _\alpha$. Since $(T,\sigma )$ is phylogenetic, $T(u){\setminus } T(v)$ has to be non-empty. If there is a leaf $y\in L(T(u){\setminus } T(v))$ with $\sigma (y)\ne \sigma (\alpha )$ in $(T,\sigma )$, then $y\notin N(\alpha )$ by Lemma 3(vi). But then, contraction of *e* implies $y\in T(\rho _\alpha )$ and therefore $y\in N(\alpha )$, thus $(T_e,\sigma )$ does not explain $(G,\sigma )$. Consequently, $T(u){\setminus } T(v)$ can only contain leaves *x* with $\sigma (x)=\sigma (\alpha )$. Indeed, for any $y' \in T(v)$ it is true that $v=\rho _\alpha ={{\,\mathrm{lca}\,}}(\alpha ,y')\prec {{\,\mathrm{lca}\,}}(x,y')$, i.e., $N^-(x)\ne N^-(\alpha )$ and thus $x\notin \alpha$. By contracting *e*, we obtain ${{\,\mathrm{lca}\,}}(x,z)\succeq uv=\rho _\alpha$ which implies $N(x)=N(\alpha )$ and $N^-(x)=N^-(\alpha )$, and therefore $x\in \alpha$. Hence, $(T_e,\sigma )$ does not explain $(G,\sigma )$.

Conversely, assume that *e* is an inner edge and there is no  class $\alpha$ such that $v=\rho _{\alpha }$, i.e., for each $\alpha \in {\mathscr {N}}$ it either holds (i) $v\prec \rho _\alpha$, (ii) $v\succ \rho _\alpha$, or (iii) *v* and $\rho _\alpha$ are incomparable. In the first and second case, contraction of *e* implies either $v\preceq \rho _\alpha$ or $v\succeq \rho _\alpha$. Thus, since $L(T(w))=L(T_e(w))$ is clearly satisfied if *w* and *v* are incomparable, we have $L(T(w))=L(T_e(w))$ for every $w\ne v$. Moreover, $N(\alpha )=\{y \mid y\in L(T(\rho _\alpha )),\sigma (y)\ne \sigma (\alpha )\}$ by Lemma 3(vi). Together these facts imply for every  class $\alpha$ with $\rho _\alpha \ne v$ that $N(\alpha )$ remains unchanged in $(T_e,\sigma )$ after contraction of *e*. Since the out-neighborhoods of all  classes are unaffected by contraction of *e*, all in-neighborhoods also remain the same in $(T_e,\sigma )$. Therefore, $(T,\sigma )$ and $(T_e,\sigma )$ explain the same graph $(G,\sigma )$. $\square$

#### Lemma 6

Let $(T,\sigma )$ be a tree that explains a connected 2-cBMG $(G,\sigma )$ and let *e* be a redundant edge. Then the edge $f\ne e$ is redundant in $(T_e,\sigma )$ if and only if *f* is redundant in $(T,\sigma )$. Moreover, if two edges $e\ne f$ are redundant in $(T,\sigma )$, then $((T_e)_f,\sigma )$ also explains $(G,\sigma )$.

#### Proof

Let $e=uv$ be a redundant edge in $(T,\sigma )$. Then, for any vertex $w\ne u,v$ in $(T,\sigma )$ it is true that *w* is the root of a  class $\alpha$ in $(T_e,\sigma )$ if and only if *w* is the root of $\alpha$ in $(T,\sigma )$. In particular, the vertex *uv* in $(T_e,\sigma )$ is the root of a  class $\alpha '$ if and only if $u=\rho _{\alpha '}$ in $(T,\sigma )$. Consequently, *f* is redundant in $(T,\sigma )$ if and only if *f* is redundant in $(T_e,\sigma )$. $\square$

As an immediate consequence, contraction of edges is commutative, i.e., the order of the contractions is irrelevant. We can therefore write $T_A$ for the tree obtained by contracting all edges in *A* in arbitrary order:

#### Corollary 2

Let $(T,\sigma )$ be a tree that explains a 2-cBMG $(G,\sigma )$ and let *A* be a set of redundant edges of $(T,\sigma )$. Then, $(T_A,\sigma )$ explains $(G,\sigma )$. In particular, $((T_A)_B,\sigma )$ explains $(G,\sigma )$ if and only if *B* is a set of redundant edges of $(T,\sigma )$.

#### Definition 6

Let $(G,\sigma )$ be a cBMG explained by $(T,\sigma )$. We say that $(T,\sigma )$ is *least resolved* if $(T_A,\sigma )$ does not explain $(G,\sigma )$ for any non-empty set *A* of interior edges of $(T,\sigma )$.

We are now in the position to formulate the main result of this section:

#### Theorem 2

For any connected 2-cBMG $(G,\sigma )$, there exists a unique least resolved tree $(T',\sigma )$ that explains $(G,\sigma )$. $(T',\sigma )$ is obtained by contraction of all redundant edges in an arbitrary tree $(T,\sigma )$ that explains $(G,\sigma )$. The set of all redundant edges in $(T,\sigma )$ is given byMoreover, $(T',\sigma )$ is displayed by $(T,\sigma )$.

#### Proof

Any edge in a least resolved tree $(T',\sigma )$ is non-redundant and therefore, as a consequence of Corollary 2, $(T',\sigma )$ is obtained from $(T,\sigma )$ by contraction of all redundant edges of $(T,\sigma )$. According to Lemma 5, the set of redundant edges is exactly ${\mathfrak {E}}_T$. Since the order of contracting the edges in ${\mathfrak {E}}_T$ is arbitrary, there is a least resolved tree for every given tree $(T,\sigma )$.

Now assume for contradiction that there exist colored digraphs that are explained by two distinct least resolved trees. Let $(G,\sigma )$ be a minimal graph (w.r.t. the number of vertices) that is explained by two distinct least resolved trees $(T_1,\sigma )$ and $(T_2,\sigma )$ and let $v\in L$ with $\sigma (v)=s$. By construction, the two trees $(T_1',\sigma ')$ and $(T_2',\sigma ')$ with $T_1':=T_1{_{|L{\setminus }\{v\}}}$, $T_2':=T_2{_{|L{\setminus }\{v\}}}$ and leaf labeling $\sigma ':=\sigma _{|L{\setminus } \{v\}}$, each explain a unique graph, which we denote by $(G_1',\sigma ')$ and $(G_2',\sigma ')$, respectively. Lemma 1 implies that $(G',\sigma '):=(G[L{\setminus } \{v\}],\sigma ')$ is a subgraph of both $(G_1',\sigma ')$ and $(G_2',\sigma ')$.

We next show that $(G_1',\sigma ')$ and $(G_2',\sigma ')$ are equal by characterizing the additional edges that are inserted in both graphs compared to $(G',\sigma ')$. Assume that there is an additional edge *uy* in one of the graphs, say $(G_1',\sigma )$. Since *uy* is not an edge in $(G,\sigma )$, we have ${{\,\mathrm{lca}\,}}_T(u,y)\succ _T {{\,\mathrm{lca}\,}}_T(u,y')$ for some $y'\in L(T)$ with $\sigma (y)=\sigma (y')$. However, $uy\in E(G_1')$ implies that ${{\,\mathrm{lca}\,}}_{T_1}(u,y)\preceq _{T_1}{{\,\mathrm{lca}\,}}_{T_1}(u,y'')$ for all $y''\in L{\setminus } \{v\}$ with $\sigma (y)=\sigma (y')$. Since $T_1':=T_1{\setminus } \{v\}$, we obtain ${{\,\mathrm{lca}\,}}_T(u,y') \prec _T {{\,\mathrm{lca}\,}}_T(u,y) \preceq _{T} {{\,\mathrm{lca}\,}}_{T}(u,y'')$, which implies that $y'=v$ and, in particular, $uv\in E(G)$ and $N(u) = \{v\}$.

**Fig. 5:**
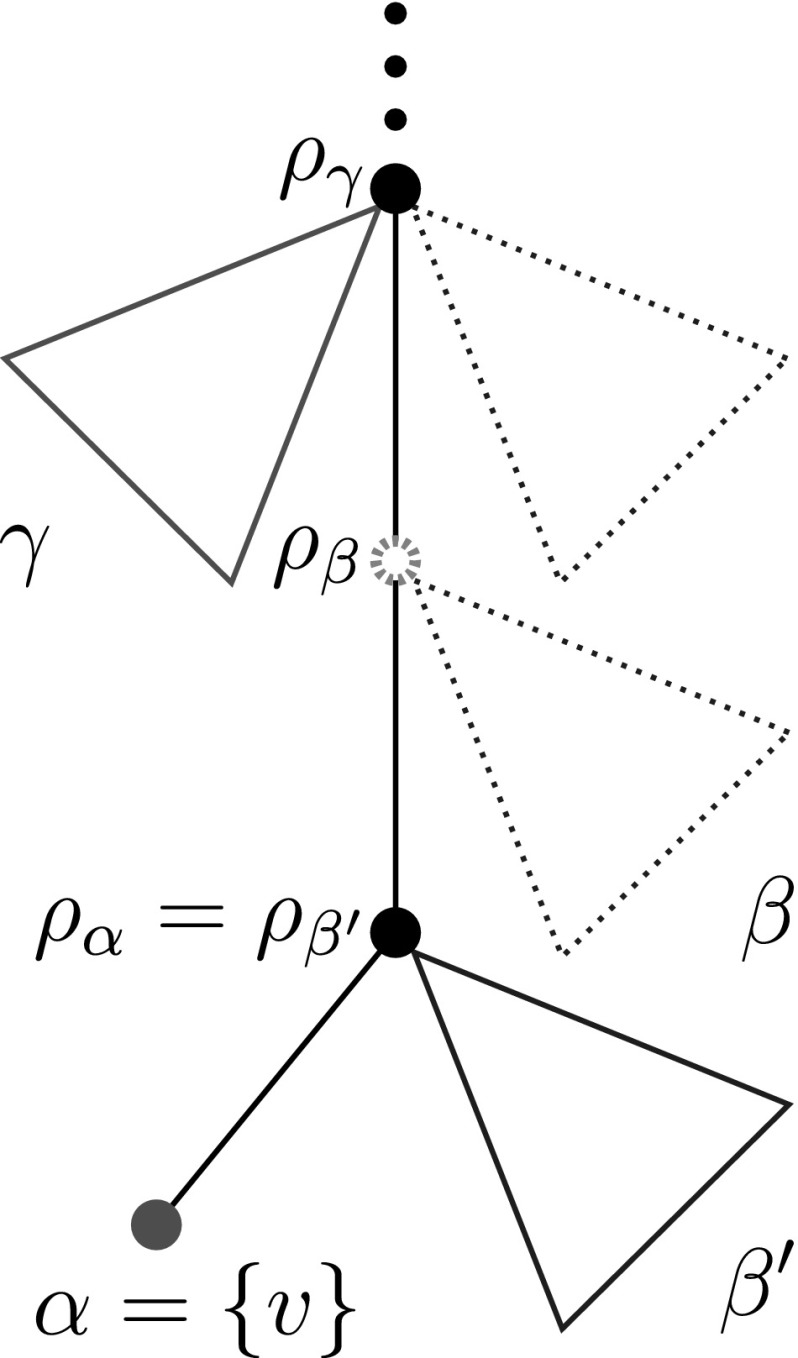
Illustration of the proof of Theorem 2, showing the local subtrees of $(T_1,\sigma )$ and $(T_2,\sigma )$, immediately above $\alpha =\{v\}$. The relevant portion extends to the root $\rho _{\gamma }$ of the  class $\gamma$ that is located immediately above of $\alpha$ and has the same color as $\alpha$, here red. Clearly, the deletion of $\alpha$ can affect only pairs of vertices *x*, *y* with ${{\,\mathrm{lca}\,}}(x,y)$ below $\rho _{\gamma }$. Triangles denote the subtree that consists of all leaves of the corresponding class which are attached to the root of the class by an outer edge. Dashed triangles and nodes denote subtrees which may or may not be present in $(T_1,\sigma )$ and $(T_2,\sigma )$

In particular, we have $\sigma (u)=t\ne s$. In this case, *u* has no out-neighbors in $(G',\sigma ')$ but it has outgoing arcs in $(G_1',\sigma ')$ and $(G_2',\sigma ')$. In order to determine these outgoing arcs explicitly, we will reconstruct the local structure of $(T_1,\sigma )$ and $(T_2,\sigma )$ in the vicinity of the leaf *v*. The following argumentation is illustrated in Fig. 5.

Since $N(u)=\{v\}$, there is a  class $\alpha =\{v\}$. Let $\beta$ be the  class of $(G,\sigma )$ to which *u* belongs. It satisfies $N(\beta )=\{v\}$. Therefore, $L(T_1(\rho _\beta ))\cap L[s]=\{v\}$ and $L(T_2(\rho _\beta ))\cap L[s]=\{v\}$. In particular, this implies $L(T_1(\rho _\alpha ))\cap L[s]=\{v\}$ and $L(T_2(\rho _\alpha ))\cap L[s]=\{v\}$. The children of $\rho _\alpha$ in both $T_1$ and $T_2$ must be leaves: otherwise, Lemma 3(ii) would imply that there are inner vertices $\rho _{\alpha '}$ and $\rho _{\beta '}$ below $\rho _\alpha$, which in turn would contradict to $L(T_1(\rho _\alpha ))\cap L[s]=\{v\}$ and $L(T_2(\rho _\alpha ))\cap L[s]=\{v\}$.

Moreover, the subtrees $T_1(\rho _\alpha )$ and $T_2(\rho _\alpha )$ must contain leaves of both colors. Thus there exists a  class $\beta '$ with color *t* whose root $\rho _{\beta '}$ coincides with $\rho _\alpha$ in both $(T_1,\sigma )$ and $(T_2,\sigma )$. More precisely, we have $\mathsf {child}(\rho _\alpha )=\alpha \cup \beta '$. We now distinguish two cases:

(i)   If $N^-(\beta )\cap \{v\}\ne \emptyset$ in $(G,\sigma )$, we have $\rho _\beta =\rho _\alpha$, i.e., $\beta =\beta '$.

(ii)   Otherwise if $N^-(\beta )\cap \{v\}= \emptyset$, then ${{\,\mathrm{lca}\,}}(v,\beta ')\prec {{\,\mathrm{lca}\,}}(v,\beta )$, hence $\rho _\beta \succ \rho _\alpha$. In particular, since $N(\beta )=\{v\}$, Lemma 3(vi) implies that there cannot be any other  class $\alpha '\ne \alpha$ of $(G,\sigma )$ with color *s* and $\rho _\beta \succeq \rho _{\alpha '}$. Moreover, there cannot be any other class $\beta ''$ of color *t* such that $\rho _{\beta ''}$ is contained in the unique path from $\rho _\beta$ to $\rho _\alpha$, otherwise it holds $N(\beta '')=N(\beta )$ and $N^-(\beta '')=N^-(\beta )$ by Lemma 3(vi), i.e., . Therefore, we conclude that $\rho _\beta \rho _\alpha \in E(T_1)$ as well as $\rho _\beta \rho _\alpha \in E(T_2)$.

If *v* is the only leaf of color *s* in $(G,\sigma )$, it follows from (i) and (ii) that $({T_1'},\sigma ')=(T_1(\rho _\beta ),\sigma ')=(T_2(\rho _\beta ),\sigma ') =({T_2'},\sigma ')$; a contradiction, hence there is a unique tree representation for $(G,\sigma )$ if $|L[s]|=1$..

Now suppose that $L[s]>1$. Then, both in case (i) and case (ii) there is a parent of $\mathsf {par}(\rho _\beta )$, because otherwise $(G_1',\sigma ')$ and $(G_2',\sigma ')$ would not contain color *s*. In either case the parent of $\rho _\beta$ is an inner node of the least resolved tree $(T_1,\sigma ')$ and $(T_2,\sigma ')$, respectively. We claim that $\mathsf {par}(\rho _\beta )$ is the root of  class $\gamma$ of color *s*. Suppose this is not the case, i.e., $\sigma (\gamma )=t$ and there is no other $\gamma '\in {\mathscr {N}}$ such that $\sigma (\gamma ')=s$ and $\mathsf {par}(\rho _\beta )=\rho _{\gamma '}$. Then $N(\gamma )=N(\beta )$ and $N^-(\gamma )=N^-(\beta )$ by Lemma 3(vi), which implies that  and $\rho _\beta$ is not the root of $\beta$; a contradiction.

We therefore conclude that the local subtrees of $(T_1,\sigma ')$ and $(T_2,\sigma ')$ immediately above $\alpha$, that is $(T_1(\rho _\gamma ), \sigma '_{|L(T_1(\rho _\gamma ))})$ and $(T_2(\rho _\gamma ), \sigma '_{|L(T_2(\rho _\gamma ))})$, as indicated in Fig. 5, are identical. Moreover, it follows that ${{\,\mathrm{lca}\,}}(u,\gamma )\preceq {{\,\mathrm{lca}\,}}(u,w)$ for any $w\in L[s]{\setminus } \{v\}$. Hence, the additionally inserted edges in $(G_1',\sigma )$ and $(G_2',\sigma )$ are exactly the edges *uc* for all $c\in \gamma$. We therefore conclude that $(G_1',\sigma )=(G_2',\sigma )$, which implies $(T_1',\sigma ')=(T_2',\sigma ')$. Since *v* has been chosen arbitrarily, this implies $(T_1,\sigma )=(T_2,\sigma )$; a contradiction. $\square$

Finally, we consider a few simple properties of least resolved trees that will be useful in the following sections.

#### Corollary 3

Let $(G,\sigma )$ be a connected 2-cBMG that is explained by a least resolved tree $(T,\sigma )$. Then all elements of $\alpha \in {\mathscr {N}}$ are attached to $\rho _\alpha$, i.e., $\rho _\alpha a\in E(T)$ for all $a\in \alpha$.

#### Proof

Assume that $\rho _\alpha a \notin E(T)$. Since by definition $\alpha \prec \rho _\alpha$, there exists an inner node *v* with $\rho _\alpha v \in E(T)$ such that *v* lies in the unique path from $\rho _\alpha$ to *a*. In particular $v\ne a$. Theorem 2 implies that each inner vertex (except possibly the root) of the least resolved tree $(T,\sigma )$ must be the root of some  class of $(G,\sigma )$. Hence, there is a  class $\beta \in {\mathscr {N}}$ with $\rho _\beta =v$. According to Lemma 3(ii), the subtree *T*(*v*) contains leaves of both colors, i.e., there exists some leaf $c\in L(T(v))$ with $\sigma (c)\ne \sigma (a)$. It follows that ${{\,\mathrm{lca}\,}}(a,c)\prec \rho _\alpha$, which contradicts the definition of $\rho _\alpha$. $\square$

This result remains true also for 2-cBMGs that are not connected.

### Characterization of 2-cBMGs

We will first establish necessary conditions for a colored digraph to be a 2-cBMG. The key construction for this purpose is the reachable set of a  class, that is, the set of all leaves that can be reached from this class via a path of directed edges in $(G,\sigma )$. Not unexpectedly, the reachable sets should forms a hierarchical structure. However, this hierarchy does not quite determine a tree that explains $(G,\sigma )$. We shall see, however, that the definition of reachable sets can be modified in such a way that the resulting hierarchy defines the unique least resolved tree w.r.t. $(G,\sigma )$.

#### Necessary conditions

We start by deriving some graph properties of 2-cBMGs. We shall see later that these are in fact sufficient to characterize 2-cBMGs.

##### Theorem 3

Let $(G,\sigma )$ be a connected 2-cBMG. Then, for any two  classes $\alpha$ and $\beta$ of *G* holds

1. $$\alpha \cap N(\beta )=\beta \cap N(\alpha )=\emptyset $$ implies
  $$N(\alpha ) \cap N(N(\beta ))=N(\beta ) \cap N(N(\alpha ))=\emptyset $$.
2. $$N(N(N(\alpha ))) \subseteq N(\alpha )$$
3. $\alpha \cap N(N(\beta ))=\beta \cap N(N(\alpha ))=\emptyset$ and $N(\alpha )\cap N(\beta )\ne \emptyset$ implies $N^-(\alpha )=N^-(\beta )$ and $N(\alpha )\subseteq N(\beta )$ or $N(\beta )\subseteq N(\alpha )$.

##### Proof

(N1) For $\sigma (\alpha )=\sigma (\beta )$ this is trivial, thus suppose $\sigma (\alpha )\ne \sigma (\beta )$. By Lemma 3(vi), $\alpha$ is not contained in the subtree $T(\rho _\beta )$ and $\beta$ is not contained in the subtree $T(\rho _\alpha )$. Therefore, $\rho _\alpha$ and $\rho _\beta$ must be incomparable. Since $N(\alpha ), N(N(\alpha ))\preceq \rho _\alpha$ and $N(\beta ), N(N(\beta ))\preceq \rho _\beta$ by Lemma 3(iii) and (vii), we conclude that $N(\alpha ) \cap N(N(\beta ))= N(\beta ) \cap N(N(\alpha ))=\emptyset$.

(N2) For contradiction, assume that there is $q\in N(N(N(\alpha ))){\setminus } N(\alpha )$. Since $\sigma (q)=\sigma (u)\ne \sigma (x)$ for all $x\in \alpha$ and $u\in N(\alpha )$, any such *q* must satisfy ${{\,\mathrm{lca}\,}}(x,q)\succ {{\,\mathrm{lca}\,}}(x,u)$ for all $x\in \alpha$ and $u\in N(\alpha )$. Otherwise it would be contained in $N(\alpha )$. Since $N(x)\preceq \rho _{\alpha }$ by Lemma 3(iii), the definition of $\rho _{\alpha }$ implies that there is some pair $x\in \alpha$ and $y\in \beta \subseteq N(\alpha )$ with ${{\,\mathrm{lca}\,}}(x,y)=\rho _{\alpha }$. Therefore ${{\,\mathrm{lca}\,}}(x,q)\succ \rho _{\alpha }$.

Now consider $\beta \subseteq N(\alpha )$. Since $\sigma (\beta )\ne \sigma (\alpha )$ and ${{\,\mathrm{lca}\,}}(\alpha ,\beta )\preceq \rho _{\alpha }$, we infer that $N(N(\alpha )) \preceq \rho _{\alpha }$. Repeating the argument yields $N(N(N(\alpha )))\preceq \rho _{\alpha }$ and thus there cannot be a pair of leaves $x\in \alpha$ and $q\in N(N(N(\alpha )))$ with ${{\,\mathrm{lca}\,}}(x,q)\succ \rho _{\alpha }$.

(N3) We first note that (N3) is trivially true for $\alpha =\beta$. Hence, assume $\alpha \ne \beta$ and suppose $N(\alpha )\cap N(\beta )\ne \emptyset$. Since *T* is a tree, Lemma 3(vi) implies that either $N(\alpha )\subseteq N(\beta )$ or $N(\beta )\subseteq N(\alpha )$. Assume $N(\beta )\subseteq N(\alpha )$. Hence, $\rho _\beta \preceq \rho _\alpha$. Consequently, for any $\gamma \subseteq N^-(\alpha )$ holds ${{\,\mathrm{lca}\,}}(\gamma ,\beta )\preceq {{\,\mathrm{lca}\,}}(\gamma ,\alpha )\preceq {{\,\mathrm{lca}\,}}(\gamma ,x)$ for all *x* with $\sigma (x)=\sigma (\alpha )$ and therefore, $N^-(\alpha )\subseteq N^-(\beta )$. Assume for contradiction that there is a $\gamma '\subseteq N^-(\beta ){\setminus } N^-(\alpha )$. By definition, we have $\rho _\alpha \succeq {{\,\mathrm{lca}\,}}(\gamma ',\beta )\succeq \rho _\beta$ in this case. But then, Lemma 3(vi) implies $N(\gamma ')\subseteq N(\alpha )$ and $\beta \subseteq N(\gamma ')\subseteq N(N(\alpha ))$; a contradiction. $\square$

##### Definition 7

For any digraph $(G,\sigma )$ we define the *reachable set*$R(\alpha )$ for a  class $\alpha$ by

1 $$\begin{aligned} R(\alpha ) = N(\alpha ) \cup N(N(\alpha )) \cup N(N(N(\alpha ))) \cup \cdots \end{aligned}$$

Moreover, we write ${\mathscr {W}}:=\{\alpha \in {\mathscr {N}} \mid N^-(\alpha )=\emptyset \}$ for the set of  classes without in-neighbors.

**Fig. 6:**
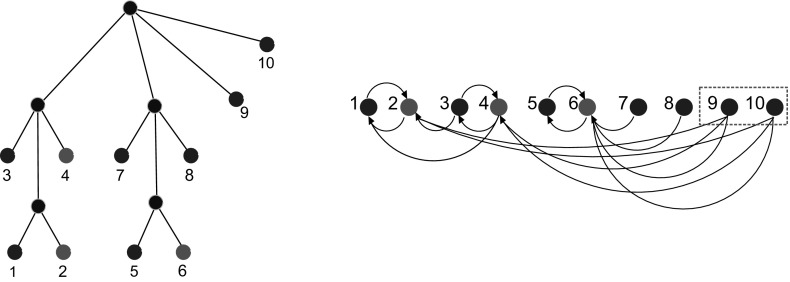
A 2-cBMG with $|{\mathscr {W}}|>1$ and its least resolved tree. The  class $\alpha =\{9,10\}$ consists of children of the root without in-neighbors. There is a second  class without in-neighbors, namely $\beta =\{7,8\}$. Hence ${\mathscr {W}}=\{\alpha ,\beta \}$, $R(\alpha )=\{1,\dots ,6\}=L{\setminus }(\alpha \cup \beta )$, while $R(\beta )=\{5,6\}$

As we shall see below, technical difficulties arise for distinct  classes that share the same set of in-neighbors. Hence, we briefly consider the classes in ${\mathscr {W}}$. An example is shown Fig. 6.

##### Lemma 7

Let $G(T,\sigma )$ be a connected 2-cBMG explained by a tree $(T,\sigma )$. Then all  classes in ${\mathscr {W}}$ have the same color and the cardinality of ${\mathscr {W}}$ distinguishes three types of roots as follows:

(i) ${\mathscr {W}}=\emptyset$ if and only if $\rho _T=\rho _{\alpha }=\rho _{\beta }$ for two distinct  classes $\alpha$ and $\beta$.
(ii) $|{\mathscr {W}}|>1$ if and only if there is a unique  class $\alpha ^*\in {\mathscr {W}}$ that is characterized by $R(\alpha ^*) = L{\setminus }\bigcup _{\beta \in {\mathscr {W}}} \beta$. Furthermore, $\rho _{\alpha ^*}=\rho _T$.
(iii) If ${\mathscr {W}}=\{\alpha \}$, then $\rho _{\alpha }=\rho _T$ and $R(\alpha )=L{\setminus } \alpha$.

##### Proof

By Theorem 1 there is at least one child *v* of the root $\rho _T$ of *T* that itself is the root of a subtree with a single leaf color, i.e., $\sigma (L(T(v)))=\{s\}$. Assume for contradiction that there are two  classes $\alpha ,\beta \in {\mathscr {W}}$ with $s=\sigma (\alpha )\ne \sigma (\beta )=t$. Then by definition ${{\,\mathrm{lca}\,}}(v,x)=\rho _T$ for all $x\in \beta$, and furthermore, $ux \in E(G)$ for all $u\in L(T(v))$. Since $x\in \beta$ has an in-arc, $\beta \not \in {\mathscr {W}}$, a contradiction. All leaves in ${\mathscr {W}}$ therefore have the same color.

For the remainder of the proof we fix such a child *v* of the root $\rho _T$. By construction all leaves below it belong to the same  class, which we denote by $\omega =L(T(v))$. W.l.o.g. we assume $\sigma (v)=s$. Since $\rho _\omega =\rho _T$ by construction, we have $N(\omega )=L[t]$.

(i)   Suppose ${\mathscr {W}}=\emptyset$. Then there is a $\beta \in {\mathscr {N}}_t$ such that $\beta {\subseteq } N^-(\omega )$. For all $b\in \beta$ we have ${{\,\mathrm{lca}\,}}(b,\omega )\le {{\,\mathrm{lca}\,}}(b,x)$ for all $x\in L[s]$. Since ${{\,\mathrm{lca}\,}}(b,\omega )=\rho _T$ we conclude $\rho _\beta =\rho _T=\rho _\omega$.

Conversely, suppose $\alpha$ and $\beta$ are two distinct  classes such that $\rho _\alpha =\rho _\beta =\rho _T$. By Lemma 3(v), $\sigma (\alpha )\ne \sigma (\beta )$. W.l.o.g. assume $\sigma (\alpha )=s$ and $\sigma (\beta )=t$. Since $L(T(\rho _\alpha ))=L(T(\rho _T)=L$, Lemma 3(vi) implies that $N(\alpha )= L[t]$ and $N(\beta )=L[s]$. Therefore, $\alpha \in N^-(\gamma )$ for all $\gamma \in {\mathscr {N}}_t$ and $\beta \in N^-(\gamma )$ for all $\gamma \in {\mathscr {N}}_s$. Hence ${\mathscr {W}}=\emptyset$.

(ii)   If ${\mathscr {W}}\ne \emptyset$, (i) implies $\rho _\beta \ne \rho _T$ for all $\beta \in {\mathscr {N}}_t$, and hence $\rho _\beta \prec \rho _T$. Thus, there is no $\beta \in {\mathscr {N}}_t$ with $\omega \subseteq N(\beta )$, i.e., $N^-(\omega )=\emptyset$ and thus $\omega \in {\mathscr {W}}$.

Consider $\gamma \in {\mathscr {N}}_s$. We have $N^-(\gamma )\ne \emptyset$ if and only if there is $\zeta \in {\mathscr {N}}_t$ such that $\gamma {\subseteq } N(\zeta )$, i.e., if and only if $\gamma \subseteq N(L[t])$. Since $N(\omega )=L[t]$ we have $\gamma \notin {\mathscr {W}}$ if and only if $\gamma \subseteq N(N(\omega ))$. In other words, $N(N(\omega ))=L[s]{\setminus } \bigcup _{\beta \in {\mathscr {W}}}\beta$. Using (N2) we have

$$\begin{aligned} R(\omega )= N(\omega )\cup N(N(\omega )) = L[t]\cup \bigcup \{\gamma \in {\mathscr {N}}_s|N^-(\gamma )\ne \emptyset \} = L{\setminus } \bigcup _{\gamma \in {\mathscr {W}}} \gamma \,. \end{aligned}$$

Now suppose there is another $\alpha \in {\mathscr {W}}$ with $R(\alpha )=L{\setminus } \bigcup _{\gamma \in {\mathscr {W}}} \gamma$. We already know that $\sigma (\alpha )=s$ since all classes in ${\mathscr {W}}$ must have the same color. Hence $L[t]\subseteq R(\alpha )$. Consequently, $\zeta \in N(\omega )$ if and only if $\zeta \in N(\alpha )$ and thus $N(\alpha )=N(\omega )$. Since $\alpha ,\omega \in {\mathscr {W}}$ implies $N^-(\alpha )=N^-(\omega )=\emptyset$, $\alpha$ and $\omega$ share both in- and out-neighbors, and thus $\alpha =\omega$. Therefore $\omega$ is unique.

(iii)   From the proof of (ii), we know that if $|{\mathscr {W}}|=1$, then the unique member of ${\mathscr {W}}$ is $\omega$. We already know that $\rho _\omega =\rho _T$. $\square$

#### Sufficient conditions

We now turn to showing that the properties obtained in Theorem 3 are already sufficient for the characterization of 2-cBMGs. For this we show that the extended reachable sets form a hierarchy whenever $(G,\sigma )$ satisfies the properties (N1), (N2), and (N3).

Recall that a set system ${\mathcal {H}}\subseteq 2^L$ is a *hierarchy* on *L* if (i) for all $A,B\in {\mathcal {H}}$ holds $A\subseteq B$, $B\subseteq A$, or $A\cap B=\emptyset$ and (ii) $L\in {\mathcal {H}}$.

The following simple property we will be used throughout this section:

##### Lemma 8

If *G* is a connected two-colored digraph satisfying (N1), then for any two  classes $\alpha$ and $\beta$ holds

2 $$\begin{aligned} N(\alpha ) \cap N(\beta )=\emptyset \quad \text {implies}\quad N(N(\alpha )) \cap N(N(\beta ))=\emptyset \end{aligned}$$

If *G* satisfies (N2), then $R(\alpha ) = N(\alpha ) \cup N(N(\alpha ))$.

##### Proof

For any $\gamma \subseteq N(\alpha )$ and any $\gamma ' \subseteq N(\beta )$, (N1) implies $N(\gamma )\cap N(N(\beta ))=N(\gamma ')\cap N(N(\alpha ))= \emptyset$. Recall that (N0) holds by definition of  classes. Hence, $N(\alpha )$ is the disjoint union of  classes, i.e., $N(\alpha ) = \bigcup _{\gamma \subseteq N(\alpha )} \gamma$. Thus, $N(N(\alpha )) \cap N(N(\beta ))=(\bigcup _{\gamma \subseteq N(\alpha )} N(\gamma )) \cap N(N(\beta )) =\emptyset$. The equation $R(\alpha ) = N(\alpha ) \cup N(N(\alpha ))$ is an immediate consequence of (N2). $\square$

##### Lemma 9

Let $(G,\sigma )$ be a connected two-colored digraph satisfying properties (N1), (N2), and (N3). Then, ${\mathcal {H}}:=\{R(\alpha )\mid \alpha \in {\mathscr {N}}\}$ is a hierarchy on $L{\setminus } \bigcup _{\alpha \in {\mathscr {W}}}\alpha$.

##### Proof

First we note that $R(\alpha ) = N(\alpha ) \cup N(N(\alpha ))$ by property (N2). Furthermore, using (N0), we observe that $\beta \cap N(\alpha )\ne \emptyset$ implies $\beta \subseteq N(\alpha )$ for all  classes $\alpha$ and $\beta$. In particular, therefore, $N(\alpha )$ is a disjoint union of  classes, and thus $N(N(\alpha ))=\bigcup _{\beta \subseteq N(\alpha )} N(\beta )$ is again a disjoint union of  classes. Hence, for any  class $\beta \ne \alpha$, we have either $\beta \subseteq R(\alpha )$ or $\beta \cap R(\alpha )=\emptyset$. Note that the case $\alpha =\beta$ is trivial.

Suppose first $\beta \subseteq R(\alpha )$. If $\beta \subseteq N(\alpha )$, then $R(\beta ) = N(\beta ) \cup N(N(\beta )) \subseteq N(N(\alpha )) \cup N(N(N(\alpha ))) \subseteq N(N(\alpha ))\cup N(\alpha )$. On the other hand, $\beta \subseteq N(N(\alpha ))$ yields $R(\beta ) \subseteq N(N(N(\alpha ))) \cup N(N(N(N(\alpha ))) \subseteq N(\alpha )\cup N(N(\alpha ))$. Thus, $R(\beta )\subseteq R(\alpha )$.

Exchanging the roles of $\alpha$ and $\beta$, the same argument shows that $\alpha \subseteq R(\beta )$ implies $R(\alpha )\subseteq R(\beta )$.

Now suppose that neither $\alpha \subseteq R(\beta )$ nor $\beta \subseteq R(\alpha )$ and thus, by the arguments above, that $\alpha \cap R(\beta )=\beta \cap R(\alpha )=\emptyset$. In particular, therefore, $\alpha \cap N(\beta )=\beta \cap N(\alpha )=\emptyset$ and thus property (N1) implies $R(\alpha )\cap R(\beta )=(N(\alpha )\cap N(\beta ))\cup (N(N(\alpha ))\cap N(N(\beta )))$. If $N(\alpha )\cap N(\beta )=\emptyset$, then $R(\alpha )\cap R(\beta )=\emptyset$ by Lemma 8. If $N(\alpha )\cap N(\beta )\ne \emptyset$, then property (N3) and $\alpha \cap R(\beta )=\beta \cap R(\alpha )=\emptyset$ implies either $N(\alpha )\subseteq N(\beta )$ or $N(\beta )\subseteq N(\alpha )$. Isotony of *N* thus implies $N(N(\alpha ))\subseteq N(N(\beta ))$ or $N(N(\beta ))\subseteq N(N(\alpha ))$, respectively. Hence we have either $R(\alpha )\subseteq R(\beta )$ or $R(\beta )\subseteq R(\alpha )$. Therefore ${\mathcal {H}}$ is a hierarchy.

Finally, we proceed to show that there is a unique set $R(\alpha ^*)$ that is maximal w.r.t. inclusion and in particular, satisfies $R(\alpha ^*)=L{\setminus } \bigcup _{\alpha \in {\mathscr {W}}} \alpha$.

Assume, for contradiction, that there are two distinct elements $R(\alpha ), R(\alpha ^*)\in {\mathcal {H}}$ that are both maximal w.r.t. inclusion. Thus, $R(\alpha )\cap R(\alpha ^*)=\emptyset$ and $\alpha \ne \alpha ^*$. Moreover, since ${\mathcal {H}}$ is a hierarchy, for each $\beta \in {\mathscr {N}}$ with $R(\beta )\subseteq R(\alpha )$, we must have $R(\beta )\cap R(\alpha ^*)=\emptyset$. In particular, this implies $\beta \subseteq R(\alpha )$ for any $\beta \in {\mathscr {N}}$ with $R(\beta ) \subseteq R(\alpha )$. As a consequence there is no $\beta \subseteq R(\alpha )$ and $\beta '\subseteq R(\alpha ^*)$ such that $\beta \subseteq N(\alpha ^*)$ and $\beta '\subseteq N(\alpha )$, respectively. Therefore, $R(\alpha )$ and $R(\alpha ^*)$ are not connected; a contraction to the connectedness of *G*. Hence, $R(\alpha ) = R(\alpha ^*)$, i.e., the there is a unique set $R(\alpha ^*)$ in ${\mathcal {H}}$ that is maximal w.r.t. inclusion. It contains all  classes of *G* that have non-empty in-neighborhood. Since by definition, all vertices of *G* are assigned to exactly one  class, we conclude that $R(\alpha ^*)=L{\setminus } \bigcup _{\alpha \in {\mathscr {W}}} \alpha$. $\square$

**Fig. 7:**
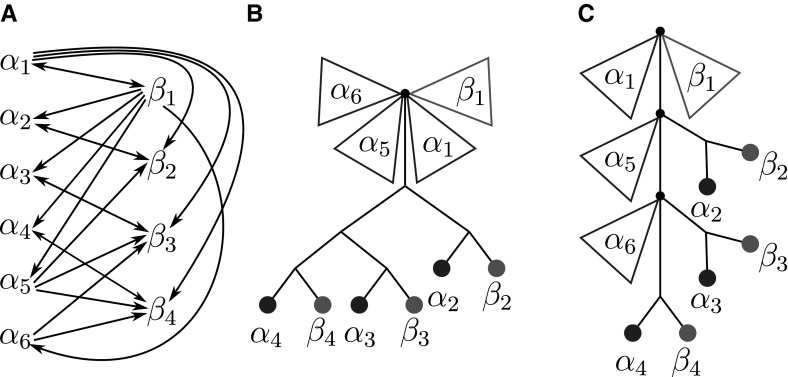
**a** The two-colored digraph $(G,\sigma )$ satisfies (N1), (N2) and (N3). All $\alpha _i$ are  classes of $(G,\sigma )$ and belong to color “blue”, the  classes $\beta _j$ form the “red” color classes. Red (blue) triangles indicate subtrees that only contain red (blue) leaves. Note that $N^-(\alpha _1)=N^-(\alpha _5)=N^-(\alpha _6)$. **b** The tree obtained from the hierarchy ${\mathcal {H}}=\{R(\alpha )\mid \alpha \in {\mathscr {N}}\}$ by attaching to the corresponding tree the elements of $\alpha$ as leaves to $R(\alpha )$ does not explain $(G,\sigma )$. It would imply $N^-(\alpha _1)=N^-(\alpha _5)=N^-(\alpha _6)$ and $N(\alpha _1)=N(\alpha _5)=N(\alpha _6)$, i.e., . **c** The tree defined by the hierarchy ${\mathcal {H'}}=\{R'(\alpha )\mid \alpha \in {\mathscr {N}}\}$ with elements of $\alpha$ attached as leaves to $R'(\alpha )$ is the unique least resolved tree that explains *G* (cf. Lemma 11) (color figure online)

Note that while $R(\alpha )$ is unique for a given  class $\alpha$, there may exist more than one  class that have the same reachable set (see for instance $\alpha _2$ and $\beta _2$ in Fig. 7c). In particular, there may even be  classes with different color giving rise to the same element of ${\mathcal {H}}$. More generally, we have $R(\alpha )=R(\beta )$ for $\alpha \ne \beta$ if and only if $\alpha \in R(\beta )$ and $\beta \in R(\alpha )$.

A hierarchy ${\mathcal {H}}$ corresponds to a unique tree $T({\mathcal {H}})$ defined as the Hasse diagram of ${\mathcal {H}}$, i.e., the vertices of $T({\mathcal {H}})$ are sets of ${\mathcal {H}}$, and $R_2$ is a child of $R_1$ iff $R_2\subset R_1$ and there is no $R_3$ such that $R_2\subset R_3\subset R_1$. In particular, thus, two  classes belong to the same interior vertex if $R(\alpha )=R(\beta )$. It is tempting to use this tree to construct a tree *T* explaining $(G,\sigma )$ by attaching the elements of $\alpha$ as leaves to the node $R(\alpha )$ in $T({\mathcal {H}})$. The example in Fig. 7a, b shows, however, that this simply does not work. The key issue arises from groups of distinct  classes that share the same in-neighborhood because they will in general be attached to the same node in $T({\mathcal {H}})$, i.e., they are indistinguishable. We therefore need a modification of the definition of reachable sets that properly distinguishes such  classes in order to construct a hierarchy with the appropriate resolution for the least resolved tree specified in Theorem 2. To this end we define for every  class the auxiliary leaf set

3 $$\begin{aligned} Q(\alpha ) = \{x\in L \,\mid \, \exists \beta \in {\mathscr {N}}:\, x\in \beta ,\, N^-(\beta )=N^-(\alpha ) \text { and } N(\beta )\subseteq N(\alpha )\} \end{aligned}$$

Note that $\alpha \subseteq Q(\alpha )$. For later reference we list several simple properties of *Q*.

##### Lemma 10

(i) $$\beta \subseteq Q(\alpha )$$ implies $$\sigma (\beta )=\sigma (\alpha )$$.
(ii) $$\beta \subseteq Q(\alpha )$$ implies $$Q(\beta )\subseteq Q(\alpha )$$.
(iii) $$\beta \subseteq Q(\alpha )$$ implies $$R(\beta )\subseteq R(\alpha )$$.
(iv) $$\alpha \cap N(\beta )=\emptyset $$ implies $$Q(\alpha )\cap \ N(\beta )=\emptyset $$.
(v) $$\alpha \cap N(N(\beta ))=\emptyset $$ implies $$Q(\alpha )\cap \ N(N(\beta ))=\emptyset $$.

##### Proof

(i) follows directly from the definition.

(ii) Let $\beta \subseteq Q(\alpha )$, $\gamma \in {\mathscr {N}}$ and $\gamma \subseteq Q(\beta )$. Then, $N^-(\gamma )=N^-(\beta )=N^-(\alpha )$ and $N(\gamma )\subseteq N(\beta )\subseteq N(\alpha )$, hence $\gamma \subseteq Q(\alpha )$ and therefore $Q(\beta )\subseteq Q(\alpha )$.

(iii) By definition, $N(\beta )\subseteq N(\alpha )$. Monotonicity of *N* implies $N(N(\beta ))\subseteq N(N(\alpha )$) and therefore, $R(\beta )\subseteq R(\alpha )$.

(iv) Assume that $\alpha \cap N(\beta )=\emptyset$, but $\gamma \subseteq Q(\alpha )\cap \ N(\beta )\ne \emptyset$. Thus, $\beta \subseteq N^-(\gamma )=N^-(\alpha )$, i.e., $\alpha \subseteq N(\beta )$; a contradiction.

(v) Assume that $\alpha \cap N(N(\beta ))=\emptyset$, but $\gamma \subseteq Q(\alpha )\cap \ N(N(\beta ))\ne \emptyset$. Thus, there is a  class $\xi \subseteq N(\beta )$ such that $\xi \subseteq N^-(\gamma )=N^-(\alpha )$ and therefore, $\alpha \subseteq N(N(\beta ))$; a contradiction. $\square$

Finally we define, for any two-colored digraph $(G,\sigma )$, its *extended reachable set* as

4 $$\begin{aligned} R'(\alpha ):= R(\alpha )\cup Q(\alpha ). \end{aligned}$$

Note that $\alpha \in R'(\alpha )$. Furthermore, the extended reachable set $R'(\alpha )$ contains vertices with both colors for every  class $\alpha$. Thus $|R'(\alpha )|>1$. We show next that for any 2-cBMG the extended reachable sets form the hierarchy that yields the desired least resolved tree.

##### Lemma 11

Let $(G,\sigma )$ be a connected two-colored digraph satisfying properties (N1), (N2), and (N3). Then, ${\mathcal {H'}}:=\{R'(\alpha )\mid \alpha \in {\mathscr {N}}\}$ is a hierarchy on *L*.

##### Proof

Consider two distinct  classes $\alpha ,\beta \in {\mathscr {N}}$. By definition $Q(\alpha )$ is the disjoint union of  classes. The same is true for $R(\alpha )$ as argued in the proof of Lemma 9, hence $R'(\alpha )=R(\alpha )\cup Q(\alpha )$ is also the disjoint union of  classes. Thus we have either $\beta \subseteq R'(\alpha )$ or $\beta \cap R'(\alpha )=\emptyset$.

First assume $\beta \subseteq R'(\alpha )$. Thus we have $\beta \subseteq R(\alpha )$ or $\beta \subseteq Q(\alpha )$. If $\beta \subseteq Q(\alpha )$, i.e., $N(\beta )\subseteq N(\alpha )$ and consequently $R(\beta )\subseteq R({\alpha })$, then Lemma 10(ii) + (iii) implies that $R'(\beta )\subseteq R'(\alpha )$. If $\beta \subseteq R(\alpha )$ then $R(\beta )\subseteq R(\alpha )\subseteq R'(\alpha )$, shown as in the proof of Lemma 9. It remains to show that $Q(\beta )\subseteq R'(\alpha )$. By definition, we have $N^-(\gamma )=N^-(\beta )$ for any $\gamma \subseteq Q(\beta )$. Therefore, $\beta \subseteq N(\alpha ) \cup N(N(\alpha ))$ implies $\gamma \subseteq N(\alpha )\cup N(N(\alpha ))$. Hence, $\gamma \subseteq R(\alpha )\subseteq R'(\alpha )$. In summary, for all $\beta \subseteq R'(\alpha )$ we have $R'(\beta )\subseteq R'(\alpha )$.

The implication “$\alpha \subseteq R'(\beta ) \implies R'(\alpha )\subseteq R'(\beta )$” follows by exchanging $\alpha$ and $\beta$ in the previous paragraph.

Now suppose $\beta \cap R'(\alpha )=\alpha \cap R'(\beta )=\emptyset$. In particular, it then holds $\alpha \cap N(\beta )=\beta \cap N(\alpha )=\emptyset$ and $\alpha \cap N(N(\beta ))=\beta \cap N(N(\alpha ))=\emptyset$. Applying property (N1) and Lemma 10(iv) + (v) yields $R'(\alpha )\cap R'(\beta )= \big (N(\alpha )\cap N(\beta )\big )\cup \big (N(N(\alpha ))\cap N(N(\beta ))\big ) \cup \big (Q(\alpha )\cap Q(\beta )\big )$. First, let $N(\alpha )\cap N(\beta )=\emptyset$. This immediately implies $Q(\alpha )\cap Q(\beta )=\emptyset$ and from Lemma 8 follows $N(N(\alpha ))\cap N(N(\beta ))=\emptyset$. Hence, $R'(\alpha )\cap R'(\beta )=\emptyset$. Now assume $N(\alpha )\cap N(\beta )\ne \emptyset$. By property (N3) we conclude $N^-(\alpha )=N^-(\beta )$ and either $N(\alpha )\subseteq N(\beta )$ or $N(\beta )\subseteq N(\alpha )$. Consequently, either $N(N(\alpha ))\subseteq N(N(\beta ))$ and $Q(\alpha )\subseteq Q(\beta )$, or $N(N(\beta ))\subseteq N(N(\alpha ))$ and $Q(\beta )\subseteq Q(\alpha )$. Hence, it must either hold $R'(\alpha )\subseteq R'(\beta )$ or $R'(\beta )\subseteq R'(\alpha )$.

It remains to show that $L\in {\mathcal {H'}}$. Similar arguments as in the proof of Lemma 9 can be applied in order to show that there is a unique element $R'(\alpha ^*)$ that is maximal w.r.t. inclusion in ${\mathcal {H'}}$. Since for any $\alpha \in {\mathscr {N}}$ it is true that $\alpha \in R'(\alpha )$, every  class of *G* is contained in at least one element of ${\mathcal {H'}}$. Moreover, any vertex of *G* is contained in exactly one  class. Hence, $L=R'(\alpha ^*)\in {\mathcal {H'}}$. $\square$

Since ${\mathcal {H'}}$ is a hierarchy, its Hasse diagram is a tree $T({\mathcal {H'}})$. Its vertices are by construction exactly the extended reachable sets $R'(\alpha )$ of $(G,\sigma )$. Starting from $T({\mathcal {H'}})$, we construct the tree $T^*({\mathcal {H'}})$ by attaching the vertices $x{\in \alpha }$ to the vertex $R'(\alpha )$ of $T({\mathcal {H'}})$. The tree $T^*({\mathcal {H'}})$ has leaf set *L*. Since $|R'(\alpha )|>1$ as noted below Eq. (4), $T^*({\mathcal {H'}})$ is a phylogenetic tree.

##### Theorem 4

Let $(G,\sigma )$ be a connected 2-colored digraph. Then there exists a tree *T* explaining $(G,\sigma )$ if and only if *G* satisfies properties (N1), (N2), and (N3). The tree $T^*({\mathcal {H'}})$ is the unique least resolved tree that explains $(G,\sigma )$.

##### Proof

The “only if”-direction is an immediate consequence of Lemma 2 and Theorem 3. For the “if”-direction we employ Lemma 11 and show that the tree $T^*({\mathcal {H'}})$ constructed from the hierarchy ${\mathcal {H'}}$ explains $(G,\sigma )$.

Let $x\in L$ and $\alpha$ be the  class of $(G,\sigma )$ to which *x* belongs. Denote by $\tilde{N}(x)$ the out-neighbors of *x* in the graph explained by $T^*({\mathcal {H'}})$. Therefore $y\in \tilde{N}(x)$ if and only if $\sigma (y)\ne \sigma (x)$ and ${{\,\mathrm{lca}\,}}_{T^*({\mathcal {H}}')}(x,y)$ is the interior node to which *x* is attached in $T({\mathcal {H'}})$, i.e., $R'(\alpha )$. Therefore, $y\in \tilde{N}(x)$ if and only if $\sigma (y)\ne \sigma (x)$ and $y\in R'(\alpha )$. By (N2) this is the case if and only if $y\in N(x)$. Thus $\tilde{N}(x)=N(x)$. Since two digraphs are identical whenever all their out-neighborhoods are the same, the tree $T^*({\mathcal {H'}})$ indeed explains $(G,\sigma )$.

By construction and Theorem 2, $(T^*({\mathcal {H'}}),\sigma )$ is a least resolved tree. $\square$

### Informative triples

An inspection of induced three-vertex subgraphs of a 2-cBMG $(G,\sigma )$ shows that several local configurations derive only from specific types of trees. More precisely, certain induced subgraphs on three vertices are associated with uniquely defined triples displayed by the least resolved tree $(T,\sigma )$ introduced in the previous section. Other induced subgraphs on three vertices, however, may derive from two or three distinct triples. The importance of triples derives from the fact that a phylogenetic tree can be reconstructed from the triples that it displays by a polynomial time algorithm traditionally referred to as BUILD (Semple and Steel 2003).

BUILD makes use of a simple graph representation of certain subsets of triples: Given a triple set *R* and a subset of leaves $L'\subseteq L$, the *Aho-graph*$[R,L']$ has vertex set $L'$ and there is an edge between two vertices $x,y\in L'$ if and only if there exists a triple $xy|z\in R$ with $z\in L'$ (Aho et al. 1981). It is well known that *R* is consistent if and only if $[R,L']$ is disconnected for every subset $L'\subseteq L$ with $|L'|>1$ (Bryant and Steel 1995). BUILD uses Aho-graphs in a top-down recursion: First, [*R*, *L*] is computed and a tree *T* consisting only of the root $\rho _T$ is initialized. If [*R*, *L*] is connected and $|L|>1$, then BUILD terminates and returns “*Ris not consistent”*. Otherwise, BUILD adds the connected components $C_1,\dots ,C_k$ of [*R*, *L*] as vertices to *T* and inserts the edges $(\rho _T,C_i)$, $1\le i\le k$. BUILD recurses on the Aho-graphs $[R,C_i]$ (where vertex $C_i$ in *T* plays the role of $\rho _T$) until it arrives at single-vertex components. BUILD either returns the tree *T* or identifies the triple set *R* as “not consistent”. Since the Aho-graphs $[R,L']$ and their connected components are uniquely defined in each step of $\texttt {BUILD}$, the tree *T* is uniquely defined by *R* whenever it exists. *T* is known as the *Aho tree* and will be denoted by ${{\,\mathrm{Aho}\,}}(R)$.

It is natural to ask whether the triples that can be inferred directly from $(G,\sigma )$ are sufficient to (a) characterize 2-cBMGs and (b) to completely determine the least resolved tree $(T,\sigma )$ explaining $(G,\sigma )$.

#### Definition 8

**Fig. 8:**
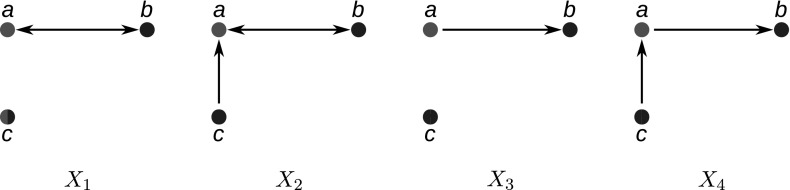
Each of the three-vertex induced subgraphs $X_1$, $X_2$, $X_3$ and $X_4$ gives a triple *ab*|*c*. If vertex *c* i n the drawing has two colors, then the color $\sigma (c)$ does not matter

Let $(G,\sigma )$ be a two-colored digraph. We say that a triple *ab*|*c* is *informative* (for $(G,\sigma )$) if the three distinct vertices $a,b,c\in L$ induce a colored subgraph *G*[*a*, *b*, *c*] isomorphic (in the usual sense, i.e., with recoloring) to the graphs $X_1$, $X_2$, $X_3$, or $X_4$ shown in Fig. 8. The set of informative triples is denoted by ${\mathcal {R}}(G,\sigma )$.

#### Lemma 12

If $(G,\sigma )$ is a connected 2-cBMG, then each triple in ${\mathcal {R}}(G,\sigma )$ is displayed by any tree *T* that explains $(G,\sigma )$.

#### Proof

Let $(T,\sigma )$ be a tree that explains $(G,\sigma )$. Assume that there is an induced subgraph $X_1$ in $(G,\sigma )$. W.l.o.g. let $\sigma (c) = \sigma (b)$. Since there is no arc (*a*, *c*) but an arc (*a*, *b*), we have ${{\,\mathrm{lca}\,}}(a,b)\prec {{\,\mathrm{lca}\,}}(a,c)$, which implies that *T* must display the triple *ab*|*c*. By the same arguments, if $X_2$, $X_3$ or $X_4$ is an induced subgraph in $(G,\sigma )$, then *T* must display the triple *ab*|*c*. $\square$

**Fig. 9:**
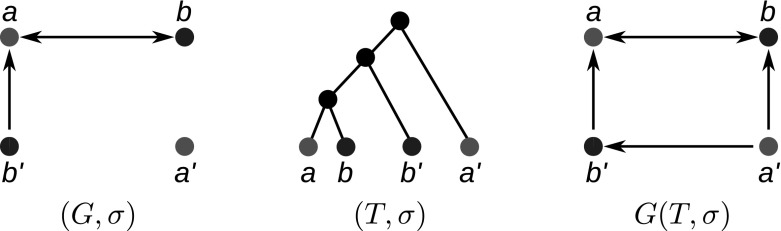
The four-vertex graph $(G,\sigma )$ on the l.h.s. cannot be a 2-cBMG because there is no out-arc from $a'$. The four induced subgraphs are of type $X_1$, $X_2$, $X_3$ (with red and blue exchanged) and arc-less, respectively resulting in the set $R(G,\sigma ) = \{ab|b',ab|a',ab'|a'\}$ of informative triples. This set is consistent and displayed by the Aho tree *T* shown in the middle. It is not difficult to check that every edge of *T* is distinguished by one informative triple. Therefore $R(G,\sigma )$ identifies the leaf-colored tree $(T,\sigma )$ (Grünewald et al. 2007). However, the graph $G(T,\sigma )$ explained by the tree $(T,\sigma )$ is not isomorphic to the graph $(G,\sigma )$ from which the triples were inferred (color figure online)

In particular, therefore, if $(G,\sigma )$ is 2-cBMG, then ${\mathcal {R}}(G,\sigma )$ is consistent. It is tempting to conjecture that consistency of the set ${\mathcal {R}}(G,\sigma )$ of informative triples is already sufficient to characterize a 2-cBMG. The example in Fig. 9 shows, however, that this is not the case.

#### Lemma 13

Let $(T,\sigma )$ be a least resolved tree explaining a connected 2-cBMG $(G,\sigma )$. Then every inner edge of *T* is distinguished by at least one triple in ${\mathcal {R}}(G,\sigma )$.

#### Proof

Let $(T,\sigma )$ be a least resolved tree w.r.t. to $(G,\sigma )$ and $e=uv$ be an inner edge of *T*. Since $(T,\sigma )$ is least resolved for $(G,\sigma )$, Theorem 2 implies that the edge *e* is relevant, and hence, there exists a $\alpha \in {\mathscr {N}}$ such that $v=\rho _\alpha$. By Corollary 3, we have $a\in \mathsf {child}(v)$ for any $a\in \alpha$. Lemma 3(ii) implies that *T*(*v*) contains a  class $\beta$ with $\sigma (\alpha )\ne \sigma (\beta )$ and $b\in \beta$.

*Case A:* Suppose that $\rho _\beta =\rho _\alpha$ and therefore, $ab, ba \in E(G)$. If *u* is the root of some  class with $c\in \gamma$, then Lemma 3(vi) implies $ca\in E(G)$, $cb\notin E(G)$ for $\sigma (c)=\sigma (b)$ and $cb\in E(T)$, $ca\notin E(T)$ for $\sigma (c)=\sigma (a)$. In all cases, we have neither $bc\in E(G)$ nor $ac\in E(G)$, since $ab, ba \in E(G)$. Therefore, we always obtain a 3-vertex induced subgraph that is isomorphic to $X_2$ (see Fig. 8) and $ab|c \in {\mathcal {R}}(G,\sigma )$. On the other hand, if there is no  class $\gamma$ such that $u=\rho _\gamma$, then *u* is the root of $(T,\sigma )$ by Corollary 3. Since $(T,\sigma )$ is phylogenetic and *u* is no root of any  class, there must be an inner vertex $w\in \mathsf {child}(u){\setminus } {\{v\}}$ such that $w=\rho _\gamma$ for some $\gamma \in {\mathscr {N}}$. Since $T(\rho _\gamma )$ contains leaves of both colors by Lemma 3(ii), for any leaf $c\in L(T(\rho _\gamma ))$ there is no edge between *c* and *b* as well as between *c* and *a*. Taken together, we obtain the induced subgraph $X_1$ and the triple *ab*|*c*.

*Case B:* Now assume $\rho _\beta \prec \rho _\alpha$ and there is no other $\beta '\in {\mathscr {N}}$ with $\sigma (\beta ')=\sigma (\beta )$ and $\rho _\alpha =\rho _{\beta '}$. By definition of $\rho _\beta$, we have ${{\,\mathrm{lca}\,}}(b,a')\prec {{\,\mathrm{lca}\,}}(b,a)$ for some $a'$ with $\sigma (a)=\sigma (a')$, i.e., $ba\notin E(G)$. Moreover, Lemma 3(vi) implies $b\in N(a)$, thus $ab\in E(G)$. Similar to Case A, first suppose that *u* is the root of some  class of $(G,\sigma )$. Since *e* is relevant, there is a $\gamma \in {\mathscr {N}}$ with $u=\rho _\gamma$ and $\sigma (\gamma )\ne \sigma (\alpha )$. Otherwise, if $\sigma (\gamma )= \sigma (\alpha )$ and there is no other $\gamma '\in {\mathscr {N}}$ with $u=\rho _{\gamma '}$, Lemma 3(vi) implies $N(\alpha )=N(\gamma )$ and $N^-(\alpha )=N^-(\gamma )$, i.e., $\alpha$ and $\gamma$ belong to the same  class with root *u*. Hence, *v* is not the root of any  class; a contradiction. Consequently, we have $\sigma (\gamma )\ne \sigma (\alpha )$, thus $ca\in E(G)$ by Lemma 3(vi) but $ac\notin E(G)$. This yields the triple *ab*|*c* that is derived from the subgraph $X_4$. If *u* is no root of any  class, analogous arguments as in *Case A* show that there is an inner vertex $w\in \mathsf {child}(u){\setminus } v$ such that the tree *T*(*w*) contains leaves of both colors. In particular, there exists a leaf $c\in L(T(w))$ and since *u* is not the root of $\alpha$, $\beta$ or the  class that *c* belongs to, there is no arc between *c* and *a* or *b* in $(G,\sigma )$. Hence, we again obtain the triple *ab*|*c* which in this case is derived from $X_3$.

In every case we have $v={{\,\mathrm{lca}\,}}(a,b)\prec {{\,\mathrm{lca}\,}}(a,c)=u$, i.e., the triple *ab*|*c* distinguishes *uv*. $\square$

Lemma 13 suggests that the leaf-colored Aho tree $({{\,\mathrm{Aho}\,}}({\mathcal {R}}(G,\sigma )),\sigma )$ of the set of informative triples ${\mathcal {R}}(G,\sigma )$ explains a given 2-cBMG $(G,\sigma )$. The following result shows that this is indeed the case and sets the stage for the main result of this section, a characterization of 2-cBMGs in terms of informative triples.

#### Theorem 5

Let $(G,\sigma )$ be a connected 2-cBMG. Then $(G,\sigma )$ is explained by the Aho tree of the set of informative triples, i.e., $(G,\sigma )=G({{\,\mathrm{Aho}\,}}({\mathcal {R}}(G,\sigma )),\sigma )$.

#### Proof

Let $({\tilde{T}}, \sigma )$ be the unique least resolved tree that explains $(G,\sigma )$. For a fixed vertex $v\in L$ we write $(G',\sigma ')=(G{\setminus } \{v\},\sigma _{|L{\setminus }\{v\}})$. Let $({\tilde{T}}', \sigma ')$ be the unique least resolved tree that explains $(G',\sigma ')$ and let $(T',\sigma '):=({{\,\mathrm{Aho}\,}}({\mathcal {R}}(G',\sigma ')),\sigma ')$ be the leaf-colored Aho tree of the informative triples of $(G',\sigma ')$.

First consider the case $L=\{x,y\}$. Since $(G,\sigma )$ is a connected 2-cBMG, we have $\sigma (x)\ne \sigma (y)$ and $xy,yx \in E(G)$. It is easy to see that both the least resolved tree w.r.t. $(G,\sigma )$ and ${{\,\mathrm{Aho}\,}}({\mathcal {R}}(G,\sigma ))$ correspond to the path $x-\rho _T-y$ with end points *x* and *y*. Thus $(G,\sigma )=G({{\,\mathrm{Aho}\,}}({\mathcal {R}}(G,\sigma )),\sigma )$.

Now let $|L|>2$ and assume that the statement of the proposition is false. Then there is a minimal graph $(G,\sigma )$ such that $(G,\sigma )\ne G(T,\sigma )$, i.e., $(G',\sigma ')=G(T',\sigma ')$ holds for every choice of $v\in V(G)$. Since $(G,\sigma )$ is connected, Theorem 1 implies that there is a  class $\alpha$ of $(G,\sigma )$ such that $\rho _\alpha =\rho _{{\tilde{T}}}$. We fix a vertex *v* in this class $\alpha$ and proceed to show that $(G,\sigma )=G(T,\sigma )$, a contradiction. Let $\sigma (\alpha )=s$ and let $({\tilde{T}}-v,\sigma ')$ be the tree that is obtained by removing the leaf *v* and its incident edge from $({\tilde{T}},\sigma )$. Clearly, the out-neighborhood of every leaf of color *s* is still the same in $({\tilde{T}}-v,\sigma ')$ compared to $({\tilde{T}}, \sigma )$. Moreover, Lemma 3(vi) implies that *N*(*x*) remains unchanged in $({\tilde{T}}-v,\sigma ')$ for any $x\in L[t]{\setminus } \{v\}$ that belongs to a  class $\beta$ with $\rho _\beta \ne \rho _{{\tilde{T}}}$. If $\rho _\beta = \rho _{{\tilde{T}}}$, then $N(x)=L[s]$ in $({\tilde{T}},\sigma )$ by Lemma 3(vi) and thus $N(x)=L[s]{\setminus } \{v\}$ in $({\tilde{T}}-v,\sigma ')$. We can therefore conclude that $({\tilde{T}}-v,\sigma ')$ explains the induced subgraph $(G',\sigma ')$ of $(G,\sigma )$.

Now, we distinguish two cases:

*Case A:* Let $|\mathsf {child}(\rho _{{\tilde{T}}})\cap L|>1$, which implies $|\mathsf {child}(\rho _{{\tilde{T}}-v})\cap L|\ge 1$. Hence, the root of $({\tilde{T}}-v, \sigma ')$ has at least two children and, in particular, $G({\tilde{T}}-v, \sigma ')$ is connected by Theorem 1. Since $({\tilde{T}},\sigma )$ is least resolved, Theorem 2 implies that any inner edge of $({\tilde{T}}-v,\sigma ')$ is non-redundant, and hence $({\tilde{T}}',\sigma ')=({\tilde{T}}-v,\sigma ')$. Consequently, we can recover $({\tilde{T}}, \sigma )$ from $({\tilde{T}},\sigma ')$ by inserting the edge $\rho _{{\tilde{T}}'}v$. If $N^-(\alpha )=\emptyset$, then $vx\in E(G)$ but $xv\notin E(G)$ for any $x\in L[t]$. Hence, any informative triple that contains *v* is induced by $X_2$ or $X_4$, and is thus of the form *xy*|*v* with $\sigma (x)\ne \sigma (y)$. This implies $v\in \mathsf {child}(\rho _T)$. On the other hand, if there is a $\beta \in {\mathscr {N}}$ with $\sigma (\beta )=t$ and $\rho _\beta =\rho _{{\tilde{T}}}$, we have $vu\in E(G)$ and $uv\in E(G)$ with $u\in L[t]$ if and only if $u\in \beta$ by Lemma 4(i). Then, there is no 3-vertex induced subgraph of $(G,\sigma )$ of the form $X_1$, $X_2$, $X_3$, or $X_4$ that contains both *u* and *v*, and any informative triple that contains either *u* or *v* is again of the form *xy*|*v* and *xy*|*v* respectively. As before, this implies $v\in \mathsf {child}(\rho _T)$. Hence, $(T,\sigma )$ is obtained from $(T',\sigma ')$ by insertion of the edge $\rho _{T'}v$. Since $(G',\sigma ')=G(T',\sigma ')$, we conclude that $(T,\sigma )$ explains $(G,\sigma )$, and arrive to the desired contradiction.

*Case B:* If $|\mathsf {child}(\rho _{{\tilde{T}}})\cap L|=1$, then $({\tilde{T}}-v, \sigma ')$ is not least resolved since either (a) the root is of degree 1 or (b) there exists no $u\in \mathsf {child}(\rho _{{\tilde{T}}}){\setminus } \{v\}$ such that $\sigma (u)\ne \{s,t\}$ (see Theorem 1). In the latter case, the graph $(G',\sigma ')$ is not connected. To convert $({\tilde{T}}-v,\sigma ')$ into the least resolved tree $({\tilde{T}}',\sigma ')$, we need to contract all edges $\rho _{{\tilde{T}}}u$ with $u\in \mathsf {child}(\rho _{T'}){\setminus }\{v\}$. Clearly, we can recover $(G,\sigma )$ from $(G',\sigma ')$ by reverting the prescribed steps. Analogous arguments as in *Case A* show that again any informative triple in ${\mathcal {R}}(G,\sigma )$ that contains *v* is of the form *xy*|*v* with $\sigma (x)\ne \sigma (y)$. If $(G'\sigma ')$ is connected, then any triple in ${\mathcal {R}}(G,\sigma ){\setminus } {\mathcal {R}}(G',\sigma ')$ is of this form and hence as above, we conclude that $v\in \mathsf {child}(\rho _T)$ and $(G,\sigma )=G(T,\sigma )$. If $(G'\sigma ')$ is not connected, then ${\mathcal {R}}(G,\sigma ){\setminus } {\mathcal {R}}(G',\sigma ')$ contains also all triples *xy*|*z* induced by $X_1$ and $X_3$ that emerged from connecting all components of $(G',\sigma ')$ by insertion of *v*. However, since ${{\,\mathrm{lca}\,}}(x,y,z)=\rho _{{\tilde{T}}}$, we conclude that $v\in \mathsf {child}(\rho _T)$ and thus $(G,\sigma )=G(T,\sigma )$ again yields the desired contradiction. $\square$

We finally arrive at the main result of this section.

#### Theorem 6

A connected 2-colored digraph $(G,\sigma )$ is a 2-cBMG if and only if $(G,\sigma )=G({{\,\mathrm{Aho}\,}}({\mathcal {R}}(G,\sigma )),\sigma )$.

#### Proof

If $(G,\sigma )$ is a 2-cBMG, then Theorem 5 guarantees that $(G,\sigma )=G({{\,\mathrm{Aho}\,}}({\mathcal {R}}(G,\sigma )),\sigma )$. If $(G,\sigma )$ is not a 2-cBMG, then either ${\mathcal {R}}(G,\sigma )$ is inconsistent or its Aho tree ${{\,\mathrm{Aho}\,}}({\mathcal {R}}(G,\sigma ))$ explains a different graph $G(T,\sigma )\ne (G,\sigma )$ because by assumption $(G,\sigma )$ cannot be explained by any tree. $\square$

If $(G,\sigma )$ is not connected, then the informative triples of Definition 8 are not sufficient by themselves to infer a tree that explains $(G,\sigma )$. However, it follows from Theorems 1 and 6, that the desired tree $(T,\lambda )$ can be obtained by attaching the Aho trees of the connected components as children of the root of $(T,\lambda )$. It can be understood as the Aho tree of the triple set

5 $$\begin{aligned} {\mathcal {R}}(G,\sigma ) = \bigcup _i {\mathcal {R}}(G_i,\sigma _i) \cup {\mathcal {R}}_C(G,\sigma ) \end{aligned}$$

where the ${\mathcal {R}}(G_i,\sigma _i)$ are the sets of informative triples of the connected components and ${\mathcal {R}}_C(G,\sigma )$ consists of all triples of the form *xy*|*z* with $x,y\in L(G_i)$ and $z\in L(G_j)$ for all pairs $i\ne j$. The triple set ${\mathcal {R}}_C(G,\sigma )$ simply specifies the connected components of $(G,\sigma )$. Note that with this augmented definition of ${\mathcal {R}}$, Theorem 6 remains true also for 2-cBMGs that are not connected.

## ***n***-Colored best match graphs

In this section we generalize the results about 2-cBMGs to an arbitrary number of colors. As in the two-color case, we write  if and only if *x* and *y* have the same in- and out-neighbors. Moreover, for given colors $r,s,t\in S$ we write $(G_{st},\sigma _{st}):=G[L[s]\cup L[t]]$ and $(G_{rst},\sigma _{rst}):=G[L[r]\cup L[s]\cup L[t]]$ for the respective induced subgraphs. Since *G* is multipartite and every vertex has at least one out-neighbor of each color except its own, we can conclude also for general cBMGs that  implies $\sigma (x)=\sigma (y)$. Denote by  the thinness relation of Definition 3 on $(G_{st},\sigma _{st}):=G[L[s]\cup L[t]]$.

### Observation 3

If $\sigma (x)=\sigma (y)=s$, then  holds if and only if  for all $t\ne s$.

We can therefore think of the relation  as the common refinement of the relations  based on the induced 2-cBMGs for all colors *s*, *t*. In particular, therefore, all elements of a  class of an *n*-cBMG appear as sibling leaves in the different least resolved trees, each explaining one of the induced 2-cBMGs. Next we generalize the notion of roots.

### Definition 9

Let $(G,\sigma )$ be an *n*-cBMG and suppose $\sigma (\alpha )=r\ne s$. Then the *root*$\rho _{\alpha }$*of the**class*$\alpha$*with respect to colors* is

$$\begin{aligned} \rho _{\alpha ,s}= \max _{\begin{array}{c} x\in \alpha \\ y\in N_s(\alpha ) \end{array}} {{\,\mathrm{lca}\,}}(x,y). \end{aligned}$$

### Observation 4

Consider an *n*-cBMG $(G,\sigma )$ that is explained by a tree $(T,\sigma )$. By Observation 1, the subgraph $(G_{st},\sigma _{st})$ induced by any two distinct colors $s,t\in S$ is a 2-BMG and thus explained by a corresponding least resolved tree $(T_{st},\sigma _{st})$. Uniqueness of this least resolved tree implies that the tree $(T,\sigma )$ must display $(T_{st},\sigma _{st})$. In other words, $(T,\sigma )$ is a refinement of $(T_{st},\sigma _{st})$.

### Observation 5

Let $(G,\sigma )$ be an *n*-cBMG that is explained by a tree $(T,\sigma )$, and $a,b,c \in L$ leaves of three distinct colors. Then the 3-cBMG $(G(T_{\{a,b,c\}}),\sigma )$ is the complete graph on $\{a,b,c\}$ with bidirectional edges.

Therefore, no further refinement can be obtained from triples of three different colors. Thus, the two-colored triples inferred from the induced 2-cBMGs for all color pairs may already be sufficient to construct $(T,\sigma )$. This suggests, furthermore, that every *n*-cBMG is explained by a unique least resolved tree. An important tool for addressing this conjecture is the following generalization of condition (vi) of Lemma 3.

### Lemma 14

Let $(G,\sigma )$ be a (not necessarily connected) *n*-cBMG explained by $(T,\sigma )$ and let $\alpha$ be a  class of $(G,\sigma )$. Then $N_s(\alpha )=L(T(\rho _{\alpha ,s}))\cap L[s]$ for all $s\in S{\setminus } \{\sigma (\alpha )\}$.

### Proof

The definition of $\rho _{\alpha ,s}$ implies $N_s(\alpha )\subseteq L(T(\rho _{\alpha ,s}))\cap L[s]$. In particular, there is a leaf $y\in N_s(\alpha )$ such that ${{\,\mathrm{lca}\,}}(y,\alpha )=\rho _{\alpha ,s}$. Now consider an arbitrary leaf $x\in L(T(\rho _{\alpha ,s}))\cap L[s]{\setminus } N_s(\alpha )$. By construction we have ${{\,\mathrm{lca}\,}}(x,\alpha )\preceq \rho _{\alpha ,s}={{\,\mathrm{lca}\,}}(y,\alpha )$ and therefore $x\in N_s(\alpha )$. $\square$

We are now in the position to characterize the redundant edges.

### Lemma 15

Let $(G,\sigma )$ be a (not necessarily connected) *n*-cBMG explained by $(T,\sigma )$. Then the edge $e=uv$ is redundant in $(T,\sigma )$ if and only if (i) *e* is an inner edge of *T* and (ii) for every color $s\in \sigma (L(T(u){\setminus } T(v)))$, there is no  class $\alpha \in {\mathscr {N}}$ with $v=\rho _{\alpha ,s}$.

### Proof

Let $(T_e,\sigma )$ be the tree that is obtained from $(T,\sigma )$ by contraction of the edge $e=uv$ and assume that $(T_e,\sigma )$ explains $(G,\sigma )$. First we note that *e* is an inner edge and thus, in particular, $L(T_e)=L(T)$. Otherwise, i.e., if *e* is an outer edge, then $v\notin L(T_e)$; $(T_e,\sigma )$ does not explain $(G,\sigma )$. Now consider an inner edge *e*. Since $(T,\sigma )$ is phylogenetic, there exists a leaf $y\in L(T(u){\setminus } T(v))$ of some color $s\in \sigma (L(T(u){\setminus } T(v)))$. Assume that there is a  class $\alpha$ of *G* such that $v=\rho _{\alpha ,s}$. Note that $s\ne \sigma (\alpha )$ by definition of $\rho _{\alpha ,s}$. Lemma 14 implies that $y\notin N(\alpha )$ in $(G,\sigma )$. After contraction of *e*, we have ${{\,\mathrm{lca}\,}}(\alpha ,y)=\rho _{\alpha ,s}$, thus $y\in N(\alpha )$ by Lemma 14. Hence, $(T_e,\sigma )$ does not explain *G*; a contradiction.

Conversely, assume that *e* is an inner edge and for every $s\in \sigma (L(T(u){\setminus } T(v)))$, there is no $\alpha \in {\mathscr {N}}$ such that $v=\rho _{\alpha ,s}$, i.e., for every $\alpha \in {\mathscr {N}}$ and every color $s\ne \sigma (\alpha )$ we either have (i) $v\succ \rho _{\alpha ,s}$, (ii) $v\prec \rho _{\alpha ,s}$, or (iii) *v* and $\rho _{\alpha ,s}$ are incomparable. In the first two cases, contraction of *e* implies $v\succeq \rho _{\alpha ,s}$ or $v\preceq \rho _{\alpha ,s}$ in $(T_e,\sigma )$, respectively. Therefore, since $L(T(w))=L(T_e(w))$ for any *w* incomparable to *v*, we have $L(T(w))=L(T_e(w))$ for any node $w\ne v$. Moreover, it follows from Lemma 14 that $N_s(\alpha )=\{y\mid y\in L(T(\rho _{\alpha ,s})),\sigma (y)=s\}$. This implies that the set $N_s(\alpha )$ remains unchanged after contraction of *e* for all  classes $\alpha$ and all color $s\in S$. In other words, the in- and out-neighborhood of any leaf remain the same in $(T_e,\sigma )$. Hence, we conclude that $(T,\sigma )$ and $(T_e,\sigma )$ explain the same graph $(G,\sigma )$. $\square$

Before we consider the general case, we show that 3-cBMGs like 2-cBMGs are explained by unique least resolved trees.

### Lemma 16

Let $(G,\sigma )$ be a connected 3-cBMG. Then there exists a unique least resolved tree $(T,\sigma )$ that explains $(G,\sigma )$.

### Proof

This proof uses arguments very similar to those in the proof of uniqueness result for 2-cBMGs. In particular, as in the proof of Theorem 2, we assume for contradiction that there exist 3-colored digraphs that are explained by two distinct least resolved trees. Let $(G,\sigma )$ be a minimal graph (w.r.t. the number of vertices) that is explained by the two distinct least resolved trees $(T_1,\sigma )$ and $(T_2,\sigma )$. W.l.o.g. we can choose a vertex *v* and assume that its color is $r\in S$, i.e., $v\in L[r]$. Using the same notation as in the proof of Theorem 2, we write $(T'_1,\sigma ')$ and $(T'_2,\sigma ')$ for the trees that are obtained by deleting *v* from $(T,\sigma )$. These trees explain the uniquely defined graphs $(G'_1,\sigma ')$ and $(G'_2,\sigma ')$, respectively. Again, Lemma 1 implies that $(G',\sigma '):=(G[L{\setminus }\{v\}],\sigma ')$ is a subgraph of both $(G'_1,\sigma ')$ and $(G'_2,\sigma ')$. Similar to the case of 2-cBMGs, we characterize the additional edges that are inserted into $(G'_1,\sigma ')$ and $(G'_2,\sigma ')$ compared to $(G',\sigma ')$ in order to show that $(G'_1,\sigma ')=(G'_2,\sigma ')$. Assume that *uy* is an edge in $(G'_1,\sigma ')$ but not in $(G',\sigma ')$. By analogous arguments as in the proof of Theorem 2, we find that $uv\in E(G)$ and in particular $N_r(u)=\{v\}$, i.e., *u* has no out-neighbors of color *r* in $(G',\sigma ')$.

Moreover, we have $u\in L[s]$, where $s\in S{\setminus } \{r\}$. Similar to the 2-color case, we now determine the outgoing arcs of *u* in $(G'_1,\sigma ')$ and $(G'_2,\sigma ')$ by reconstructing the local structure of $(T_1,\sigma )$ and $(T_2,\sigma )$ in the vicinity of *v*.

Observation 1 implies that the least resolved tree $(T_{rs},\sigma _{rs})$ explaining $(G_{rs},\sigma _{rs})$ is displayed by both $(T_1,\sigma )$ and $(T_2,\sigma )$. The local structure of $(T_{rs},\sigma _{rs})$ around *v* is depicted in Fig. 5. Using the notation in the figure, $\{v\}$ is a  class by itself, $\alpha =\{v\}$, there is a  class $\beta '\subseteq L[s]$ with $N_r(\beta ')=\{\alpha \}$ and $N_s(\alpha )=\{\beta '\}$, and there may or may not exist a $\beta \subseteq L[s]$ with $N_r(\beta )=N_r(\beta ')=\{\alpha \}$ and $N_s(\alpha )\cap \beta '=\emptyset$. In addition, we have $\gamma \subseteq L[r]$, which is the $\preceq$-minimal  class of color *r* such that $\rho _\gamma \succ \rho _\beta , \rho _{\beta '}$. Recall that *uc* with $c\in \gamma$ are all the edges on $L[r]\times L[s]$ that have been additionally inserted in both $(G'_1,\sigma ')$ and $(G'_2,\sigma ')$. Since every  class has at least one out-neighbor of each color and given the relationship between $\alpha$ and $\beta '$, there exists a  class $\delta \subseteq L[t]$, where $t\in S{\setminus }\{r,s\}$, with $\alpha \subseteq N_r(\delta )$ and $\beta '\subseteq N_s(\delta )$ such that there is no other $\delta '\subseteq L[t]$ with $\rho _{\delta '}\prec \rho _\delta$. If $N_r(\delta ){\setminus } \{\alpha \}\ne \emptyset$, then $\rho _\delta \succeq \rho _\gamma$ by Lemma 14, and in particular there is no additional edge of the form *wa* with $w\in L[t]$ and $a\in L[r]$ that is contained in $(G'_1,\sigma ')$ and/or $(G'_2,\sigma ')$ but not in $(G',\sigma ')$. Therefore, only edges of the form *uc* with $c\in \gamma$ are additionally inserted into $(G'_1,\sigma ')$ and $(G'_2,\sigma ')$, and we conclude that $(G'_1,\sigma ')=(G'_2,\sigma ')$, which implies $(T'_1,\sigma ')=(T'_2,\sigma ')$ and therefore, since *v* was arbitrary, $(T_1,\sigma ')=(T_2,\sigma ')$; a contradiction.

Now consider the case $N_r(\delta ){\setminus } \{\alpha \}= \emptyset$. Since $\gamma \notin N_r(\delta )$, Lemma 14 ensures that $\rho _\delta \not \succeq \rho _\gamma$. The roots $\rho _{\gamma }$ and $\rho _{\delta }$ are comparable since $\alpha$ is an out-neighbor of both $\gamma$ and $\delta$. Thus $\rho _\delta \prec \rho _\gamma$ and hence $N_r(\delta )=\{\gamma \}$ in $(T'_1,\sigma ')$ as well as in $(T'_2,\sigma ')$ after deletion of *v*. We still need to distinguish two cases: either we have $N_s(\delta )=\{\beta '\}$ or $N_s(\delta )=\{\beta ',\beta \}$. In the first case, we have $\rho _\delta =\rho _{\beta '}=\rho _\alpha$ in $(T'_1,\sigma ')$ as well as in $(T'_2,\sigma ')$. In the second case, we obtain $\rho _\delta =\rho _{\beta }$, again this holds for both $(T'_1,\sigma ')$ and $(T'_2,\sigma ')$. As before, we can conclude that $(T'_1,\sigma ')=(T'_2,\sigma ')$ and therefore $(T_1,\sigma ')=(T_2,\sigma ')$; a contradiction. $\square$

If $(G,\sigma )$ is not connected, we can build a least resolved tree $(T,\sigma )$ analogously to the case of 2-cBMGs: we first construct the unique least resolved tree $(T_i,\sigma _i)$ for each component $(G_i,\sigma _i)$. Using Theorem 1 we then insert an additional root for $(T,\sigma )$ to which the roots of the $(G_i,\sigma _i)$ are attached as children. We proceed by showing that this construction corresponds to the unique least resolved tree.

### Theorem 7

Let $(G,\sigma )$ be a (not necessarily connected) *n*-cBMG with $n\in \{2,3\}$. Then there exists a unique least resolved tree $(T,\sigma )$ that explains $(G,\sigma )$.

### Proof

Denote by $(G_i,\sigma _i)$ the connected components of $(G,\sigma )$. By Theorem 2 and Lemma 16 there is a unique least resolved tree $(T_i,\sigma _i)$ that explains $(G_i,\sigma _i)$. Hence, if $(G,\sigma )$ is connected, we are done.

Now assume that there are at least two connected components. Let $(T,\sigma )$ be a least resolved tree that explains $(G,\sigma )$. Theorem 1 implies that there is a vertex $u\in \mathsf {child}(\rho _T)$ such that $L(G_i)\subseteq L(T(u))$ for each connected component $(G_i,\sigma _i)$. Hence, the subtree $(T(u),\sigma _{L(T(u))})$ displays the least resolved tree $(T_i,\sigma _i)$ explaining $(G_i,\sigma _i)$. Moreover, since $(T,\sigma )$ is least resolved, $\rho _Tu$ is a relevant edge, i.e., there must be a color $s\in \sigma (L(T{\setminus } T(u)))$ and a  class $\alpha$ such that $u=\rho _{\alpha ,s}$ by Lemma 15.

This implies in particular that there exists a leaf $x\in L(T(u))\cap L[s]$. Lemma 14 now implies that the elements of $\alpha$ are connected to any element of color *s* in the subtree $(T(u),\sigma _{L(T(u))})$. Furthermore, any leaf $y\in L(T(u))$ has at least one out-neighbor of color *s* in *L*(*T*(*u*)). Hence, we can conclude that the graph $G(T(u),\sigma _{L(T(u))})$ induced by the subtree $(T(u),\sigma _{L(T(u))})$ is connected.

Since $L(G_i)\subseteq L(T(u))$ and $(T(u),\sigma _{L(T(u))})$ explains the *maximal connected* subgraph $(G_i,\sigma _i)$, we conclude that $G(T(u),\sigma _{L(T(u))}) = (G_i,\sigma _i)$. By construction, both $(T(u),\sigma _{L(T(u))})$ and $(T_i,\sigma _i)$ are least resolved trees explaining the same graph, hence Theorem 2 and Lemma 16 imply $(T(u),\sigma _{L(T(u))})=(T_i,\sigma _i)$. In particular, thus, $\rho _{T_i}=u$.

As a consequence, any least resolved tree $(T,\sigma )$ that explains $(G,\sigma )$ must be composed of the disjoint trees $(T_i,\sigma _i)$ that are linked to the root via the relevant edge $\rho _T\rho _{T_i}$. Since every $(T_i,\sigma _i)$ and the construction of the edges $\rho _T\rho _{T_i}$ is unique, $(T,\sigma )$ is unique. $\square$

The characterization of redundant edges in trees explaining 2-cBMGs together with the uniqueness of the least resolved trees for 3-cBMGs can be used to characterize redundant edges in the general case, thereby establishing the existence of a unique least resolved tree for *n*-cBMGs.

### Theorem 8

For any connected *n*-cBMG $(G,\sigma )$, there exists a unique least resolved tree $(T',\sigma )$ that explains $(G,\sigma )$. The tree $(T',\sigma )$ is obtained by contraction of all redundant edges in an arbitrary tree $(T,\sigma )$ that explains $(G,\sigma )$. The set of all redundant edges in $(T,\sigma )$ is given by

$$\begin{aligned} {\mathfrak {E}}_T= \left\{ e=uv \mid v\notin L(T), v\ne \rho _{\alpha ,s} \text { for all } s\in \sigma (L(T(u){\setminus } T(v))) \text { and } \alpha \in {\mathscr {N}} \right\} . \end{aligned}$$

Moreover, $(T',\sigma )$ is displayed by $(T,\sigma )$.

### Proof

Using arguments analogous to the 2-color case one shows that there is a least resolved tree $(T',\sigma )$ that can be obtained from $(T,\sigma )$ by contraction of all redundant edges. The set of redundant edges is given by ${\mathfrak {E}}_T$ by Lemma 15. By construction, $(T',\sigma )$ is displayed by $(T,\sigma )$. It remains to show that $(T',\sigma )$ is unique. Observation 1 implies that for any pair of distinct colors *s* and *t* the corresponding unique least resolved tree $(T_{st},\sigma _{st})$ is displayed by $(T',\sigma )$. The same is true for the least resolved tree $(T_{rst},\sigma _{rst})$ for any three distinct colors $r,s,t\in S$. Since for any 2-cBMG as well as for any 3-cBMG, the corresponding least resolved tree is unique (see Theorem 2 and Lemma 16), it follows for any three distinct leaves $x,y,z\in L[r]\cup L[s]\cup L[t]$ that there is either a unique triple that is displayed by $(T_{rst},\sigma _{rst})$ or the least resolved tree $(T_{rst},\sigma _{rst})$ contains no triple on *x*, *y*, *z*. Note that we do not require that the colors *r*, *s*, *t* are pairwise distinct. Instead, we use the notation $(T_{rst},\sigma _{rst})$ to also include the trees explaining the induced 2-cBMGs. Observation 1 then implies that ${\mathcal {R}}^{*}:=\bigcup _{r,s,t \in S} r(T_{rst})\subseteq r(T')$. Now assume that there are two distinct least resolved trees $(T_1,\sigma )$ and $(T_2,\sigma )$ that explain $(G,\sigma )$. In the following we show that any triple displayed by $T_1$ must be displayed by $T_2$ and thus, $r(T_1)=r(T_2)$.

**Fig. 10:**
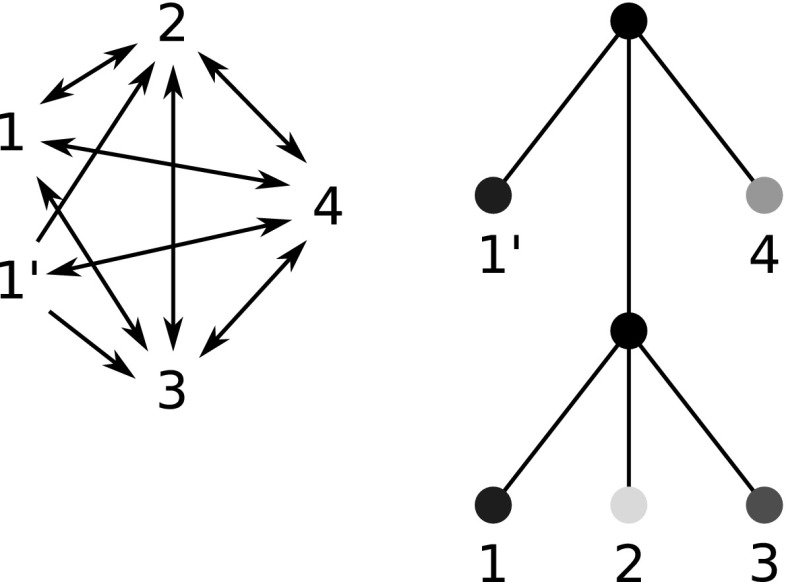
A connected graph $(G,\sigma )$ and the corresponding least resolved tree $(T,\sigma )$ on five vertices of four colors: blue (1 and 1’), yellow (2), red (3), and green (4). The triple 23|4 is displayed by $(T,\sigma )$ but it is not displayed by the least resolved tree $(T',\sigma ')$ that explains the induced subgraph $(G',\sigma ')$ with $V(G')=\{2,3,4\}$ since $(T',\sigma ')$ is simply the star tree on $\{2,3,4\}$. Hence, $23|4\notin {\mathcal {R}}^*=\bigcup _{r,s,t\in S}r(T_{rst})$ (color figure online)

Figure 10 shows that there may be triples $xy|z \in r(T_1){\setminus } {\mathcal {R}}^{*}$. Assume, for contradiction, that $xy|z \notin r(T_2){\setminus }{\mathcal {R}}^{*}$. Fix the notation such that $z\in \alpha$, $\sigma (x)=r$, $\sigma (y)=s$, and $\sigma (z)=t$. We do not assume here that *r*, *s*, *t* are necessarily pairwise distinct.

In the remainder of the proof, we will make frequent use of the following

**Observation:***If the treeTis a refinement of*$T'$, *then we have*$u\preceq _{T'} v$*if and only if*$u\preceq _T v$*for all*$u,v\in V(T')$.

In particular, $u\prec _{T'} v$ (i.e., $u\preceq _{T'} v$ and $u\ne v$) implies $u\prec _{T} v$. The converse of the latter statement is still true if *u* is a leaf in $T'$ but not necessarily for arbitrary inner vertices *u* and *v*.

Let $u={{\,\mathrm{lca}\,}}_{T_1}(x,y,z)$. The assumption $xy|z \in r(T_1)$ implies that there is a vertex $v\in \mathsf {child}(u)$ such that $v\succeq {{\,\mathrm{lca}\,}}_{T_1}(x,y)$. Since $(T_1,\sigma )$ is least resolved the characterization of relevant edges ensures that there is a color $p\in \sigma (L(T_1(u){\setminus } T_1(v)))$ and a  class $\beta$ with $\sigma (\beta )=q$ such that $v=\rho _{\beta ,p}$. In particular, there must be leaves $a\in L(T_1(v))$ and $a^*\in L(T_1(u){\setminus } T_1(v))$ with $\sigma (a)=\sigma (a^*)=p$. As a consequence we know that $a^* \notin N_p(b)$ for any $b\in \beta$.

We continue to show that the edge *uv* must also be contained in the least resolved tree $(T_{pq},\sigma _{pq})$ that explains the (not necessarily connected) graph $(G_{pq},\sigma _{pq})$. By Theorem 7, $(T_{pq},\sigma _{pq})$ is unique. Assume, for contradiction, that *uv* is not an edge in $T_{pq}$. Recalling the arguments in Observation 4, the tree $(T_1,\sigma )$ must display $(T_{pq},\sigma _{pq})$. Thus, if *uv* is not an edge in $T_{pq}$, then $v^*:=u=v$ in $T_{pq}$. By construction, we therefore have $v^*=\rho _{\beta ,p}$ in $(T_{pq},\sigma _{pq})$. Since $(T_{pq},\sigma _{pq})$ is least resolved, it follows from Corollary 3 that $b\in \mathsf {child}(v^*)$ for all $b\in \beta$ in $(T_{pq},\sigma _{pq})$. The latter, together with $a,a^*\preceq _{T_{pq}} v^*$, implies that ${{\,\mathrm{lca}\,}}_{T_{pq}}(a,\beta ) = {{\,\mathrm{lca}\,}}_{T_{pq}}(a^*,\beta )= v^*$. However, this implies $a^* \in N_p(\beta )$, a contradiction.

To summarize, the edge *uv* must be contained in the least resolved tree $(T_{pq},\sigma _{pq})$. Moreover, by Observation 4, $(T_{pqo},\sigma _{pqo})$ is a refinement of $(T_{pq},\sigma _{pq})$ for every color $o\in S$. Hence, we have $v\prec _{T_{pqo}} u$, which is in particular true for the color $o\in \{r,s,t\}$. Moreover, we know that $x \prec _{T_{pqr}} v$ and $y \prec _{T_{pqs}} v$ because $(T_1,\sigma )$ is a refinement of both $(T_{pqr},\sigma _{pqr})$ and $(T_{pqs},\sigma _{pqs})$.

Since $(T_2,\sigma )$ is also a refinement of both $(T_{pqr},\sigma _{pqr})$ and $(T_{pqs},\sigma _{pqs})$, we have $x,y\prec _{T_2} v \prec _{T_2} u$. Furthermore, $v \prec _{T_1} {{\,\mathrm{lca}\,}}_{T_1}(v,z)=u$ and $z \not \preceq _{T_1}$ implies that $z\prec _{T_{pqt}} u$ and $z \not \preceq _{T_{pqt}} v$. Therefore, $z \prec _{T_2} u$ and $z \not \preceq _{T_2} v$. Combining these facts about partial order of the vertices *v*, *u*, *x*, *y* and *z* in $T_2$, we obtain $xy|z\in r(T_2)$; a contradiction.

Hence, $r(T_1) = r(T_2)$. Since $r(T_1)$ uniquely identifies the structure of $T_1$ (cf. Semple and Steel 2003, Theorem 6.4.1), we conclude that $(T_1,\sigma )=(T_2,\sigma )$. The least resolved tree explaining $(G,\sigma )$ is therefore unique. $\square$

### Corollary 4

Every *n*-cBMG $(G,\sigma )$ is explained by the unique least resolved tree $(T,\sigma )$ consisting of the least resolved trees $(T_i,G_i)$ explaining the connected components $(G_i,\sigma _i)$ and an additional root $\rho _T$ to which the roots of the $(T_i,G_i)$ are attached as children.

### Proof

It is clear from the construction that $(T,\sigma )$ explains $(G,\sigma )$. The proof that his is the only least resolved tree parallels the arguments in the proof of Theorem 7 for 2-cBMGs and 3-cBMGs. $\square$

Since a tree is determined by all its triples, it is clear now that the construction of a tree that explains a connected *n*-cBMG is essentially a supertree problem: it suffices to find a tree, if it exists, that displays the least resolved trees explaining the induced subgraphs on 3 colors. In the following, we write

$$\begin{aligned} R:=\bigcup _{s,t \in S}r(T^*_{s,t}) \end{aligned}$$

for the union of all triples in the least resolved trees $(T^*_{st},\sigma _{st})$ explaining the 2-colored subgraphs $(G_{st},\sigma _{st})$ of $(G,\sigma )$. In contrast, the set of all *informative* triples of $(G,\sigma )$, as specified in Definition 8, is denoted by ${\mathcal {R}}(G,\sigma )$. As an immediate consequence of Lemma 12 we have

6 $$\begin{aligned} {\mathcal {R}}(G,\sigma )\subseteq R \end{aligned}$$

### Theorem 9

A connected colored digraph $(G,\sigma )$ is an *n*-cBMG if and only if (i) all induced subgraphs $(G_{st},\sigma _{st})$ on two colors are 2-cBMGs and (ii) the union *R* of all triples obtained from their least resolved trees $(T_{st},\sigma _{st})$ forms a consistent set. In particular, ${{\,\mathrm{Aho}\,}}(R)$ is the unique least resolved tree that explains $(G,\sigma )$.

### Proof

Let $(G,\sigma )$ be an *n*-cBMG that is explained by a tree $(T,\sigma )$. Moreover, let *s* and *t* be two distinct colors of *G* and let $L':= L[s]\cup L[t]$ be the subset of vertices with color *s* and *t*, respectively. Observation 1 states that the induced subgraph $(G[L'],\sigma )$ is a 2-cBMG that is explained by $(T_{L'},\sigma ')$. In particular, the least resolved tree $(T^*_{L'},\sigma ')$ of $(T_{L'},\sigma ')$ also explains $(G[L'],\sigma )$ and $T^*_{L'} \le T_{L'}\le T$ by Theorem 8, i.e., $r(T^*_{L'})\subseteq r(T)$. Since this holds for all pairs of two distinct colors, the union of the triples obtained from the set of all least resolved 2-cBMG trees *R* is displayed by *T*. In particular, therefore, *R* is consistent.

Conversely, suppose that $(G[L'],\sigma )$ is a 2-cBMG for any two distinct colors *s*, *t* and *R* is consistent. Let ${{\,\mathrm{Aho}\,}}(R)$ be the tree that is constructed by BUILD for the input set *R*. This tree displays *R* and is a least resolved tree (Aho et al. 1981) in the sense that we cannot contract any edge in ${{\,\mathrm{Aho}\,}}(R)$ without loosing a triple from *R*. By construction, any triple that is displayed by $(T_{st},\sigma _{st})$ is also displayed by ${{\,\mathrm{Aho}\,}}(R)$, i.e. $(T_{st},\sigma _{st})\le {{\,\mathrm{Aho}\,}}(R)$. Hence, for any $\alpha \in {\mathscr {N}}$ and any color $s\ne \sigma (\alpha )$ the out-neighborhood $N_s(\alpha )$ is the same w.r.t. $(T_{st},\sigma _{st})$ and w.r.t. ${{\,\mathrm{Aho}\,}}(R)$. Since this is true for any  class of *G*, also all in-neighborhoods are the same in ${{\,\mathrm{Aho}\,}}(R)$ and the corresponding $(T_{st},\sigma _{st})$. Therefore, we conclude that ${{\,\mathrm{Aho}\,}}(R)$ explains $(G,\sigma )$, i.e., $(G,\sigma )$ is an *n*-cBMG.

In order to see that ${{\,\mathrm{Aho}\,}}(R)$ is a least resolved tree explaining $(G,\sigma )$, we recall that the contraction of an edge leaves at least on triple unexplained, see Semple (2003, Prop. 4.1). Since *R* consists of all the triples $r(T_{st})$ that in turn uniquely identify the structure of $(T_{st},\sigma _{st})$ (cf. Semple and Steel 2003, Theorem 6.4.1), none of these triples is dispensable. The contraction of an edge in ${{\,\mathrm{Aho}\,}}(R)$ therefore yields a tree that no longer displays $(T_{st},\sigma _{st})$ for some pair of colors *s*, *t* and thus no longer explains $(G,\sigma )$. Thus, ${{\,\mathrm{Aho}\,}}(R)$ contains no redundant edges and we can apply Theorem 8 to conclude that ${{\,\mathrm{Aho}\,}}(R)$ is the unique least resolved tree that explains $(G,\sigma )$. $\square$

**Fig. 11:**
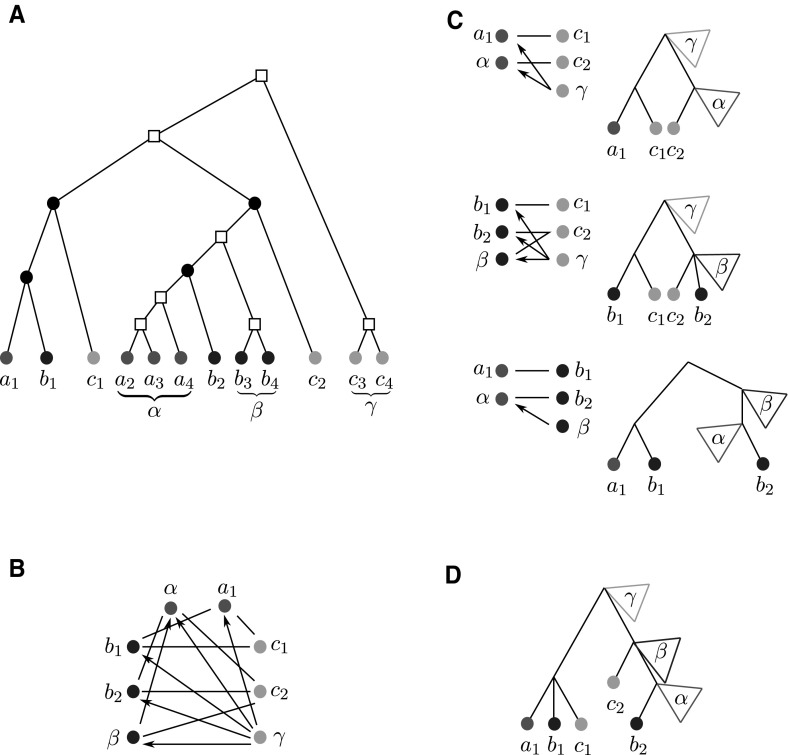
Construction of the least resolved tree explaining the colored best match graph. **a** Recalls the event-labeled gene tree of the evolutionary scenario shown in Fig. 1. There are three  classes with more than one element: $\alpha =\{a_2,a_3,a_4\}$, $\beta =\{b_3,b_4\}$ and $\gamma =\{c_3,c_4\}$ in the 3-cBMG graph $(G,\sigma )$ shown in (**b**). For simplicity of presentation, the  classes are already collapsed into single vertices. **c** Lists the three induced subgraphs of $(G,\sigma )$ on two colors together with their least resolved trees. By construction, $(G,\sigma )$ is the union of the three subgraphs on two colors. **d** The Aho-Tree for the set of all triples obtained from the least resolved trees shown in (**c**). This tree explains the graph $(G,\sigma )$ and is the unique least resolved tree w.r.t. $(G,\sigma )$

Figure 11 summarizes the construction of the least resolved tree from the 3-colored digraph $(G,\sigma )$ shown in Fig. 11b. For simplicity we assume that we already know that $(G,\sigma )$ is indeed a 3-cBMG. For each of the three colors the example has four genes. In addition to singleton there are three non-trivial  classes $\alpha =\{a_2,a_3,a_4\}$, $\beta =\{b_3,b_4\}$ and $\gamma =\{c_3$, $c_4\}$. Following Theorem 9, we extract for each of the three pairs of colors the induced subgraphs $(G_{st},\sigma _{st})$ and construct the least resolved trees that explain them (Fig. 11c). Extracting all triples from these least resolved trees on two colors yields the triple set ${\mathcal {R}}$, which in this case is consistent. Theorem 9 implies that the tree ${{\,\mathrm{Aho}\,}}({\mathcal {R}})$ (shown in the lower right corner) explains $(G,\sigma )$ and is in particular the unique least resolved tree w.r.t. $(G,\sigma )$.

We close this section by showing that in fact the informative triples of all $(G_{st},\sigma _{st})$ are already sufficient to decide whether $(G,\sigma )$ is an *n*-cBMG or not. More precisely, we show

### Lemma 17

If $(G,\sigma )$ is an *n*-cBMG then ${{\,\mathrm{Aho}\,}}({\mathcal {R}}(G,\sigma ))={{\,\mathrm{Aho}\,}}(R)$.

### Proof

We first observe that the two triple sets *R* and ${\mathcal {R}}:={\mathcal {R}}(G,\sigma )$ have the same Aho tree ${{\,\mathrm{Aho}\,}}(R) = {{\,\mathrm{Aho}\,}}({\mathcal {R}})$ if, in each step of BUILD, the respective Aho-graphs $[R,L']$ and $[{\mathcal {R}},L']$, as defined at the beginning of this section, have the same connected components. It is not necessary, however, that $[R,L']$ and $[{\mathcal {R}},L']$ are isomorphic. In the following set $T={{\,\mathrm{Aho}\,}}(R)$.

If *T* is the star tree on *L*, then ${\mathcal {R}}\subseteq R = \emptyset$, thus $[R,L]=[{\mathcal {R}},L]$ is the edgeless graph on *L*, hence in particular ${{\,\mathrm{Aho}\,}}({\mathcal {R}})={{\,\mathrm{Aho}\,}}(R)$.

Now suppose *T* is not the star tree. Then there is a vertex $w\in V^0(T)$ such that $L(T(w))=\mathsf {child}(w)$. For simplicity, we write $L_w:=L(T(w))$. Since $(T(w),\sigma _{L_w})$ is a star tree, we can apply the same argument again to conclude that $[R_{|L_w},L_w]=[{\mathcal {R}}_{|L_w},L_w]$, hence both Aho-graphs have the same connected components. Now let $u=\rho _T$ and assume by induction that $[R_{|L_{u'}},L_{u'}]$ and $[{\mathcal {R}}_{|L_{u'}},L_{u'}]$ have the same connected components for every $u'\prec _T u$, and thus, in particular, for $v\in \mathsf {child}(u)$. Consequently, for any $v_i\in \mathsf {child}(v)$ the set $L_{v_i}$ is connected in $[{\mathcal {R}}_{|L_{v}},L_{v}]$. Since ${\mathcal {R}}_{|L_{v}}\subseteq {\mathcal {R}}_{|L_{u}}$, the set $L_{v_i}$ must also be connected in $[{\mathcal {R}}_{|L_{u}},L_{u}]$ for every $v_i\in \mathsf {child}(v)$ (cf. Prop. 8 in Bryant and Steel 1995). It remains to show that all $L_{v_i}$ are connected in $[{\mathcal {R}}_{|L_{u}},L_{u}]$.

Since $(T,\sigma )$ is least resolved w.r.t. $(G,\sigma )$, it follows from Theorem 8 that $v=\rho _{\alpha ,s}$ for some color $s\in \sigma (L(T(u){\setminus } T(v)))$ and an  class $\alpha$ with $\sigma (\alpha )\ne s$. In particular, therefore, $s\notin \sigma (L_{v_i})$ if $\alpha \in L_{v_i}$ (say $i=1$). By definition of *s*, there must be a $v_j\in \mathsf {child}(v){\setminus } \{v_1\}$ (say $j=2$) such that $s\in \sigma (L_{v_2})$. Let $y\in L_{v_2}\cap L[s]$. Lemma 14 implies $y\in N_s(\alpha )$, i.e., $\alpha y \in E(G)$. Moreover, by definition of *s*, there must be a leaf $y'\in L(T(u){\setminus } T(v))\cap L[s]$. Since ${{\,\mathrm{lca}\,}}(\alpha ,y)\prec _T{{\,\mathrm{lca}\,}}(\alpha ,y')$, we have $\alpha y'\notin E(G)$, whereas $y'\alpha$ may or may not be contained in $(G,\sigma )$. Therefore, the induced subgraph on $\{\alpha y y'\}$ is of the form $X_1$, $X_2$, $X_3$, or $X_4$ and thus provides the informative triple $\alpha y | y'$. It follows that $L_{v_1}$ and $L_{v_2}$ are connected in $[{\mathcal {R}}_{|L_{u}},L_{u}]$. In particular, this implies that any $L_{v_j}$ with $\sigma (L_{v_j})\subseteq \sigma (L_v)$ containing *s* is connected to any $L_{v_i}$ that does not contain *s*. Since $(G,\sigma )$ is connected, such a set $L_{v_i}$ always exists by Theorem 1. Now let $L_1:=\{L_{v_j}\mid v_j\in \mathsf {child}(v), s\in \sigma (L_{v_j}) \}$ and $L_2:=\{L_{v_i}\mid v_i\in \mathsf {child}(v), s\notin \sigma (L_{v_i}) \}$. It then follows from the arguments above that $L_1$ and $L_2$ form a complete bipartite graph, hence $[{\mathcal {R}}_{|L_{u}},L_{u}]$ is connected. $\square$

As an immediate consequence, Theorem 9 can be rephrased as:

### Corollary 5

A connected colored digraph $(G,\sigma )$ is an *n*-cBMG if and only if (i) all induced subgraphs $(G_{st},\sigma _{st})$ on two colors are 2-cBMGs and (ii) the union ${\mathcal {R}}$ of informative triples ${\mathcal {R}}(G_{st},\sigma _{st})$ obtained from the induced subgraphs $(G_{st},\sigma _{st})$ forms a consistent set. In particular, ${{\,\mathrm{Aho}\,}}({\mathcal {R}})$ is the unique least resolved tree that explains $(G,\sigma )$.

## Algorithmic considerations

The material in the previous two sections can be translated into practical algorithms that decide for a given colored graph $(G,\sigma )$ whether it is an *n*-cBMG and, if this is the case, compute the unique least resolved tree that explains $(G,\sigma )$. The correctness of Algorithm 1 follows directly from Theorem 9 (for a single connected component) and Theorem 1 regarding the composition of connected components. It depends on the construction of the unique least resolved tree for the connected components of the induced 2-cBMGs, called LRTfrom2cBMG() in the pseudocode of Algorithm 1. There are two distinct ways of computing these trees: either by constructing the hierarchy $T({\mathcal {H}})$ from the extended reachable sets $R'$ (Algorithm 2) or via constructing the Aho tree from the set of informative triples (Algorithm 3). While the latter approach seems simpler, we shall see below that it is in general slightly less efficient. Furthermore, we use a function BuildST() to construct the supertree from a collection of input trees. Together with the computation of ${{\,\mathrm{Aho}\,}}()$ from a set of triples, it will be briefly discussed later in this section.
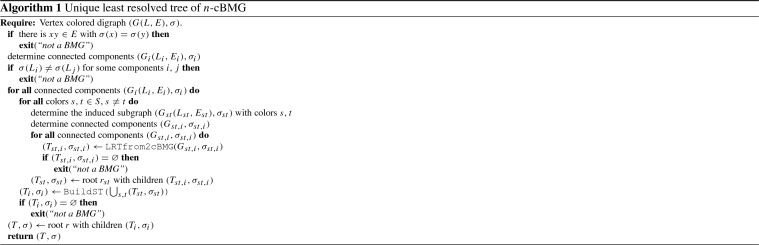

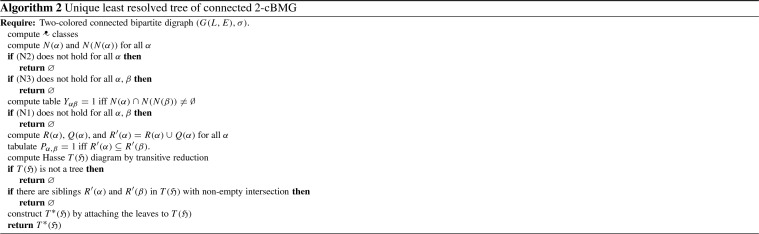

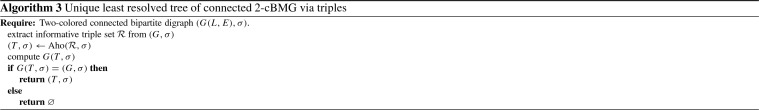


Let us now turn to analyzing the computational complexity of Algorithms 1, 2, and 3. We start with the building blocks necessary to process the 2-cBMG and consider performance bounds on individual tasks.

*From*$(T,\sigma )$*to*$(G,\sigma )$. Given a leaf-labeled tree $(T,\sigma )$ we first consider the construction of the corresponding cBMG. The necessary lowest common ancestor queries can be answered in constant time after linear time preprocessing, see e.g. (Harel and Tarjan 1984; Schieber and Vishkin 1988). The ${{\,\mathrm{lca}\,}}()$ function can also be used to express the partial orders among vertices since we have $x\preceq y$ if and only if ${{\,\mathrm{lca}\,}}(x,y)=y$. In particular, therefore, ${{\,\mathrm{lca}\,}}(x,y)\preceq {{\,\mathrm{lca}\,}}(x,y')$ is true if and only if ${{\,\mathrm{lca}\,}}({{\,\mathrm{lca}\,}}(x,y),{{\,\mathrm{lca}\,}}(x,y'))={{\,\mathrm{lca}\,}}({{\,\mathrm{lca}\,}}(x,y),y')={{\,\mathrm{lca}\,}}(x,y')$. Thus $(G,\sigma )$ can be constructed from $(T,\sigma )$ by computing ${{\,\mathrm{lca}\,}}(x,y)$ in constant time for each leaf *x* and each $y\in L[s]$. Since the last common ancestors for fixed *x* are comparable, their unique minimum can be determined in *O*(|*L*[*s*]|) time. Thus we can construct all best matches in $O( |L|+ |L|\sum _s |L|)=O(|L|^2)$ time.

*Thinness classes* Recall that each connected component of a cBMG $(G,\sigma )$ has vertices with all $|S|\ge 2$ colors (we disregard the trivial case of the edge-less graph with $|S|=1$) and thus every $x\in V$ has a non-zero out-degree. Therefore $|E|\ge |L|$, i.e., $O(|L|+|E|)=O(|E|){=} O(|L|^2)$.

Consider a collection ${\mathscr {F}}$ of $n=|{\mathscr {F}}|$ subsets on *L* with a total size of $m=\sum _{A\in {\mathscr {F}}}|A|$. Then the set inclusion poset of ${\mathscr {F}}$ can be computed in *O*(*nm*) time and $O(n^2)$ space as follows: For each $A\in {\mathscr {F}}$ run through all elements *x* of all other sets $B\in {\mathscr {F}}$ and mark $B\not \subseteq A$ if $x\notin A$, resulting in a $n\times n$ table $P_{{\mathscr {F}}}$ storing the set inclusion relation. More sophisticated algorithms that are slightly more efficient under particular circumstances are described in Pritchard (1995) and Elmasry (2010).

In order to compute the thinness classes, we observe that the symmetric part of $P_{{\mathscr {F}}}$ corresponds to equal sets. The classes of equal sets can be obtained as connected components by breadth first search on the symmetric part of $P_{{\mathscr {F}}}$ with an effort of $O(n^2)$. This procedure is separately applied to the in- and out-neighborhoods of the cBMG. Using an auxiliary graph in which $x,y\in L$ are connected if they are in the same component for both the in- and out- neighbors, the thinness classes can now be obtained by another breath first search in $O(n^2)$. Since we have $n=|L|$ and $m=|E|$ and thus the sets of vertices with equal in- and out-neighborhoods can be identified in $O(|L|\,|E|)$ total time.

*Recognizing 2-cBMGs* Since (N0) holds for all graphs, it will be useful to construct the table *X* with entries $X_{\alpha ,\beta }=1$ if $\alpha \subseteq N(\beta )$ and $X_{\alpha ,\beta }=0$ otherwise. This table can be constructed in *O*(|*E*|) time by iterating over all edges and retrieving (in constant time) the  classes to which its endpoints belong. The $N(N(\alpha ))$ can now be obtained in $O(|E|\,|L|)$ by iterating over all edges $\alpha \beta$ and adding the classes in $N(\beta )$ to $N(N(\alpha ))$. We store this information in a table with entries $Q_{\alpha ,\beta }=1$ if $\alpha \in N(N(\beta ))$ and $Q_{\alpha ,\beta }=0$ otherwise, in order to be able to decide membership in constant time later on.

A table $Y_{\alpha \beta }$ with $Y_{\alpha \beta }=0$ if $N(\alpha )\cap N(N(\beta ))=\emptyset$ and $Y_{\alpha \beta }=1$ if there is an overlap between $N(\alpha )$ and $N(N(\beta ))$ can be computed in $O(|L|^3)$ time from the membership tables *X* and *Q* for neighborhoods $N(\,.\,)$ and next-nearest neighborhoods $N(N(\,.\,))$, respectively. From the membership table for $N(N(\alpha ))$ and $N(\gamma )$ we obtain $N(N(N(\alpha )))$ in $O(|E|\,|L|)$ time, making use of the fact that $\sum _{\alpha }|N(\alpha )|=|E|$. For fixed $\alpha ,\beta \in {\mathscr {N}}$ it only takes constant time to check the conditions in (N1) and (N3) since all set inclusions and intersections can be tested in constant time using the auxiliary data derived above. The inclusion (N2) can be tested directly in *O*(|*L*|) time for each $\alpha$. We can summarize considerations above as

### Lemma 18

A 2-cBMG can be recognized in $O(|L|^2)$ space and $O(|L|^3)$ time with Algorithm 2.

*Reconstruction of*$T^*({\mathcal {H}})$. For each $\alpha \in {\mathscr {N}}$, the reachable set $R(\alpha )$ can be found by a breadth first search in *O*(|*E*|) time, and hence with total complexity $O(|E|\,|L|)$. For each $\alpha$, we can find all $\beta \in {\mathscr {N}}$ with $N^-(\beta )=N^-(\alpha )$ and $N(\beta )\subseteq N(\alpha )$ in *O*(|*L*|) time by simple look-ups in the set inclusion table for the in- and out-neighborhoods, respectively. Thus we can find all auxiliary leaf sets $Q(\alpha )$ in $O(|L^2|)$ time and the collection of the $R'(\alpha )$ can be constructed in $O(|E|\,|L|)$.

The construction of the set inclusion poset is also useful to check whether the $\{R'(\alpha )\}$ form a hierarchy. In the worst case we have a tree of depth |*L*| and thus $m=O(|L|^2)$. Since the number of  classes is bounded by *O*(|*L*|), the inclusion poset of the reachable sets can be constructed in $O(|L|^3)$. The Hasse diagram of the partial order is the unique transitive reduction of the corresponding digraph. In our setting, this also takes $O(|L|^3)$ time (Gries et al. 1989; Aho et al. 1972), since the inclusion poset of the $\{R'(\alpha )\}$ may have $O(|L|^2)$ edges. It is now easy to check whether the Hasse diagram is a tree or not. If the number of edges is at least the number of vertices, the answer is negative. Otherwise, the presence of a cycle can be verified e.g. using breadth first search in *O*(|*L*|) time. It remains to check that the non-nested sets $R(\alpha )$ are indeed disjoint. It suffices to check this for the children of each vertex in the Hasse tree. Traversing the tree top-down this can be verified in $O(|L|^2)$ time since there are *O*(|*L*|) vertices in the Hasse diagram and the total number of elements in the subtrees is *O*(|*L*|).

Summarizing the discussion so far, and using the fact that the vertices $x\in \alpha$ can be attached to the corresponding vertices $R'(\alpha )$ in total time *O*(|*L*|) we obtain

### Lemma 19

The unique least resolved tree $T^*({\mathcal {H}}')$ of a connected 2-cBMG $(G,\sigma )$ can be constructed in $O(|L|^3)$ time and $O(|L|^2)$ space with Algorithm 2.

*Informative triples* Since all informative triples ${\mathcal {R}}(G,\sigma )$ come from an induced subgraph that contains at least one edge, it is possible to extract ${\mathcal {R}}(G,\sigma )$ for a connected 2-cBMG in $O(|E|\,|L|)$ time. Furthermore, the total number of vertices and edges in ${\mathcal {R}}(G,\sigma )$ is also bounded by $O(|E|\,|L|)$, hence the algorithm of Deng and Fernández-Baca can be used to construct the tree ${{\,\mathrm{Aho}\,}}({\mathcal {R}}(G,\sigma ))$ for a connected 2-cBMG in $O(|E|\,|L| \log ^2(|E|\,|L|) )$ time (Deng and Fernández-Baca 2018). The graph $(G',\sigma )$ explained by this tree can be generated in $O(|L|^3)$ time, and checking whether $(G,\sigma )=(G',\sigma )$ requires $O(|L|^2)$ time. Asymptotically, the approach via informative triples, Algorithm 3, is therefore at best as good as the direct construction of the least resolved tree $T^*({\mathcal {H}}')$ with Algorithm 2.

*Effort in then-color case* For *n*-cBMGs it is first of all necessary to check all pairs of induced 2-cBMGs. The total effort for processing all induced 2-cBMGs is $O(\sum _{s<t} (|L[s]|+|L[t]|)^3) \le O(|S|\,|L|\,\ell ^2 + |L|^2\ell )$ with $\ell :=\max _{s\in S} |L[s]|$, as shown by a short computation.

The 2-cBMG for colors *s* and *t* is of size $O(L[s]+L[t])$ hence the total size of all $|S|(|S|-1)/2$ 2-cBMGs is $O(|S|\,|L|)$. The total effort to construct a supertree from these 2-cBMGs is therefore only $O(|L|\,|S| \log ^2(|L|\,|S|))$ (Deng and Fernández-Baca 2018), and thus negligible compared to the effort of building the 2-cBMGs.

Using Lemma 5 it is also possible to use the set of all informative triples directly. Its size is bounded by $O(|L|\,|E|)$, hence the algorithm of Rauch Henzinger et al. (1999) can used to construct the supertree on $O( |L|\,|E|\log ^2(|L|\,|E| )$. This bound is in fact worse than for the strategy of constructing all 2-cBMGs first.

We note, finally, that for practical applications the number of genes between different species will be comparable, hence $O(\ell )=O(|L|/|S|)$. The total effort of recognizing an *n*-cBMG in a biologically realistic application scenario amounts to $O(|L|^3/|S|)$. In the worst case scenario with $O(\ell )=O(|L|)$, the total effort is $O(|S|\, |L|^3)$.

## Reciprocal best match graphs

**Fig. 12:**
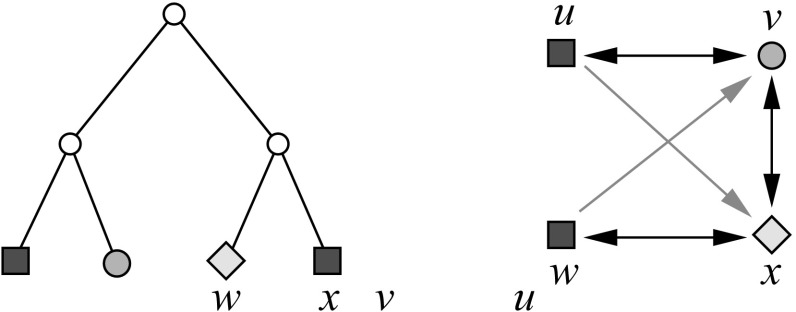
Colored Reciprocal Best Match Graphs are not necessarily cographs. This simple counterexample contains the path $u-v-x-w$ as symmetric part. It corresponds to a species tree of the form  and a duplication pre-dating the two speciations, with the speciation of  and  being followed by complementary loss of one of the two copies

Several software tools implementing methods for tree-free orthology assignment are typically on reciprocal best matches, i.e., the symmetric part of a cBMG, which we will refer to as *colored Reciprocal Best Match Graph* (cRBMG). Orthology is well known to have a cograph structure (Hellmuth et al. 2013; Hellmuth and Wieseke 2018, 2016). The example in Fig. 12 shows, however, that cRBMG in general are not cographs. It is of interest, therefore to better understand this class of colored graphs and their relationships with cographs.

### Definition 10

A vertex-colored undirected graph $G(V,E,\sigma )$ with $\sigma :V\rightarrow S$ is a *colored reciprocal best match graph* (cRBMG) if there is a tree *T* with leaf set *V* such that $xy\in E$ if and only if ${{\,\mathrm{lca}\,}}(x,y)\preceq {{\,\mathrm{lca}\,}}(x,y')$ for all $y'\in V$ with $\sigma (y')=\sigma (y)$ and ${{\,\mathrm{lca}\,}}(x,y)\preceq {{\,\mathrm{lca}\,}}(x',y)$ for all $x'\in V$ with $\sigma (x')=\sigma (x)$.

By definition $G(V,E,\sigma )$ is a cRBMG if and only if there is a cBMG $(G',\sigma )$ with vertex set *V* and edges $xy\in E(G)$ if and only if both (*x*, *y*) and (*y*, *x*) are arcs in $(G',\sigma )$. In particular, therefore, a cRBMG is the edge-disjoint union of the edge sets of the induced cRBMGs by pairs of distinct colors $s,t\in S$.

**Fig. 13:**
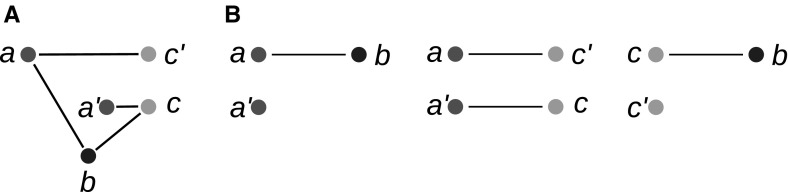
**a** A symmetric graph on three colors. **b** Each induced subgraph on two colors is a reciprocal Best Match Graph and a disjoint union of complete bipartite graphs. However, the corresponding symmetric graph on three colors shown in (**a**) does not have a tree representation

### Corollary 6

Every 2-cRBMG is the disjoint union of complete bipartite graphs.

### Proof

By Lemma 4 there are arcs (*x*, *y*) and (*y*, *x*) if and only if $x\in \alpha \subseteq N(\beta )$ and $y\in \beta \subseteq N(\alpha )$. In this case $\rho _{\alpha }=\rho _{\beta }$. By Lemma 3(v) then $\sigma (\alpha )\ne \sigma (\beta )$. The same results also implies in a 2-cRBMG there are at most two  classes with the same root. Thus the connected components of a 2-cRBMG are the complete bipartite graphs formed by pairs of  classes with a common root, as well as isolated vertices corresponding to all other leaves of *T*. $\square$

The converse, however, is not true, as shown by the counterexample in Figure 13. The complete characterization of cRBMGs does not seem to follow in a straightforward manner from the properties of the underlying cBMGs. It will therefore be addressed elsewhere.

## Concluding remarks

The main result of this contribution is a complete characterization of colored best match graphs (cBMGs), a class of digraphs that arises naturally at the first stage of many of the widely used computational methods for orthology assignment. A cBMG $(G,\sigma )$ is explained by a unique least resolved tree $(T,\sigma )$, which is displayed by the true underlying tree. We have shown here that cBMGs can be recognized in cubic time (in the number of genes) and with the same complexity it is possible to reconstruct the unique least resolved tree $(T,\sigma )$. Related graph classes, for instance directed cographs (Crespelle and Paul 2006), which appear in generalizations of orthology relations (Hellmuth et al. 2017), or the Fitch graphs associated with horizontal gene transfer (Geiß et al. 2018), have characterizations in terms of forbidden induced subgraphs. We suspect that this not the case for best match graphs because they are not hereditary.

Reciprocal best match graphs, i.e., the symmetric subgraph of $(G,\sigma )$, form the link between cBMGs and orthology relations. The characterization of cRBMGs, somewhat surprisingly, does not seem to be a simple consequence of the results on cBMGs presented here. We will address this issue in future work.

Several other questions seem to be appealing for future work. Most importantly, what if the vertex coloring is not known *a priori*? What are the properties of BMGs in general? For connected 2-cBMGs the question is simple, since the bipartition is easily found by a breadth first search. In general, however, we suspect that—similar to many other coloring problems—it is difficult to decide whether a digraph *G* admits a coloring $\sigma$ with $n=|S|$ colors such that $(G,\sigma )$ is an *n*-cBMG. In the same vein, we may ask for the smallest number *n* of colors, if it exists, such that *G* can be colored as an *n*-cBMG.

As discussed in the introduction, usually sequence similarities are computed. In the presence of large differences in evolutionary rates between paralogous groups, maximal sequence similarity does not guarantee maximal evolutionary relatedness. It is often possible, however, to identify such problematic cases. Suppose the three species *a*, *b*, and *c* form a triple *ab*|*c* that is trustworthy due to independent phylogenetic information. Now consider a gene *x* in *a*, two candidate best matches $y'$ and $y''$ in *b*, and a candidate best match *z* in *c*. To decide whether ${{\,\mathrm{lca}\,}}(x,y')\prec {{\,\mathrm{lca}\,}}(x,y'')$ or not, we can use the support for the three possible unrooted quadruples formed by the sequences $\{x,y',y'',z\}$ to decide whether ${{\,\mathrm{lca}\,}}(x,y')\prec {{\,\mathrm{lca}\,}}(x,y'')$, which can be readily computed as the likelihoods of the three quadruples or using quartet-mapping (Nieselt-Struwe 2001). If the best supported quadruples is $(xy'|y''z)$ or $(xy''|y'z)$ it is very likely that ${{\,\mathrm{lca}\,}}(x,y')\prec {{\,\mathrm{lca}\,}}(x,y'')$ or ${{\,\mathrm{lca}\,}}(x,y'')\prec {{\,\mathrm{lca}\,}}(x,y')$, respectively, while $(xz|y'y'')$ typically indicates ${{\,\mathrm{lca}\,}}(x,y'')={{\,\mathrm{lca}\,}}(x,y')$. This inference is correct as long a *z* is correctly identified as outgroup to $x,y',y''$, which is very likely since all three of $y',y'',z$ are candidate best matches of *x* in the first place. Aggregating evidence over different choices of *z* thus could be used to increase the confidence. An empirical evaluation of this approach to improve blast-based best hit data is the subject of ongoing research.

From a data analysis point of view, finally, it is of interest to ask whether an *n*-colored digraph $(G,\sigma )$ that is not a cBMG can be edited by adding and removing arcs to an *n*-cBMG. This idea has been used successfully to obtain orthologs from noisy, empirical reciprocal best hit data, see e.g. Hellmuth et al. (2013), Lafond and El-Mabrouk (2014), Hellmuth et al. (2015) and Lafond et al. (2016); Dondi et al. (2017). We propose that a step-wise approach could further improve the accuracy of orthology detection. In the first step, empirical (reciprocal) best hit data obtained with ProteinOrtho or a similar tool would be edited to conform to a cBMG or a cRBMG. These improved data are edited in a second step to the co-graph structure of an orthology relation. Details on cRBMGs and their connections with orthology will be discussed in forthcoming work.
