# Corrigendum to “Best match graphs”

**DOI:** 10.1007/s00285-021-01601-6

**Published:** 2021-04-05

**Authors:** David Schaller, Manuela Geiß, Edgar Chávez, Marcos González Laffitte, Alitzel López Sánchez, Bärbel M. R. Stadler, Dulce I. Valdivia, Marc Hellmuth, Maribel Hernández Rosales, Peter F. Stadler

**Affiliations:** 1 Max-Planck-Institute for Mathematics in the Sciences, Inselstraße 22, 04103 Leipzig, Germany; 2 Software Competence Center Hagenberg GmbH, Softwarepark 21, 4232 Hagenberg, Austria; 3 Centro de Física Aplicada y Tecnología Avanzada, UNAM, 76230 Juriquilla, QRO México; 4 Instituto de Matemáticas, UNAM Juriquilla, Blvd. Juriquilla 3001, 76230 Juriquilla, Querétaro, QRO México; 5 Department of Computer Science, Université de Sherbrooke, 2500 Boul. de l’Université, Sherbrooke, J1K 2R1 Canada; 6 Centro de Investigación y de Estudios Avanzandos del IPN (CINVESTAV), Irapuato Unit, Libramiento Norte, Carretera Panamericana Irapuato-León Kilómetro 9.6, 36821 Irapuato, Guanajuato México; 7 Department of Mathematics, Faculty of Science, Stockholm University, SE - 106 91 Stockholm, Sweden; 8 Bioinformatics Group, Department of Computer Science, Interdisciplinary Center of Bioinformatics, German Centre for Integrative Biodiversity Research (iDiv) Halle-Jena-Leipzig, Competence Center for Scalable Data Services and Solutions, Leipzig, Germany; 9 Leipzig Research Center for Civilization Diseases, Leipzig University, Härtelstraße 16-18, 04107 Leipzig, Germany; 10 Institute for Theoretical Chemistry, University of Vienna, Währingerstraße 17, 1090 Wien, Austria; 11 Facultad de Ciencias, Universidad National de Colombia, Sede Bogotá, Colombia; 12 Santa Fe Institute, 1399 Hyde Park Rd., Santa Fe, NM 87501 USA

**Keywords:** Corrigendum, Phylogenetic Combinatorics, Colored digraph, Rooted triples

## Abstract

Two errors in the article *Best Match Graphs* (Geiß et al. in JMB 78: 2015–2057, 2019) are corrected. One concerns the tacit assumption that digraphs are sink-free, which has to be added as an additional precondition in Lemma 9, Lemma 11, Theorem 4. Correspondingly, Algorithm 2 requires that its input is sink-free. The second correction concerns an additional necessary condition in Theorem 9 required to characterize best match graphs. The amended results simplify the construction of least resolved trees for *n*-cBMGs, i.e., Algorithm 1. All other results remain unchanged and are correct as stated.

## Best match graphs (BMGs) must be sink-free

Throughout Geiß et al. (2019) we have tacitly assumed that all vertex-colored digraphs $$(G,\sigma
							)$$ satisfy the following property, which, by construction, is true for all colored best match graphs (cBMGs):

- *For each vertex* *x with color* $$\sigma
										(x)$$,* there is an arc* *xy* *to at least one vertex of* *y* *every other color* $$\sigma
										(y)\ne \sigma (x)$$.

All properly 2-colored digraphs appearing in the text are therefore assumed to be sink-free, i.e., the out-neighborhoods of their 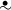-classes are assumed to be non-empty:

(N4) $$N(\alpha
										)\ne \emptyset $$ for all $$\alpha
										\in {\mathcal {N}}$$.

This assumption was not clearly stated in the text.

Property (N4) is required in the proof of Lemma 9 [last line on page 2032]: Here $$R(\beta )\cap
							R(\alpha ^*)=\emptyset $$ only implies $$\beta \subseteq R(\alpha
							)$$ for the 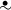 classes $$\beta
							$$ with $$R(\beta )\subseteq R(\alpha
							)$$ if $$R(\beta )\ne \emptyset
							$$, which in turn is equivalent to $$N(\beta )\ne
							\emptyset $$. Furthermore, $$N(\alpha )=\emptyset
							$$ implies $$Q(\alpha )=\alpha $$ and thus $$R'(\alpha )=\alpha
							$$. Property (N4) is therefore also necessary to ensure that $$|R'(\alpha
							)|>1$$ and thus that the tree $$T({\mathcal {H}}')$$ is phylogenetic [page 2035, just before Theorem 4]. In summary, Lemma 9, Lemma 11, and Theorem 4 require (N4) as additional precondition.

## Corrected characterization of *n*-cBMGs

The second paragraph of the proof of Theorem 9 in (Geiß et al. 2019, page 2045) incorrectly states that “*for any* $$\alpha \in {\mathcal
							{N}}$$*and any color* $$s\ne \sigma (\alpha
							)$$ *the out-neighborhood* $$N_s(\alpha
							)$$ *is the same w.r.t.* $$(T_{st},\sigma
							_{st})$$ *and w.r.t.* $${{\,\mathrm{Aho}\,}}(R)$$.”, leading to the incorrect conclusion that $$G({{\,\mathrm{Aho}\,}}(R),\sigma )=(G,\sigma
							)$$ whenever $${{\,\mathrm{Aho}\,}}(R)$$ exists. We recall that the triple set *R* is defined as the union

$$\begin{aligned} R {:}{=}\bigcup _{s,t\in S}
							r(T^*_{st}) \end{aligned}$$

of all triples in the least resolved trees $$(T^*_{st},\sigma
							_{st})$$ that explain the induced subgraphs $$(G_{st},\sigma
							_{st})$$ of $$(G,\sigma )$$, and the Aho tree $${{\,\mathrm{Aho}\,}}(R)$$ is defined on the leaf set $$L=V(G)$$ (which may not have been clear from the wording in the text). We shall show in Proposition 3 below that $$G({{\,\mathrm{Aho}\,}}(R),\sigma
							)$$ is always a subgraph of $$(G,\sigma )$$ whenever *R* is consistent. The example in Fig. 1 shows, however, that $$G({{\,\mathrm{Aho}\,}}(R),\sigma )\ne (G,\sigma
							)$$ is possible because $${{\,\mathrm{Aho}\,}}(R)$$ can contain triples that are not present in any of the 2-colored trees $$(T_{st},\sigma
							_{st})$$.

**Fig. 1:**
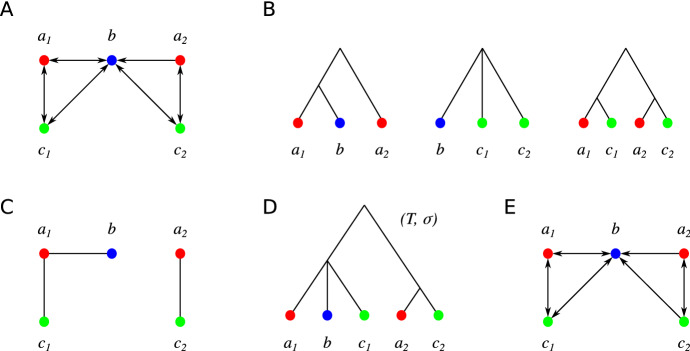
Counterexample for the original version of Theorem 9. **a** A colored digraph with vertex set *L* that is not a 3-cBMG. **b** The least resolved subtrees for the three 2-colored induced subgraphs. The union of their triples is $$R{:}{=}\{a_1b|a_2,\; a_1c_1|a_2,\;
										a_1c_1|c_2,\; a_2c_2|a_1,\;
										a_2c_2|c_1\}$$. **c** The Aho-graph [*R*, *L*]. In particular, *R* forms a consistent set. **d** The tree $$T{:}{=}{{\,\mathrm{Aho}\,}}(R)$$. **e** The 3-cBMG $$G(T,\sigma
										)$$. The arc $$bc_2$$ that was present in $$(G,\sigma
										)$$ is missing in $$G(T,\sigma
										)$$

As a consequence, the characterization of *n*-cBMGs requires the equality $$(G,\sigma
							)=G({{\,\mathrm{Aho}\,}}(R),\sigma )$$ as an additional condition. The corrected result, with the correction underlined, reads as follows:

### Theorem 9

A connected colored digraph $$(G,\sigma )$$ is an *n*-cBMG if and only if (1) all induced subgraphs $$(G_{st},\sigma
								_{st})$$ on two colors are 2-cBMGs, (2) the union *R* of all triples obtained from their least resolved trees $$(T_{st},\sigma
								_{st})$$ forms a consistent set, and $$\underline{
								\textit{(3)}\, G({{\,\mathrm{Aho}\,}}(R),\sigma )=(G,\sigma
								)}$$. In particular, $$({{\,\mathrm{Aho}\,}}(R),\sigma
								)$$ is the unique least resolved tree that explains $$(G,\sigma
								)$$.

Condition (1) in Theroem 9, i.e., the requirement that all 2-colored induced subgraphs $$(G_{st},\sigma
								_{st})$$ of $$(G,\sigma )$$ are 2-cBMGs, is necessary to ensure that the least resolved trees $$(T_{st},\sigma
								_{st})$$ exist and thus that the triple sets $$r(T_{st})$$ – and therefore also the set *R* of all triples displayed by the 2-colored induced subgraphs – are well-defined. Consistency of *R* is necessary for the existence of $${{\,\mathrm{Aho}\,}}(R)$$. Clearly, Condition (3) is sufficient to ensure that $$(G,\sigma
								)$$ is an *n*-cBMG. Hence, it remains to show that Condition (3) is also necessary. This is achieved in Proposition 2 below.

## Proof of theorem 9

Instead of proving the corrected version of Theorem 9 directly, we first state and prove a slightly stronger and more convenient result, Theorem 1 below, and then proceed to derive Theroem 9. To this end, we first generalize Definition 8 in (Geiß et al. (2019), page 2036) to digraphs with an arbitrary number of colors:

### Definition 1

(Schaller et al. (2021), Definition 2.7) Let $$(G,\sigma )$$ be a colored digraph. We say that a triple $$ab|b'$$ is *informative* for $$(G,\sigma
								)$$ if *a*, *b* and $$b'$$ are pairwise distinct vertices in $$G$$ such that (i) $$\sigma (a)\ne \sigma (b)=\sigma
								(b')$$ and (ii) $$ab\in E(G)$$ and $$ab'\notin E(G)$$. The set of informative triples is denoted by $${\mathcal
								{R}}(G,\sigma )$$.

We briefly argue that, for 2-colored digraphs, the definition of informative triples given here is equivalent to the one given in Geiß et al. (2019): By definition, an informative triple of some colored digraph has vertices with exactly two colors, and thus is also an informative triple in one of its 2-colored induced subgraphs. It is easy to check that, for 2-colored digraphs, Definition 1 is equivalent to Definition 8 in Geiß et al. (2019), since the four induced subgraphs shown in Fig. 8 in (Geiß et al. (2019), page 2036) correspond to the presence or absence of the two optional arcs *ba* and *ca* in the informative triple *ab*|*c* (as defined here).

We will also make use of a generalization of Lemma 12 in (Geiß et al. (2019), page 2036):

### Lemma 1

(Schaller et al. (2021), Lemma 2.8) Let $$(G,\sigma )$$ be an *n*-cBMG and $$ab|b'$$ an informative triple for $$(G,\sigma )$$. Then, every tree $$(T,\sigma )$$ that explains $$(G,\sigma )$$ displays the triple $$ab|b'$$, i.e. $${{\,\mathrm{lca}\,}}_T(a,b)\prec
								_T{{\,\mathrm{lca}\,}}_T(a,b')={{\,\mathrm{lca}\,}}_T(b,b')$$.

Given a digraph $$(G,\sigma
								)$$ for which *R* exists, Lemma 1 in particular implies that

1 $$\begin{aligned}
								{\mathcal {R}}(G,\sigma )\subseteq R.
								\end{aligned}$$

With these preliminaries, we are ready to formulate our new main result as

### Theorem 1

A colored digraph $$(G,\sigma
								)$$ is an *n*-cBMG if and only if $$G({{\,\mathrm{Aho}\,}}({\mathcal {R}}(G,\sigma
								)),\sigma ) = (G,\sigma )$$. Moreover, $${{\,\mathrm{Aho}\,}}({\mathcal {R}}(G,\sigma
								))$$ is the unique least resolved tree explaining an n-cBMG $$(G,\sigma
								)$$.

In order to prove Theorem 1, we will first provide several technical results that make use of the notion of (non-)redundant tree edges and, in particular, of least resolved trees. Recall that an inner edge *e* in a leaf-colored tree $$(T,\sigma )$$ is *redundant* if the tree $$(T_e,\sigma
								)$$ obtained from *T* by contraction of *e* explains the same *n*-cBMG, i.e., if $$G(T,\sigma
								)=G(T_e,\sigma )$$. A tree $$(T,\sigma )$$ is called *least resolved* if it does not contain any redundant edges. We will need the following, simplified, characterization of redundant edges:

### Lemma 2

(Schaller et al. (2021), Lemma 2.10) Let $$(G,\sigma )$$ be an *n*-cBMG explained by a tree $$(T,\sigma
								)$$. The edge $$e=uv$$ with $$v\prec _T u$$ in $$(T,\sigma )$$ is redundant w.r.t. $$(G,\sigma )$$ if and only if (i) *e* is an inner edge of *T* and (ii) there is no arc $$ab\in E(G)$$ such that $${{\,\mathrm{lca}\,}}_T(a,b)=v$$ and $$\sigma (b)\in \sigma (L(T(u))\setminus
								L(T(v)))$$.

We note that the proofs of Lemma 1 (Schaller et al. (2021), Lemma 2.8) and Lemma 2 (Schaller et al. (2021), Lemma 2.10) only require the definition of best match graphs, and are thus independent of the results proved in Geiß et al. (2019).

Following Bryant and Steel (1995), an inner edge *e* of a rooted tree *T* is *distinguished* by a triple $$ab|c\in r(T)$$ if the path from *a* to *c* in *T* intersects the path from *b* to the root $$\rho _T$$ precisely on the edge e. In other words, $$e=uv$$ with $$v\prec _T u$$ is distinguished by *ab*|*c* if $${{\,\mathrm{lca}\,}}_T(a, b) =
								v$$ and $${{\,\mathrm{lca}\,}}_T(a, b, c) =
								u$$. Lemma 2 immediately implies the following generalization of Lemma 13 in Geiß et al. (2019):

### Corollary 1

Let $$(G,\sigma
								)$$ be an *n*-cBMG explained by a tree $$(T,\sigma
								)$$. An inner edge *e* of $$(T,\sigma
								)$$ is non-redundant w.r.t. $$(G,\sigma )$$ if and only if it is distinguished by an informative triple $$ab|b'$$ for $$(G,\sigma )$$. In particular, if $$(T,\sigma )$$ is least resolved, then each of its inner edges is distinguished by an informative triple.

In addition, we will need the following two technical results relating subtrees and induced subgraphs of *n*-cBMGs.

### Lemma 3

Let $$(T,\sigma
								)$$ be a tree explaining an *n*-cBMG $$(G,\sigma
								)$$. Then $$G(T(u),\sigma _{|L(T(u))}) = (G[L(T(u))],\sigma
								_{|L(T(u))})$$ holds for every $$u\in V(T)$$.

### Proof

Let $$(G_1, \sigma
								'){:}{=}G\left( T(u), \sigma _{|L(T(u))}\right)
								$$ and $$(G_2, \sigma '){:}{=}(G[L(T(u))],\sigma
								_{|L(T(u))})$$. By definition, we have $$V(G_1)=V(G_2)=L(T(u))$$. First assume that $$xy\in E(G_1)$$ for some $$x,y\in L(T(u))$$. Hence, it holds that $${{\,\mathrm{lca}\,}}_{T(u)}(x,y)\preceq
								_{T(u)}{{\,\mathrm{lca}\,}}_{T(u)}(x,y')$$ for all $$y'$$ with $$\sigma (y)=\sigma
								(y')$$ in *T*(*u*) and thus, since *T*(*u*) is a subtree of *T*, we have $${{\,\mathrm{lca}\,}}_T(x,y)\preceq
								_T{{\,\mathrm{lca}\,}}_T(x,y')$$ for all $$y'$$ with $$\sigma (y)=\sigma
								(y')$$ in *T*. Therefore, $$xy\in
								E(G)$$. Since $$x,y\in L(T(u))$$ and $$G_2$$ is the subgraph of $$G$$ induced by *L*(*T*(*u*)), we have $$xy\in
								E(G_2)$$ and thus $$E(G_1)\subseteq
								E(G_2)$$. Now assume $$xy\in E(G_2)$$ for some $$x,y\in L(T(u))$$. Hence, $$xy\in E(G)$$. Consequently, there is no leaf $$y'$$ in *T* with $$\sigma
								(y')=\sigma (y)\ne \sigma (x)$$ such that $${{\,\mathrm{lca}\,}}_{T}(x,y')\prec
								_{T}{{\,\mathrm{lca}\,}}_{T}(x,y)\preceq _{T}
								u$$. This clearly also holds for the subtree *T*(*u*). Therefore, we have $$xy\in E(G_1)$$ and thus $$E(G_2)\subseteq
								E(G_1)$$. $$\square $$

### Lemma 4

If $$(T,\sigma
								)$$ is least resolved for an *n*-cBMG $$(G,\sigma
								)$$, then the subtree *T*(*u*) is least resolved for the *n*-cBMG $$G(T(u),\sigma
								_{|L(T(u))})$$ for each $$u\in V(T)$$.

### Proof

The statement is trivially satisfied if *T*(*u*) does not contain any inner edges, which is exactly the case if either $$u\in L(T)$$ or $$u\in V^0(T)$$ with $$\mathsf {child}_T(u)\subseteq
								L(T)$$. Thus, let $$u\in V^0(T)$$ and $$\mathsf {child}_T(u)\cap V^0(T)\ne \emptyset
								$$. Since $$(T,\sigma )$$ is least resolved, it does not contain redundant edges. Let *vw* be an inner edge of *T*(*u*) with $$w\prec _T v\preceq _T
								u$$, and note that *vw* must also be an inner edge in *T*. By Lemma 2 and since *vw* is not redundant in *T*, there is an arc $$ab\in E(G)$$ such that $${{\,\mathrm{lca}\,}}_T(a,b)=w$$ and $$\sigma (b)\in \sigma (L(T(v))\setminus
								L(T(w)))$$. Since $$u\succeq _T v$$, Lemma 3 implies that *ab* is also an arc in $$G(T(u),\sigma
								_{|L(T(u))})$$ and $${{\,\mathrm{lca}\,}}_{T(u)}(a,b)=v$$. Hence, in particular, we have $$\sigma (b)\in
								\sigma (L(T(v))\setminus
								L(T(w)))$$. We can now apply Lemma 2 to conclude that *vw* is not redundant in *T*(*u*). Since *vw* was chosen arbitrarily, we conclude that *T*(*u*) does not contain any redundant edge and thus, it must be least resolved for $$G(T(u),\sigma
								_{|L(T(u))})$$ for all $$u\in V(T)$$. $$\square $$

We finally relate the subtrees *T*(*u*) to the construction of the Aho-graph as specified in (Geiß et al. (2019), Sec. 3.4). Given a set of triples *R* on *L*, we will write $$R_{|L'}$$ for the set of triples $$ab|c\in R$$ with $$a,b,c\in L'\subseteq
								L$$.

### Lemma 5

Let $$(T,\sigma
								)$$ be least resolved for an *n*-cBMG $$(G,\sigma
								)$$ with informative triple set $${\mathcal
								{R}}{:}{=}{\mathcal {R}}(G,\sigma
								)$$. Then, *L*(*T*(*v*)) is a connected component in the Aho-graph $$[{\mathcal
								{R}}_{|L(T(u))}, L(T(u))]$$ for every inner vertex *u* and each of its children $$v\in \mathsf
								{child}_{T}(u)$$.

### Proof

We proceed by induction on $L {:}{=}V(G)$. The statement trivially holds for $|L|=1$. Hence, suppose that $|L|>1$ and assume that the statement is true for every *n*-cBMG with less than |*L*| vertices.

Let *u* be an inner vertex of *T* and $$v\in \mathsf
								{child}_{T}(u)$$. We first show that *L*(*T*(*v*)) is connected in $$[{\mathcal
								{R}}_{|L(T(u))}, L(T(u))]$$, and then argue that there are no edges between *L*(*T*(*v*)) and $$L(T(u))\setminus
								L(T(v))$$, i.e., that *L*(*T*(*v*)) forms a connected component.

If *uv* is an outer edge, i.e. *v* is a leaf, then *L*(*T*(*v*)) is trivially connected. Now suppose that *uv* is an inner edge of *T*. By Lemmas 3 and 4, $$(G[L(T(v))],\sigma
								_{|L(T(v))})$$ is explained by the least resolved tree $$(T(v), \sigma
								_{|L(T(v))})$$. By the induction hypothesis, *L*(*T*(*w*)) forms a connected component in $$[{\mathcal
								{R}}_{|L(T(v))}, L(T(v))]$$ for all children $$w\in \mathsf
								{child}_T(v)$$. Together with $${\mathcal {R}}_{|L(T(v))}\subseteq {\mathcal
								{R}}_{|L(T(u))}$$, this implies that the elements in *L*(*T*(*w*)) are also connected in $$[{\mathcal
								{R}}_{|L(T(u))}, L(T(u))]$$ for all $$w\in \mathsf
								{child}_T(v)$$. Since *uv* is an inner edge of the least resolved tree $$(T,\sigma )$$, we can apply Corollary 1 to conclude that there is an informative triple $$ab|b'$$ in $$(G,\sigma )$$ that distinguishes *uv*, i.e. $${{\,\mathrm{lca}\,}}_T(a,b)=v$$ and $$b'\in L(T(u))\setminus
								L(T(v))$$ with color $$\sigma (b')=\sigma
								(b)$$. Hence, $$ab|b'$$ is also contained in $$[{\mathcal {R}}_{|L(T(u))},
								L(T(u))]$$. In particular, there are children $$w,w'\in \mathsf
								{child}_T(v)$$ such that $$a\preceq _T w$$ and $$b\preceq _T w'$$, and the edge *ab* connects *L*(*T*(*w*)) and $$L(T(w'))$$ in $$[{\mathcal {R}}_{|L(T(u))},
								L(T(u))]$$.

Now suppose that there is an additional child $$w''\in \mathsf {child}_T(v)\setminus
								\{w,w'\}$$. We distinguish two cases. Either there is a leaf $$b''\preceq _T
								w''$$ with $$\sigma (b'')=\sigma
								(b)$$ or no such leaf exists. If there is such a leaf $$b''$$, then $$ab''$$ forms an arc in $$(G,\sigma )$$ and $$ab''|b'$$ is an informative triple making *L*(*T*(*w*)) and $$L(T(w''))$$ connected in $$[{\mathcal {R}}_{|L(T(u))},
								L(T(u))]$$. Otherwise, take an arbitrary leaf $$c\preceq _T
								w''$$. Since $$\sigma (b)\notin \sigma
								(L(T(w'')))$$, we have $$\sigma (c)\ne \sigma
								(b)$$ and thus, there is an arc *cb* in $$(G,\sigma
								)$$. Since $${{\,\mathrm{lca}\,}}_T(c,b') = u \succ _T v =
								{{\,\mathrm{lca}\,}}_T(c,b)$$, the arc $$cb'$$ is not contained in $$(G,\sigma )$$. Hence, $$cb|b'$$ is an informative triple making $$L(T(w'))$$ and $$L(T(w''))$$ connected in $$[{\mathcal {R}}_{|L(T(u))},
								L(T(u))]$$.

Therefore, the subgraph in $$[{\mathcal {R}}_{|L(T(u))},
								L(T(u))]$$ induced by *L*(*T*(*v*)) must be connected.

It remains to show that *L*(*T*(*v*)) is a connected component in $$[{\mathcal
								{R}}_{|L(T(u))}, L(T(u))]$$ and thus, that there are no edges *ab* in $$[{\mathcal
								{R}}_{|L(T(u))}, L(T(u))]$$ with $$a\in L(T(v))$$ and $$b\in L(T(u))\setminus
								L(T(v))$$. Assume, for contradiction, that there exists such an edge *ab*. Hence, this edge must be supported by an informative triple w.l.o.g. $$ab|b'$$ with $$\sigma (a)\ne \sigma (b)=\sigma
								(b')$$ and $$b'\in L(T(u))$$. Lemma  1 implies that $$ab|b'$$ must be displayed by *T*. However, $${{\,\mathrm{lca}\,}}_T(a,b) = u =
								{{\,\mathrm{lca}\,}}_T(a,b,b')$$ implies that such a triple cannot exist. Thus, *L*(*T*(*v*)) is a connected component in $$[{\mathcal
								{R}}_{|L(T(u))}, L(T(u))]$$. $$\square $$

The least resolved tree of an *n*-cBMG therefore coincides with the Aho tree of its informative triples. In more detail, we have

### Proposition 1

If $$(G,\sigma
								)$$ is an *n*-cBMG, then $$({{\,\mathrm{Aho}\,}}({\mathcal {R}}(G,\sigma
								)),\sigma )$$ is the unique least resolved tree for $$(G,\sigma
								)$$.

### Proof

Since $$(G,\sigma
								)$$ is an *n*-cBMG, Lemma 1 implies that there is a tree displaying all triples in $${\mathcal {R}}(G,\sigma
								)$$. In particular, therefore, $${{\,\mathrm{Aho}\,}}({\mathcal {R}}(G,\sigma
								))$$ exists. Moreover, there must be a least resolved tree $$(T^*,\sigma
								)$$ for $$(G,\sigma )$$. To see this, consider an arbitrary tree $$(T,\sigma
								)$$ that explains $$(G,\sigma )$$, and repeatedly identify and contract a redundant edge until no redundant edges remain. By definition, the resulting tree still explains $$(G,\sigma
								)$$ and is least resolved. By Lemma 5 and by construction of $$({{\,\mathrm{Aho}\,}}({\mathcal {R}}(G,\sigma
								)),\sigma )$$, any least resolved tree $$(T^*,\sigma )$$ for $$(G,\sigma )$$ coincides with the latter. The uniqueness of $${{\,\mathrm{Aho}\,}}({\mathcal {R}}(G,\sigma
								))$$ therefore implies that the least resolved tree is also unique.$$\square
								$$

We now have all the pieces in place to complete the proof of the main result:

### Proof of Theorem 1

If $$(G,\sigma
								)$$ is an *n*-cBMG, then Proposition 1 implies that $$({{\,\mathrm{Aho}\,}}({\mathcal {R}}(G,\sigma
								)),\sigma )$$ is its unique least resolved tree, and thus $$G({{\,\mathrm{Aho}\,}}({\mathcal {R}}(G,\sigma
								)),\sigma ) = (G,\sigma )$$. Conversely, $$G({{\,\mathrm{Aho}\,}}({\mathcal {R}}(G,\sigma
								)),\sigma )$$ is an *n*-cBMG. $$\square
								$$

None of the intermediate results used to prove Theorem 9 in Geiß et al. (2019) is used below in our proof of Theorem 1. However, to the best of our knowledge, all results in Geiß et al. (2019) with the exception of the aforementioned Lemmas 9 and 11, and Thms. 4 and 9 are correct as stated. It is worth noting, furthermore, that Theorem 1 immediately implies Thms. 5, 6, and 7, as well as the existence of a unique least resolved tree in Thms. 2 and 8 of Geiß et al. (2019). In particular, Theorem 1 allows us to obtain the least resolved tree of an *n*-cBMG without the need to explicitly construct the least resolved trees of all its 2-colored induced subgraphs.

To prove the correctness of the amended version of Theorem 9, it only remains to show

### Proposition 2

If $$(G,\sigma
								)$$ is a *n*-cBMG, then $${{\,\mathrm{Aho}\,}}({\mathcal {R}}(G,\sigma
								))={{\,\mathrm{Aho}\,}}(R)$$.

### Proof

For brevity set $${\mathcal
								{R}}{:}{=}{\mathcal {R}}(G,\sigma
								)$$. From Eq. (1), i.e., $${\mathcal {R}}\subseteq
								R$$, we immediately have $${\mathcal {R}}_{|L(T(u))}\subseteq
								R_{|L(T(u))}$$ for every inner vertex *u* of *T*. Moreover, by Theorem 1, $$(T,\sigma
								)$$ with $$T{:}{=}{{\,\mathrm{Aho}\,}}({\mathcal
								{R}})$$ is the least resolved tree that explains $$(G,\sigma
								)$$.

Hence, we can apply the same arguments as in the proof of Lemma 5 to conclude that *L*(*T*(*v*)) forms a connected component in the Aho-graph $$[R_{|L(T(u))},
								L(T(u))]$$ for every inner vertex *u* and each of its children $$v\in \mathsf
								{child}_{T}(u)$$. More precisely, note that connectedness of any such *L*(*T*(*v*)) is guaranteed by the informative triples. Now assume, for contradiction, that there is an edge *ab* in $$[R_{|L(T(u))},
								L(T(u))]$$ with $$a\in L(T(v))$$ and $$b\in L(T(u))\setminus
								L(T(v))$$ connecting *L*(*T*(*v*)) and $$L(T(v'))$$ for some child $$v'\in \mathsf {child}_T(u)\setminus
								\{v\}$$. In this case, there is a triple $$ab|c\in
								R_{|L(T(u))}$$ and thus, $$a,b,c\in
								L(T(u))$$ and $${{\,\mathrm{lca}\,}}_T(a,b,c)=u$$. By definition of *R* and Observation 4 in Geiß et al. (2019), *ab*|*c* must be displayed by *T*. However, $$a,b,c\in
								L(T(u))$$ and $${{\,\mathrm{lca}\,}}_{T}(a,b)=u
								={{\,\mathrm{lca}\,}}_{T}(a,b,c)$$ imply that *ab*|*c* is not displayed by *T*; a contradiction. Therefore, $$(T,\sigma )=({{\,\mathrm{Aho}\,}}(R),\sigma
								)$$, which completes the proof. $$\square
								$$

For completeness, we show that conditions (i) and (ii) of Theorem 9 ensure that $$G({{\,\mathrm{Aho}\,}}(R),\sigma
								)$$ and $$G({{\,\mathrm{Aho}\,}}({\mathcal {R}}(G,\sigma
								)),\sigma )$$ are subgraphs of $$(G,\sigma )$$.

### Proposition 3

Let $$(G,\sigma
								)$$ be a properly *n*-colored digraph with all 2-colored induced subgraphs being 2-cBMGs. Then the following two statements hold:

1. If $${\mathcal {R}}(G,\sigma
											)$$ is consistent, then $$G({{\,\mathrm{Aho}\,}}({\mathcal
											{R}}(G,\sigma )),\sigma )\subseteq (G,\sigma
											)$$.
2. If *R* is consistent, then $$G({{\,\mathrm{Aho}\,}}(R),\sigma
											)\subseteq (G,\sigma
											)$$.

### Proof

We set $$(G',\sigma
								'){:}{=}G({{\,\mathrm{Aho}\,}}({\mathcal {R}}(G,\sigma )),\sigma
								)$$. Since $${{\,\mathrm{Aho}\,}}({\mathcal {R}}(G,\sigma
								))$$ is defined on *V*(*G*), we have $$V(G')= V(G)$$ and $$\sigma '=\sigma
								$$. Now assume, for contradiction, that there is an arc $$ab\in
								E(G')$$ such that $$ab\notin E(G)$$. By assumption, the induced subgraph $$(G_{st},\sigma
								_{st})$$ of $$(G,\sigma )$$, where $$s=\sigma (a)$$ and $$t=\sigma (b)$$, is a 2-cBMG and thus sink-free. Therefore, there must be a vertex $$b'$$ of color $$\sigma (b)$$ with $$ab'\in E(G)$$. Hence, $$ab'|b$$ is informative for $$(G,\sigma )$$ and contained in $${\mathcal {R}}(G,\sigma
								)$$. In particular, $$ab'|b$$ must be displayed by $${{\,\mathrm{Aho}\,}}({\mathcal {R}}(G,\sigma
								))$$; contradicting that *ab* is an arc in $$(G',\sigma
								')$$. Hence, statement (i) is true.

Statement (ii) can be shown using Eq. (1), i.e., $${\mathcal {R}}(G,\sigma )\subseteq
								R$$, and arguments similar to the previous paragraph. $$\square
								$$

## Consequences for the algorithms

Finally, we discuss the consequences of the corrections for the algorithmic aspects outlined in Section 5 of Geiß et al. (2019).

Algorithm 2 constructs the least resolved tree for 2-cBMGs based on Theorem 4 in Geiß et al. (2019). It therefore requires a sink-free graph as input, or needs to be amended to check that its input satisfies condition (N4). This can be done trivially in *O*(|*E*|) time. The statements concerning its complexity, i.e., Lemmas 18 and 19, therefore are still correct.

Regarding the recognition of *n*-cBMGs, we have noted above that the consistency of the triple set *R* and the fact that all 2-colored induced subgraphs are 2-BMGs are not sufficient. Algorithm 1 of Geiß et al. (2019) therefore also needs to be corrected. By Theorem 1, it suffices to construct the tree $$T{:}{=}{{\,\mathrm{Aho}\,}}({\mathcal {R}}(G,\sigma
							))$$ and to check whether $$G(T,\sigma ) = (G,\sigma
							)$$. On the other hand, it is no longer necessary to require connectedness of the input graph. We therefore obtain a considerably simpler procedure, see Alg. 1.
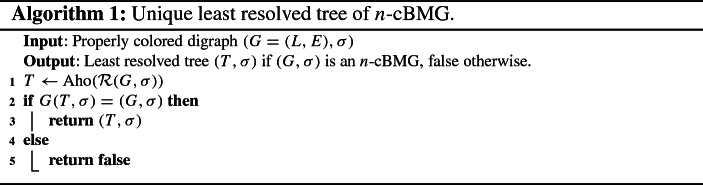


The same arguments as in Geiß et al. (2019) show that $$T={{\,\mathrm{Aho}\,}}({\mathcal {R}}(G,\sigma
							))$$ can be constructed in $$O(|E||L|\log ^2(|E||L|)) {=O(|E||L|\log
							^2|L|)}$$ time using the algorithm by Deng and Fernández-Baca (2018). The construction of $$G(T,\sigma
							)$$ can then be achieved in $$O(|L|^2)$$ time e.g. using Algorithm 1 of the Supplement of Geiß et al. (2020). The equality $$G(T,\sigma
							)=(G,\sigma )$$ can be checked in $$O(|L|^2)$$ operations. The total effort therefore remains dominated by the construction of the least resolved tree *T*.

We note that Algorithm 3 in Geiß et al. (2019) is essentially the simplified Algorithm 1 above with its input restricted to 2-colored connected digraphs. Its correctness therefore follows immediately from Theorem 1.
